# Supplementary material for: Multimodal Imaging Techniques to Evaluate the Anticancer Effect of Cold Atmospheric Pressure Plasma
Source: Cancers (Basel). 2021 May 19;13(10):2483. doi: 10.3390/cancers13102483 (PMC8161248; doi:10.3390/cancers13102483)
Supplement: Supplementary file 1 [file cancers-13-02483-s001.zip › cancers-1174396-supplementary/Figure S1_Quantitative calliper assessment of tumour volumes (A and B).pdf]

Supplementary Materials:

Multimodal Imaging Techniques to Evaluate the Anticancer Effect of Cold Atmospheric Pressure Plasma

Marcel Kordt, Isabell Trautmann, Christin Schlie, Tobias Lindner, Jan Stenzel, Anna Schildt, Lars Boeckmann, Sander Bekeschus, Jens Kurth, Bernd J. Krause, Brigitte Vollmar and Eberhard Grambow

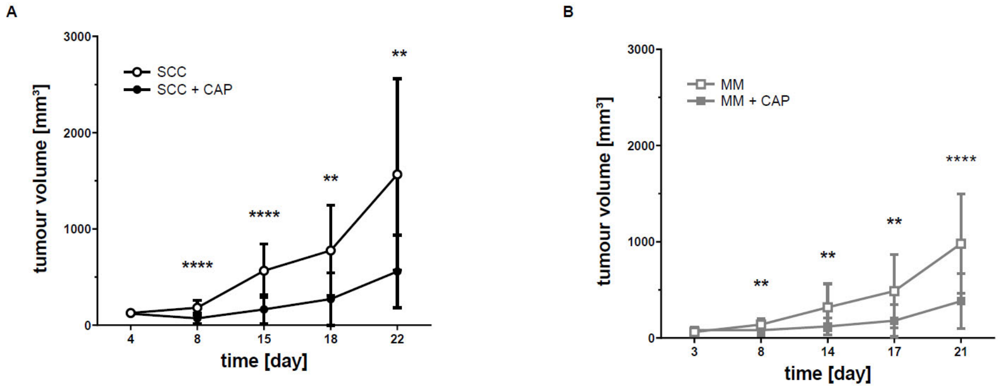

Figure S1. Quantitative calliper assessment of tumour volumes (A and B). NSG mice with s.c. flank tumours, either squamous cell carcinoma (SCC) or malignant melanoma (MM), were treated with or without cold atmospheric pressure plasma (CAP) over three weeks. CAP treatment started four days after tumour cell injection and was repeated every four days. CAP treatment caused a significant reduction of both SCC and MM growth over the three-week observation time. Empty symbols represent untreated tumours, and filled symbols represent CAP-treated tumours. Data are presented as mean ± SD (n = 20–24 samples per time point); two-way ANOVA followed by Bonferroni correction for multiple comparison. **$p \leq 0.01$;****$p \leq 0.0001$ vs. untreated SCC or MM, respectively.
