# Supplementary material for: Multimodal Imaging Techniques to Evaluate the Anticancer Effect of Cold Atmospheric Pressure Plasma
Source: Cancers (Basel). 2021 May 19;13(10):2483. doi: 10.3390/cancers13102483 (PMC8161248; doi:10.3390/cancers13102483)
Supplement: Supplementary file 1 [file cancers-13-02483-s001.zip › cancers-1174396-supplementary/Figure S2. Original Western Blot images/MM cell line A375 cleaced-caspase-3 ß-actin.pdf]

Image Report: 20200702 Cleaved Caspase 3 Eva S4 60µg Prot

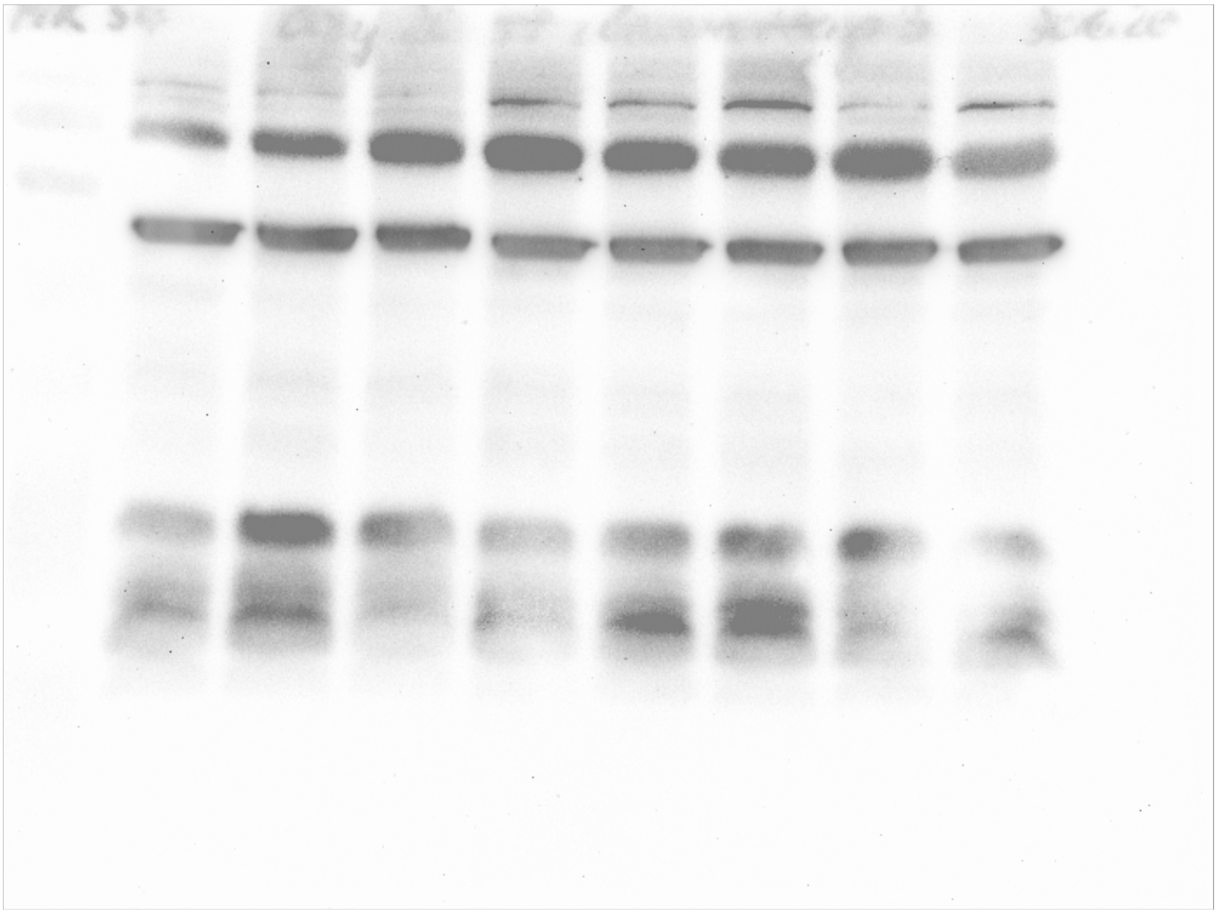

C:\Users\marcel.kordt\Desktop\Marcel\Versuche\Probenaufbereitung\Mol\WB März 2020 mit und ohne KAP\Auswertung Finall\20200702 Cleaved Caspase 3 Eva S4 60µg Prot.mscn

---

Channel 1 - Red - Chemi Hi Resolution

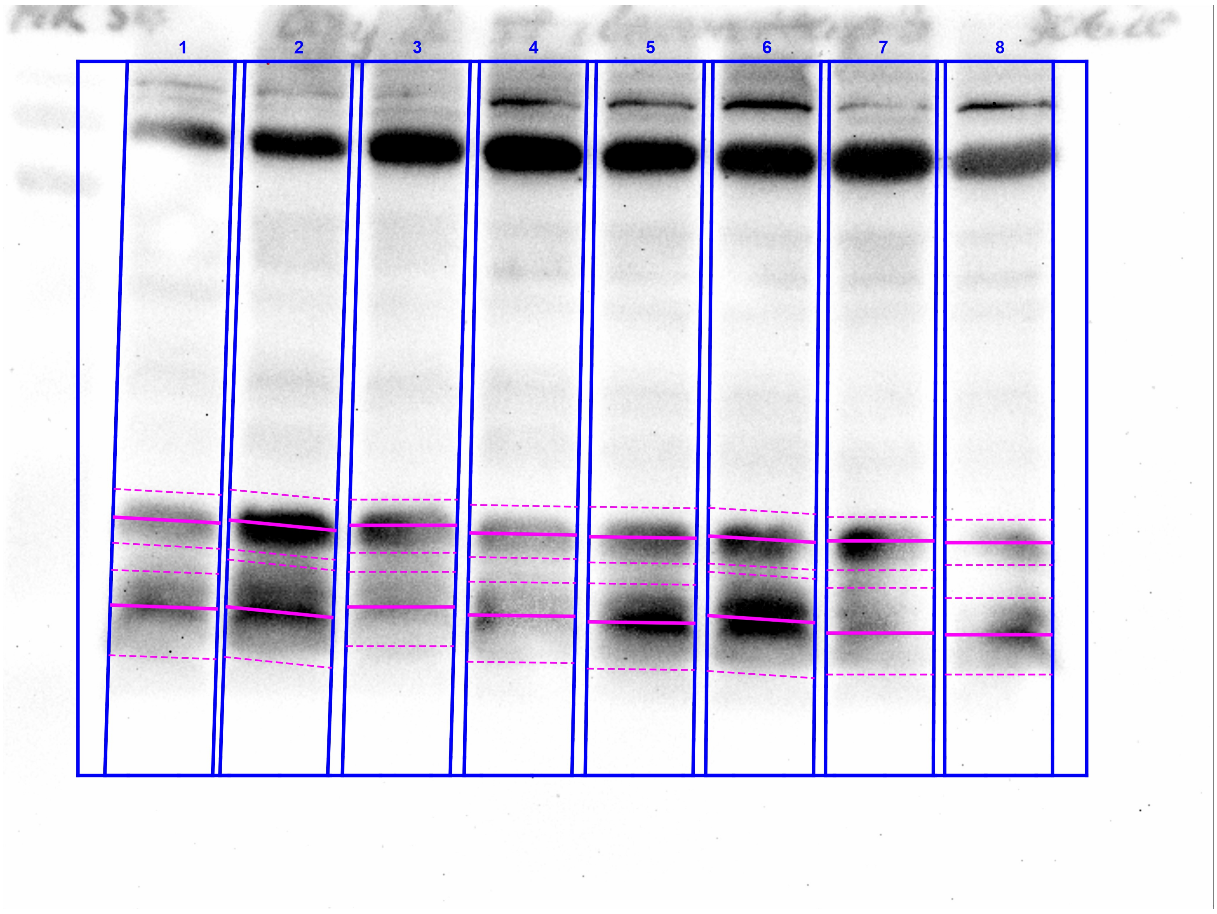

Lane Statistics

Channel	Lane No.	Adj. Total Band Vol. (Int)	Total Band Vol. (Int)	Adj. Total Lane Vol. (Int)	Total Lane Vol. (Int)	Bkgd. Vol. (Int)	Norm. Factor
Chemi Hi Resolution	1	30.817.348	73.927.064	56.931.128	228.837.288	171.906.160	N/A
Chemi Hi Resolution	2	45.965.932	114.564.406	77.347.046	300.225.886	222.878.840	N/A
Chemi Hi Resolution	3	21.824.682	70.568.586	61.785.976	270.167.728	208.381.752	N/A
Chemi Hi Resolution	4	22.919.788	62.503.254	66.760.050	278.388.618	211.628.568	N/A
Chemi Hi Resolution	5	39.944.182	88.892.252	78.055.458	289.210.718	211.155.260	N/A
Chemi Hi Resolution	6	44.602.676	104.691.588	83.875.026	304.580.146	220.705.120	N/A
Chemi Hi Resolution	7	25.077.388	70.181.272	64.248.678	265.270.286	201.021.608	N/A
Chemi Hi Resolution	8	26.364.136	54.326.384	59.236.536	229.783.780	170.547.244	N/A

Lane And Band Analysis

Lane 1

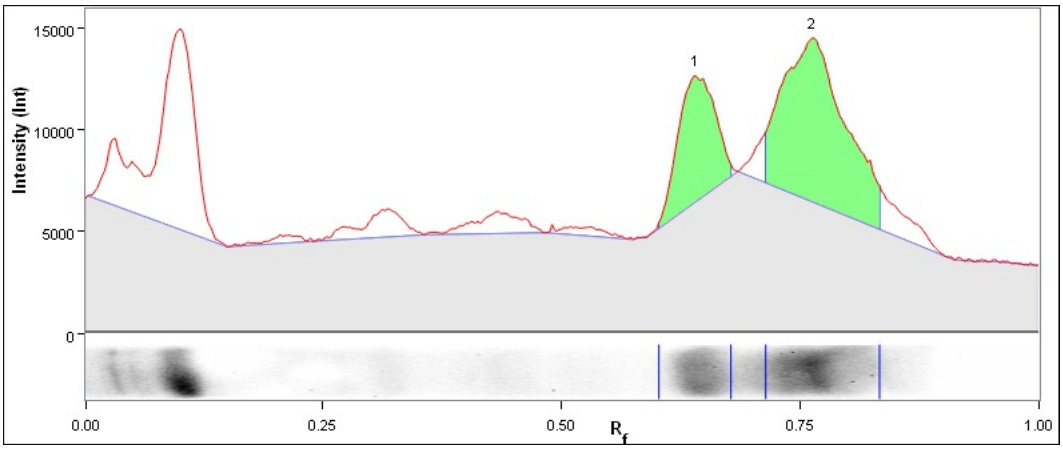

Channel	Band No.	Band Label	Mol. Wt. (KDa)	Relative Front	Adj. Volume (Int)	Volume (Int)	Abs. Quant.	Rel. Quant.	Band %	Lane %	Norm. Factor	Norm. Vol. (Int)
Chemi Hi Resolution	1		N/A	0,642	9.586.440	27.086.808	N/A	N/A	31,1	16,8	N/A	N/A
Chemi Hi Resolution	2		N/A	0,764	21.230.908	46.840.256	N/A	N/A	68,9	37,3	N/A	N/A

Band Detection	Automatically detected bands with sensitivity: Low
Lane Background	Lane background subtracted with disk size: 10
Lane Width	7.39 mm

Lane 2

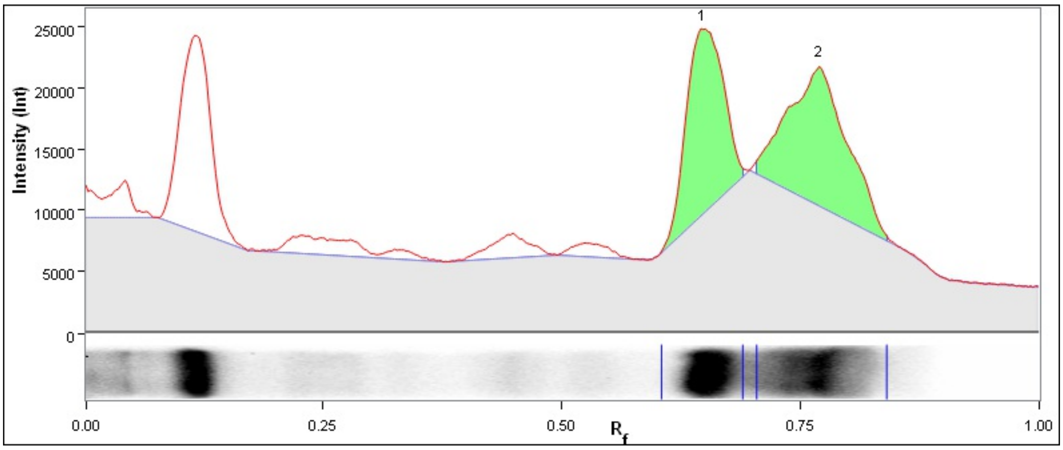

Channel	Band No.	Band Label	Mol. Wt. (KDa)	Relative Front	Adj. Volume (Int)	Volume (Int)	Abs. Quant.	Rel. Quant.	Band %	Lane %	Norm. Factor	Norm. Vol. (Int)
Chemi Hi Resolution	1		N/A	0,650	21.060.904	47.270.164	N/A	N/A	45,8	27,2	N/A	N/A
Chemi Hi Resolution	2		N/A	0,771	24.905.028	67.294.242	N/A	N/A	54,2	32,2	N/A	N/A

Band Detection	Automatically detected bands with sensitivity: Low
Lane Background	Lane background subtracted with disk size: 10
Lane Width	7.39 mm

Lane 3

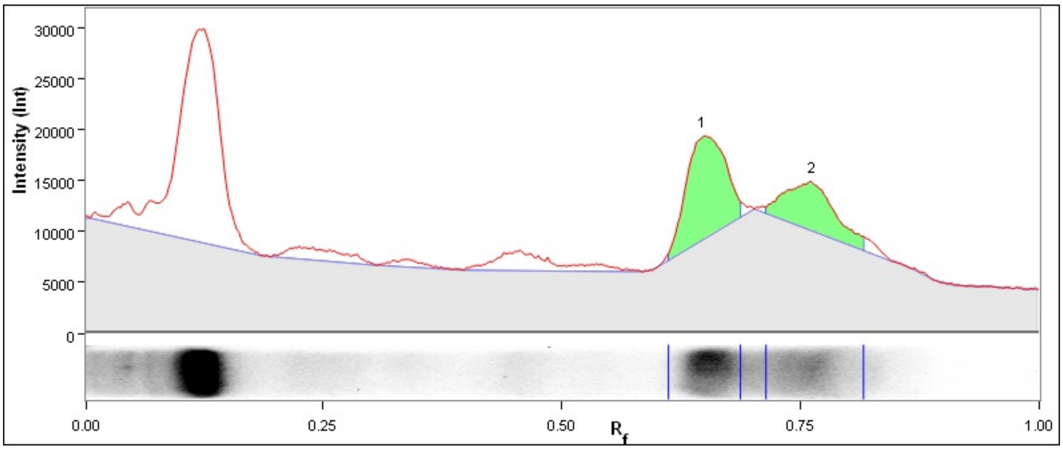

Channel	Band No.	Band Label	Mol. Wt. (KDa)	Relative Front	Adj. Volume (Int)	Volume (Int)	Abs. Quant.	Rel. Quant.	Band %	Lane %	Norm. Factor	Norm. Vol. (Int)
Chemi Hi Resolution	1		N/A	0,650	13.571.304	33.013.388	N/A	N/A	62,2	22,0	N/A	N/A
Chemi Hi Resolution	2		N/A	0,764	8.253.378	37.555.198	N/A	N/A	37,8	13,4	N/A	N/A

Band Detection	Automatically detected bands with sensitivity: Low
Lane Background	Lane background subtracted with disk size: 10
Lane Width	7.39 mm

Lane 4

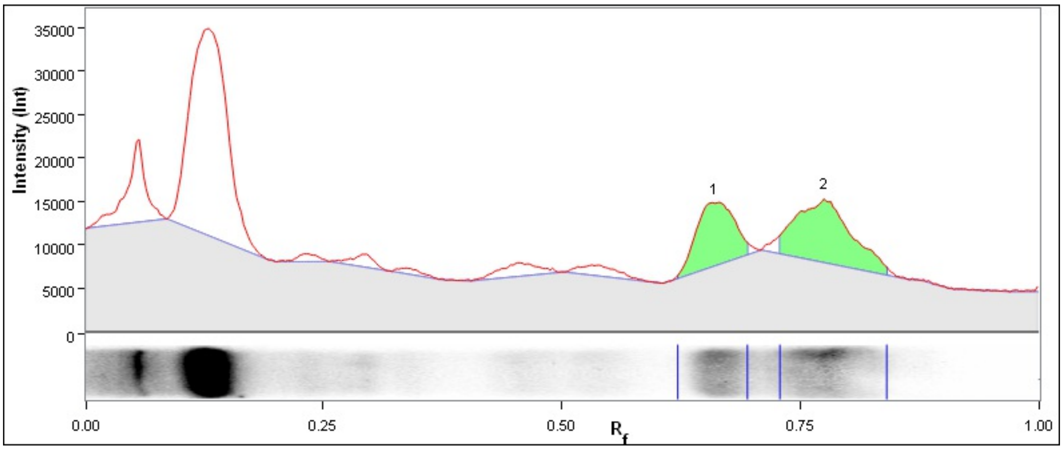

Channel	Band No.	Band Label	Mol. Wt. (KDa)	Relative Front	Adj. Volume (Int)	Volume (Int)	Abs. Quant.	Rel. Quant.	Band %	Lane %	Norm. Factor	Norm. Vol. (Int)
Chemi Hi Resolution	1		N/A	0,662	9.483.644	25.131.700	N/A	N/A	41,4	14,2	N/A	N/A
Chemi Hi Resolution	2		N/A	0,776	13.436.144	37.371.554	N/A	N/A	58,6	20,1	N/A	N/A

Band Detection	Automatically detected bands with sensitivity: Low
Lane Background	Lane background subtracted with disk size: 10
Lane Width	7.39 mm

Lane 5

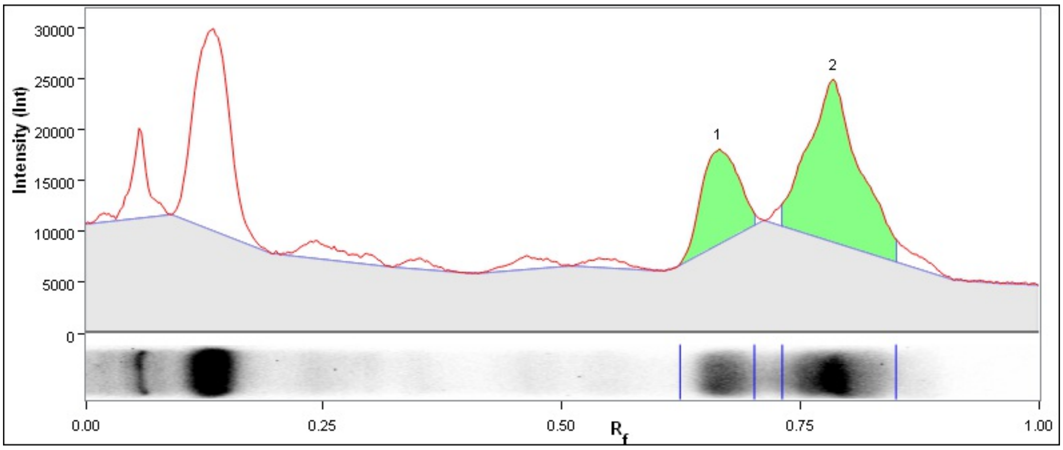

Channel	Band No.	Band Label	Mol. Wt. (KDa)	Relative Front	Adj. Volume (Int)	Volume (Int)	Abs. Quant.	Rel. Quant.	Band %	Lane %	Norm. Factor	Norm. Vol. (Int)
Chemi Hi Resolution	1		N/A	0,667	11.832.204	30.949.160	N/A	N/A	29,6	15,2	N/A	N/A
Chemi Hi Resolution	2		N/A	0,786	28.111.978	57.943.092	N/A	N/A	70,4	36,0	N/A	N/A

Band Detection	Automatically detected bands with sensitivity: Low
Lane Background	Lane background subtracted with disk size: 10
Lane Width	7.39 mm

Lane 6

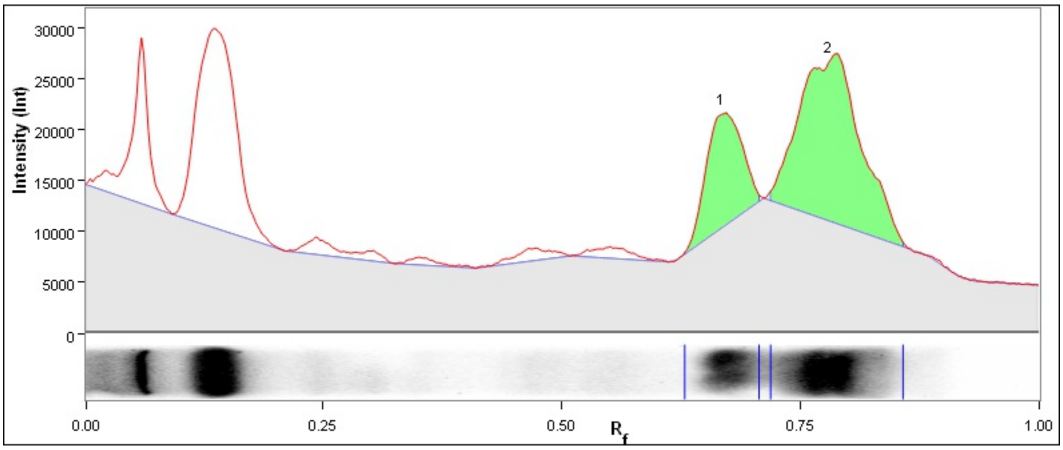

Channel	Band No.	Band Label	Mol. Wt. (KDa)	Relative Front	Adj. Volume (Int)	Volume (Int)	Abs. Quant.	Rel. Quant.	Band %	Lane %	Norm. Factor	Norm. Vol. (Int)
Chemi Hi Resolution	1		N/A	0,669	12.655.812	33.401.384	N/A	N/A	28,4	15,1	N/A	N/A
Chemi Hi Resolution	2		N/A	0,781	31.946.864	71.290.204	N/A	N/A	71,6	38,1	N/A	N/A

Band Detection	Automatically detected bands with sensitivity: Low
Lane Background	Lane background subtracted with disk size: 10
Lane Width	7.39 mm

Lane 7

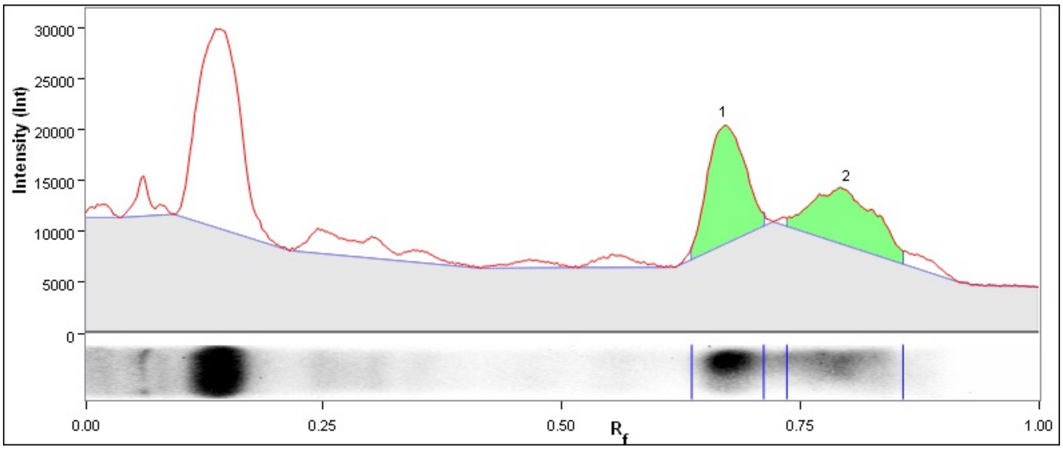

Channel	Band No.	Band Label	Mol. Wt. (KDa)	Relative Front	Adj. Volume (Int)	Volume (Int)	Abs. Quant.	Rel. Quant.	Band %	Lane %	Norm. Factor	Norm. Vol. (Int)
Chemi Hi Resolution	1		N/A	0,672	13.793.698	31.692.974	N/A	N/A	55,0	21,5	N/A	N/A
Chemi Hi Resolution	2		N/A	0,800	11.283.690	38.488.298	N/A	N/A	45,0	17,6	N/A	N/A

Band Detection	Automatically detected bands with sensitivity: Low
Lane Background	Lane background subtracted with disk size: 10
Lane Width	7.39 mm

Lane 8

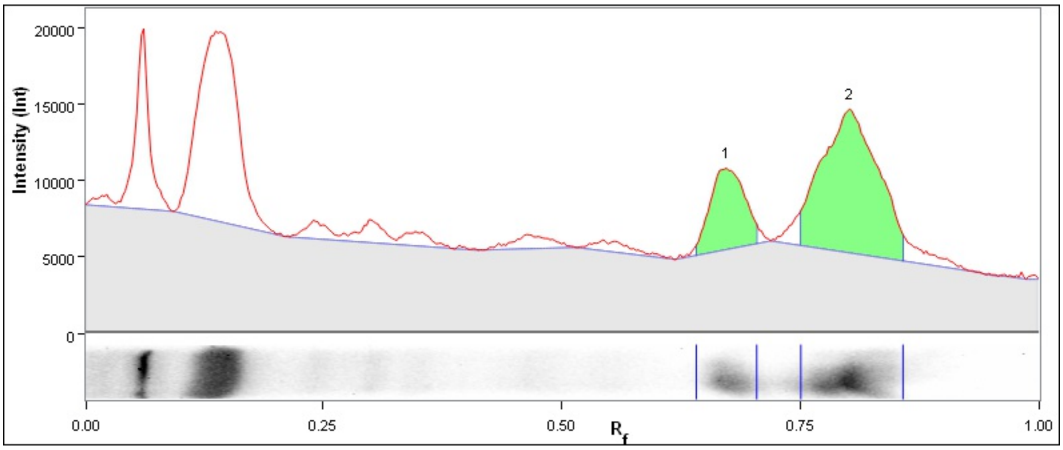

Channel	Band No.	Band Label	Mol. Wt. (KDa)	Relative Front	Adj. Volume (Int)	Volume (Int)	Abs. Quant.	Rel. Quant.	Band %	Lane %	Norm. Factor	Norm. Vol. (Int)
Chemi Hi Resolution	1		N/A	0,674	6.797.618	17.490.758	N/A	N/A	25,8	11,5	N/A	N/A
Chemi Hi Resolution	2		N/A	0,803	19.566.518	36.835.626	N/A	N/A	74,2	33,0	N/A	N/A

Band Detection	Automatically detected bands with sensitivity: Low
Lane Background	Lane background subtracted with disk size: 10
Lane Width	7.39 mm

Channel 2 - Green - Chemi Hi Resolution

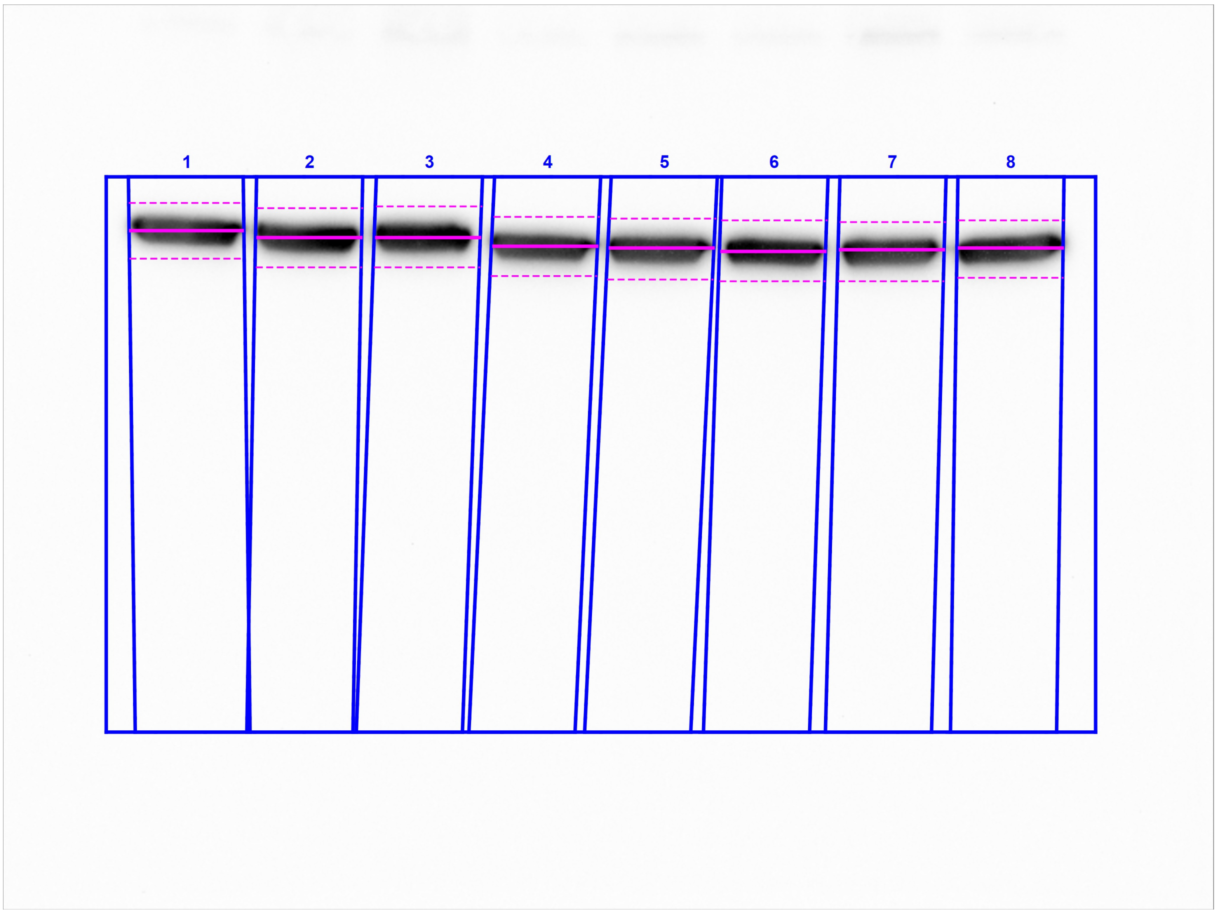

Lane Statistics

Channel	Lane No.	Adj. Total Band Vol. (Int)	Total Band Vol. (Int)	Adj. Total Lane Vol. (Int)	Total Lane Vol. (Int)	Bkgd. Vol. (Int)	Norm. Factor
Chemi Hi Resolution	1	41.463.774	43.873.038	42.815.916	64.953.834	22.137.918	N/A
Chemi Hi Resolution	2	44.633.029	47.088.828	45.967.465	66.956.284	20.988.819	N/A
Chemi Hi Resolution	3	44.827.070	47.396.451	46.211.221	67.386.700	21.175.479	N/A
Chemi Hi Resolution	4	37.700.196	40.126.654	39.068.670	60.266.109	21.197.439	N/A
Chemi Hi Resolution	5	39.181.337	41.703.077	40.591.779	61.940.620	21.348.841	N/A
Chemi Hi Resolution	6	41.820.380	44.299.359	43.383.810	64.448.086	21.064.276	N/A
Chemi Hi Resolution	7	37.942.915	40.363.578	39.359.091	60.425.075	21.065.984	N/A
Chemi Hi Resolution	8	40.062.909	42.341.076	41.363.368	61.878.522	20.515.154	N/A

Lane And Band Analysis

Lane 1

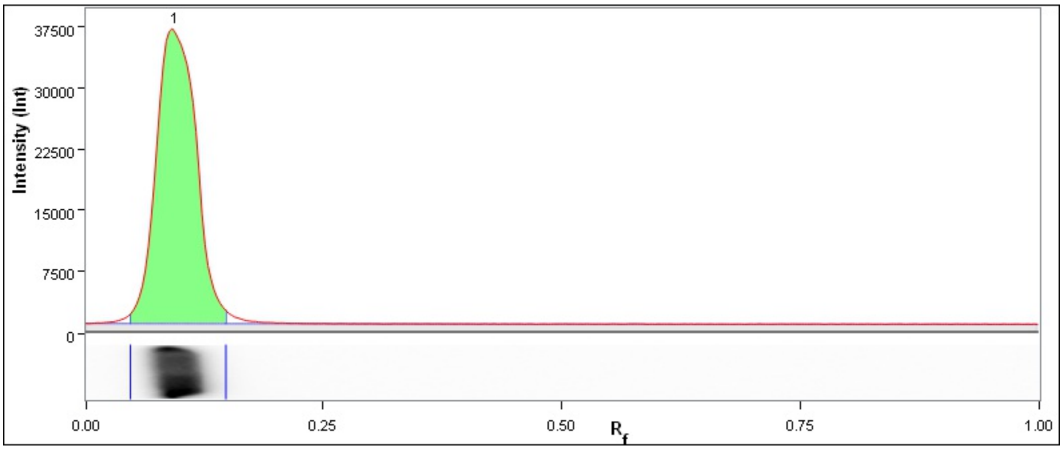

Channel	Band No.	Band Label	Mol. Wt. (KDa)	Relative Front	Adj. Volume (Int)	Volume (Int)	Abs. Quant.	Rel. Quant.	Band %	Lane %	Norm. Factor	Norm. Vol. (Int)
Chemi Hi Resolution	1		N/A	0,097	41.463.774	43.873.038	N/A	N/A	100,0	96,8	N/A	N/A

Band Detection	Automatically detected bands with sensitivity: Low
Lane Background	Lane background subtracted with disk size: 10
Lane Width	7.87 mm

Lane 2

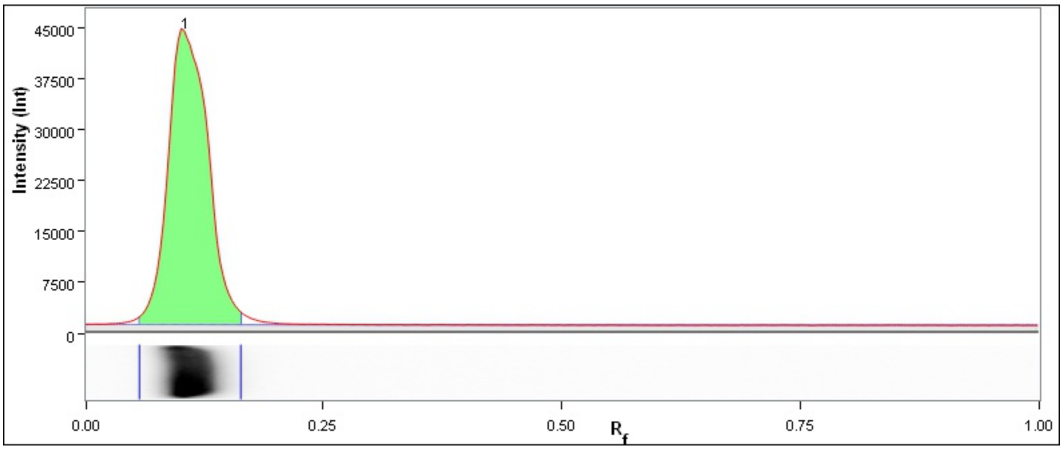

Channel	Band No.	Band Label	Mol. Wt. (KDa)	Relative Front	Adj. Volume (Int)	Volume (Int)	Abs. Quant.	Rel. Quant.	Band %	Lane %	Norm. Factor	Norm. Vol. (Int)
Chemi Hi Resolution	1		N/A	0,109	44.633.029	47.088.828	N/A	N/A	100,0	97,1	N/A	N/A

Band Detection	Automatically detected bands with sensitivity: Low
Lane Background	Lane background subtracted with disk size: 10
Lane Width	7.27 mm

Lane 3

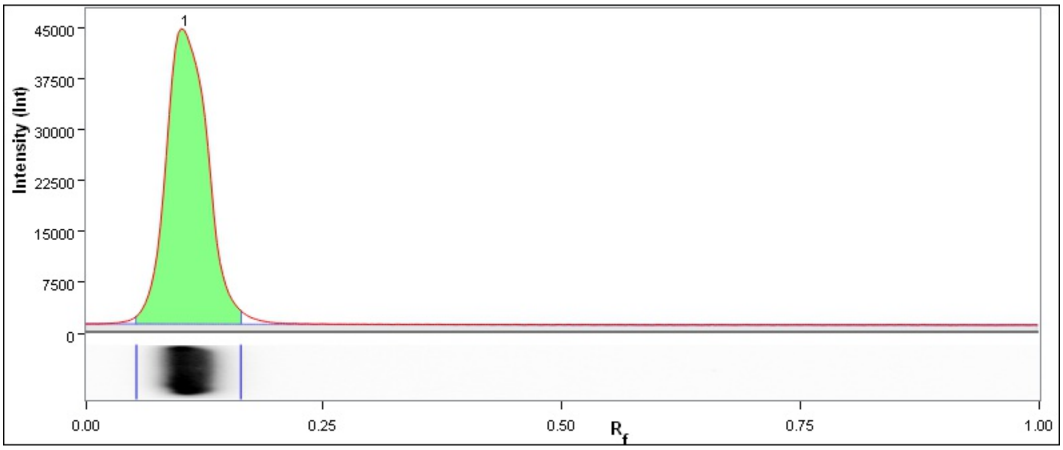

Channel	Band No.	Band Label	Mol. Wt. (KDa)	Relative Front	Adj. Volume (Int)	Volume (Int)	Abs. Quant.	Rel. Quant.	Band %	Lane %	Norm. Factor	Norm. Vol. (Int)
Chemi Hi Resolution	1		N/A	0,109	44.827.070	47.396.451	N/A	N/A	100,0	97,0	N/A	N/A

Band Detection	Automatically detected bands with sensitivity: Low
Lane Background	Lane background subtracted with disk size: 10
Lane Width	7.27 mm

Lane 4

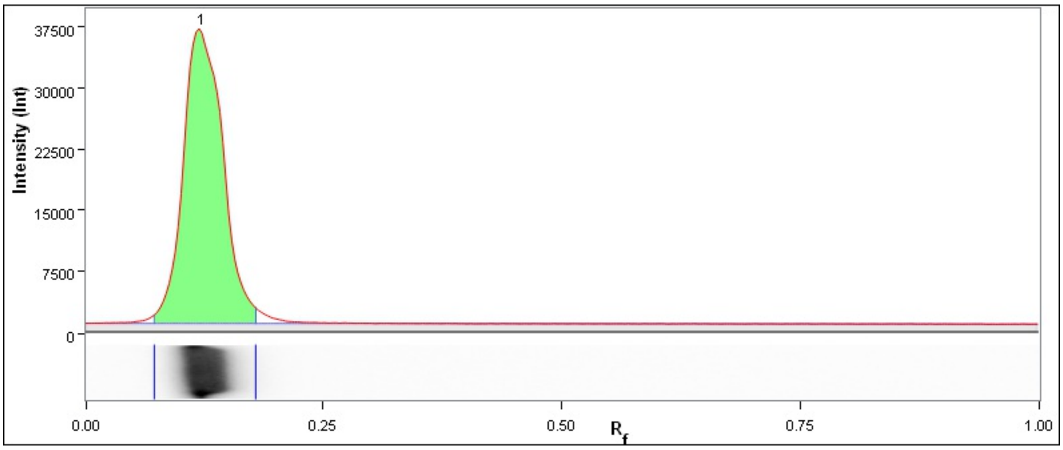

Channel	Band No.	Band Label	Mol. Wt. (KDa)	Relative Front	Adj. Volume (Int)	Volume (Int)	Abs. Quant.	Rel. Quant.	Band %	Lane %	Norm. Factor	Norm. Vol. (Int)
Chemi Hi Resolution	1		N/A	0,125	37.700.196	40.126.654	N/A	N/A	100,0	96,5	N/A	N/A

Band Detection	Automatically detected bands with sensitivity: Low
Lane Background	Lane background subtracted with disk size: 10
Lane Width	7.27 mm

Lane 5

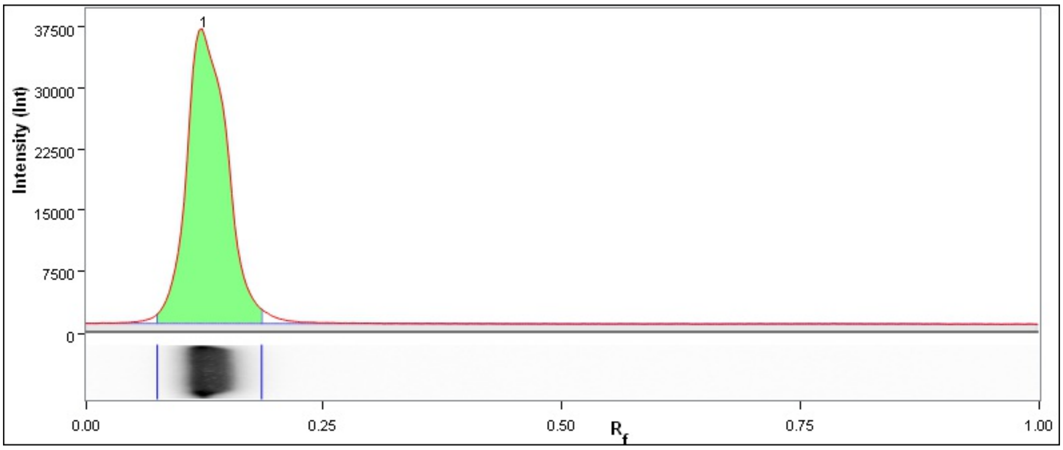

Channel	Band No.	Band Label	Mol. Wt. (KDa)	Relative Front	Adj. Volume (Int)	Volume (Int)	Abs. Quant.	Rel. Quant.	Band %	Lane %	Norm. Factor	Norm. Vol. (Int)
Chemi Hi Resolution	1		N/A	0,128	39.181.337	41.703.077	N/A	N/A	100,0	96,5	N/A	N/A

Band Detection	Automatically detected bands with sensitivity: Low
Lane Background	Lane background subtracted with disk size: 10
Lane Width	7.27 mm

Lane 6

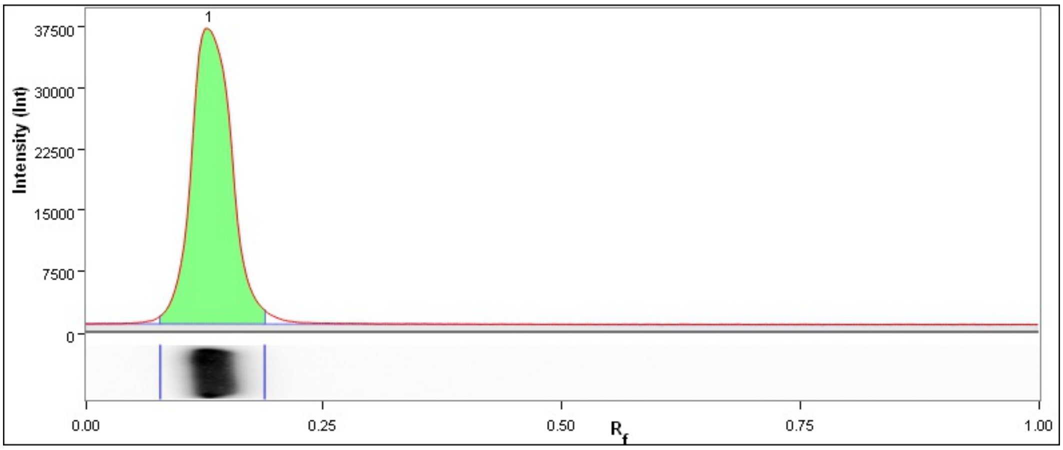

Channel	Band No.	Band Label	Mol. Wt. (KDa)	Relative Front	Adj. Volume (Int)	Volume (Int)	Abs. Quant.	Rel. Quant.	Band %	Lane %	Norm. Factor	Norm. Vol. (Int)
Chemi Hi Resolution	1		N/A	0,134	41.820.380	44.299.359	N/A	N/A	100,0	96,4	N/A	N/A

Band Detection	Automatically detected bands with sensitivity: Low
Lane Background	Lane background subtracted with disk size: 10
Lane Width	7.27 mm

Lane 7

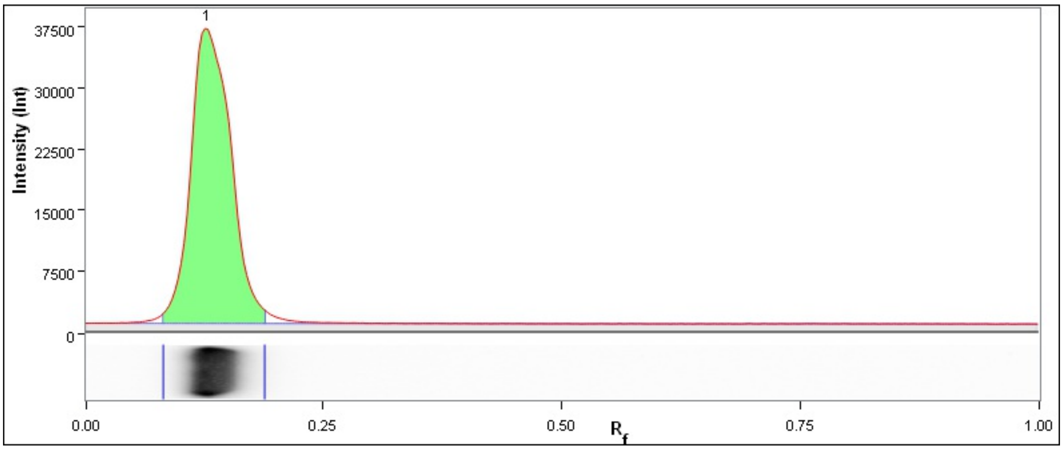

Channel	Band No.	Band Label	Mol. Wt. (KDa)	Relative Front	Adj. Volume (Int)	Volume (Int)	Abs. Quant.	Rel. Quant.	Band %	Lane %	Norm. Factor	Norm. Vol. (Int)
Chemi Hi Resolution	1		N/A	0,131	37.942.915	40.363.578	N/A	N/A	100,0	96,4	N/A	N/A

Band Detection	Automatically detected bands with sensitivity: Low
Lane Background	Lane background subtracted with disk size: 10
Lane Width	7.27 mm

Lane 8

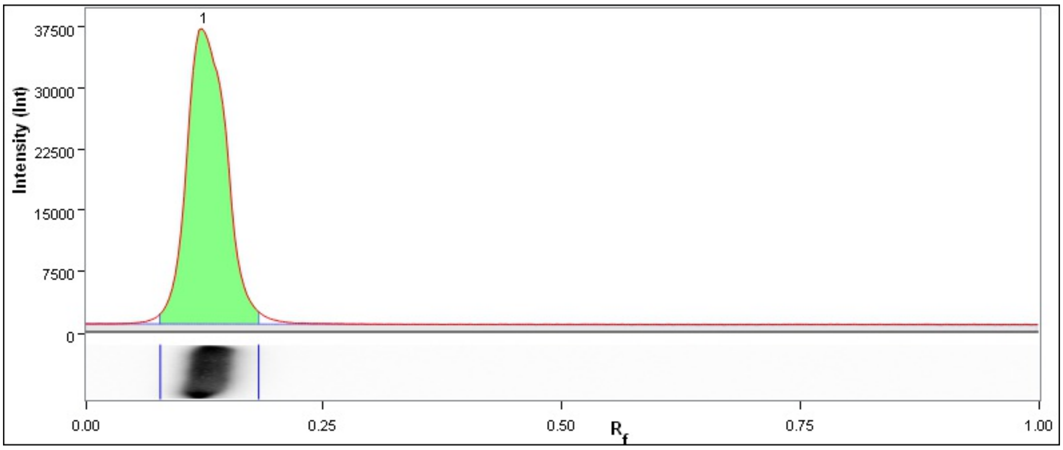

Channel	Band No.	Band Label	Mol. Wt. (KDa)	Relative Front	Adj. Volume (Int)	Volume (Int)	Abs. Quant.	Rel. Quant.	Band %	Lane %	Norm. Factor	Norm. Vol. (Int)
Chemi Hi Resolution	1		N/A	0,128	40.062.909	42.341.076	N/A	N/A	100,0	96,9	N/A	N/A

Band Detection	Automatically detected bands with sensitivity: Low
Lane Background	Lane background subtracted with disk size: 10
Lane Width	7.27 mm
