# Supplementary material for: Multimodal Imaging Techniques to Evaluate the Anticancer Effect of Cold Atmospheric Pressure Plasma
Source: Cancers (Basel). 2021 May 19;13(10):2483. doi: 10.3390/cancers13102483 (PMC8161248; doi:10.3390/cancers13102483)

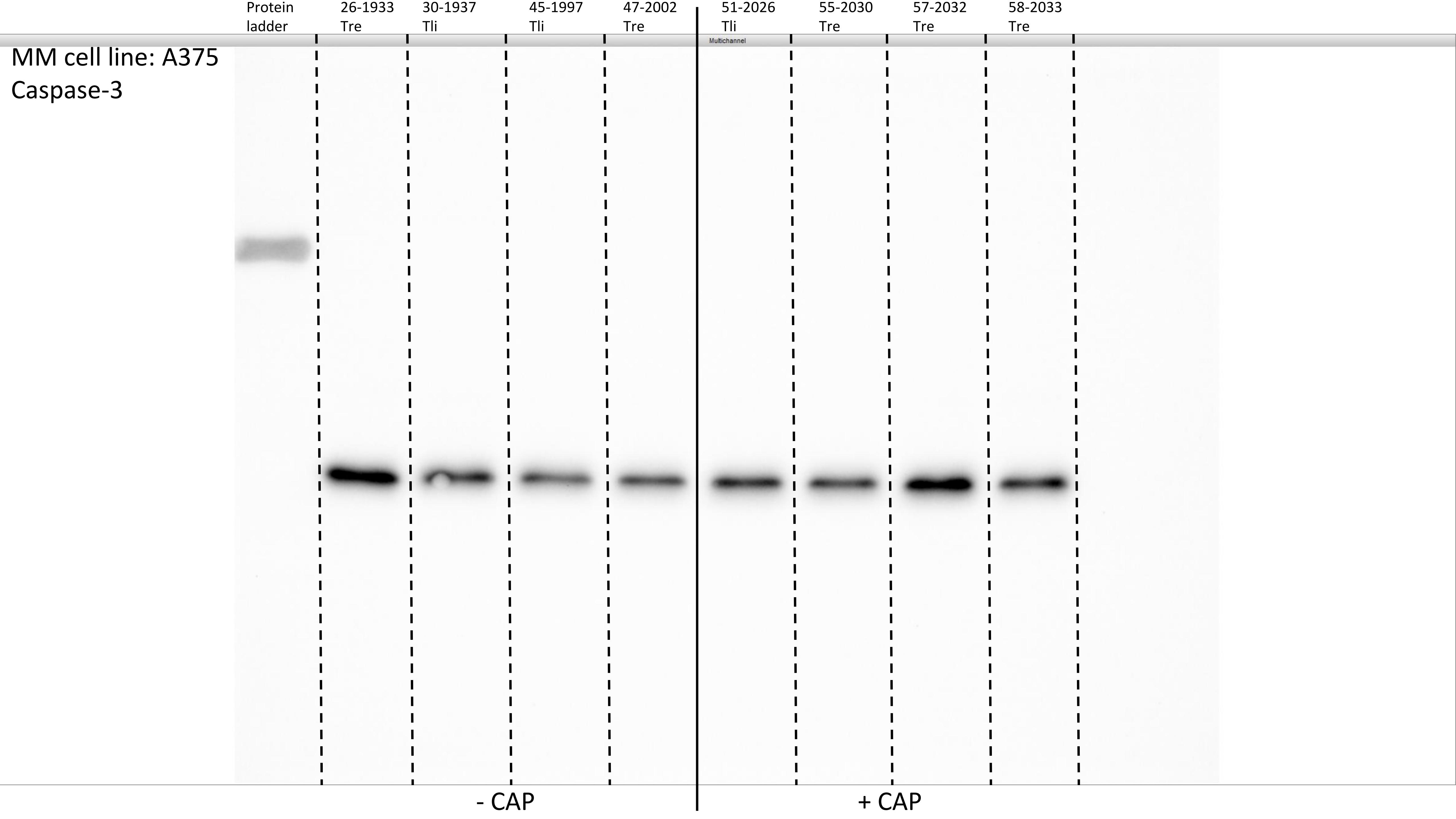

MM cell line: A375
Caspase-3
Protein ladder
26-1933 Tre
30-1937 Tli
45-1997 Tli
47-2002 Tre
51-2026 Tli
55-2030 Tre
57-2032 Tre
58-2033 Tre
Multichannel
- CAP
+ CAP

MM cell line: A375

ß-actin to caspase-3

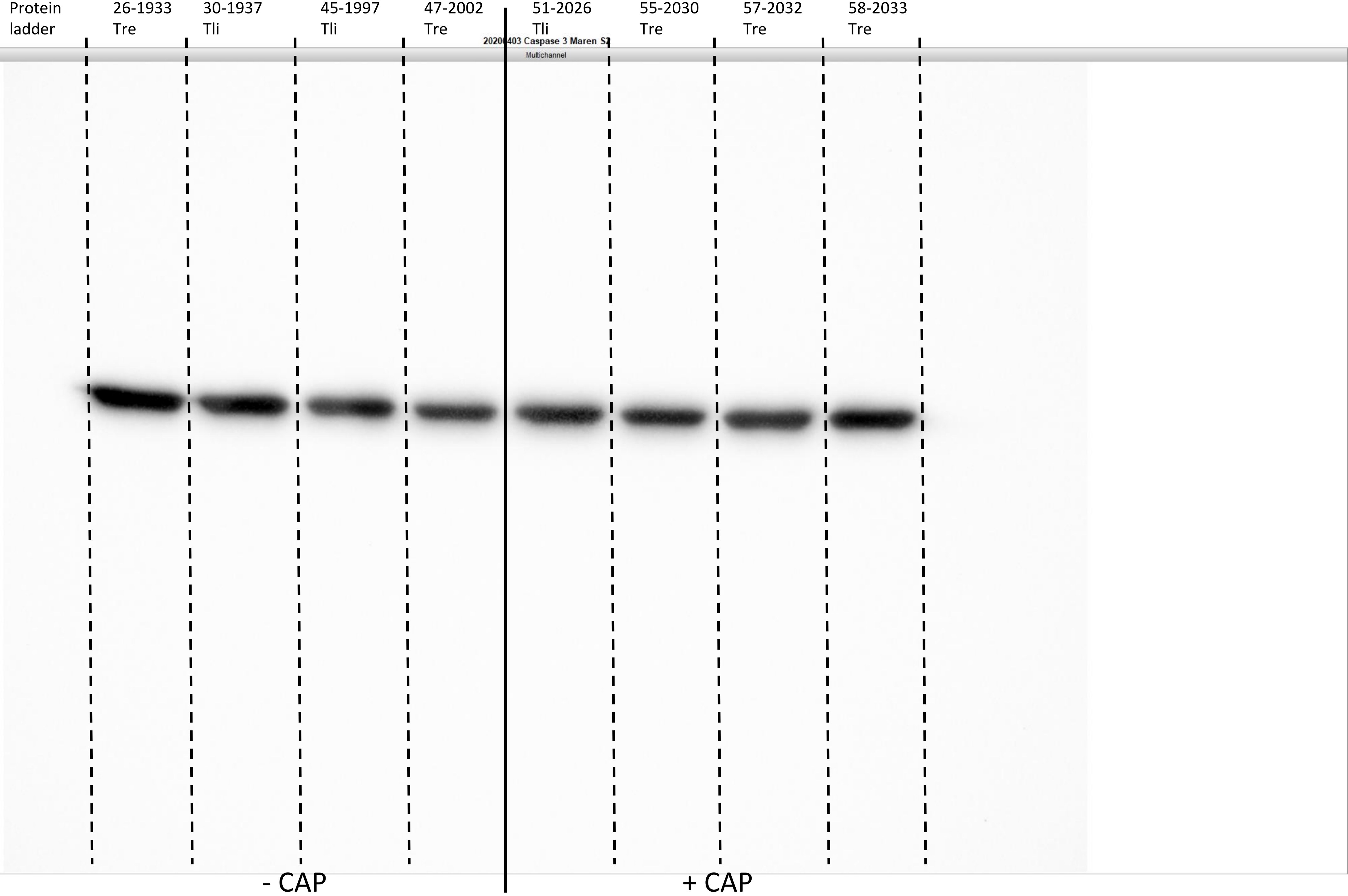

MM cell line: A375
Marker peq Gold IV to
ß-actin and caspase-3

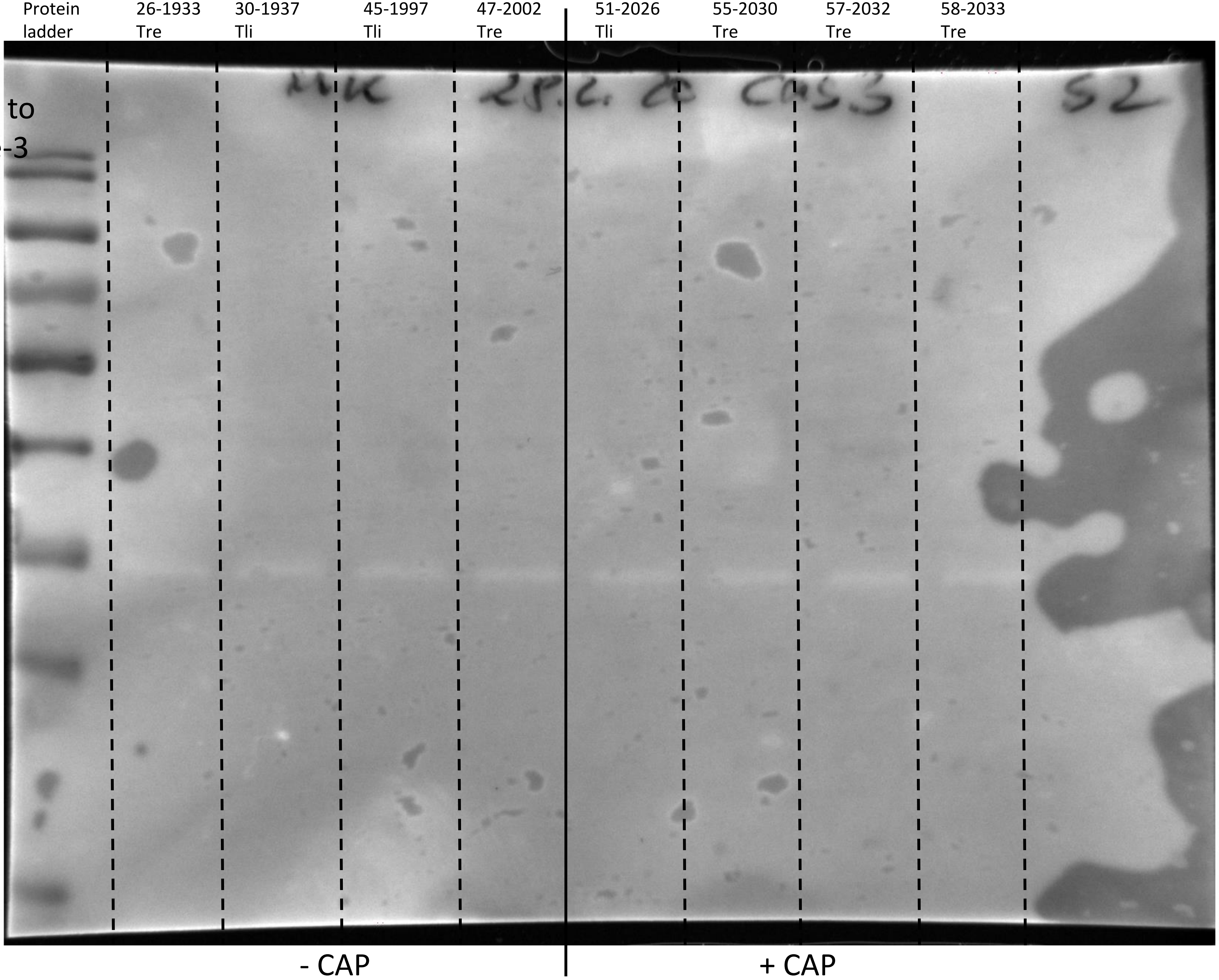

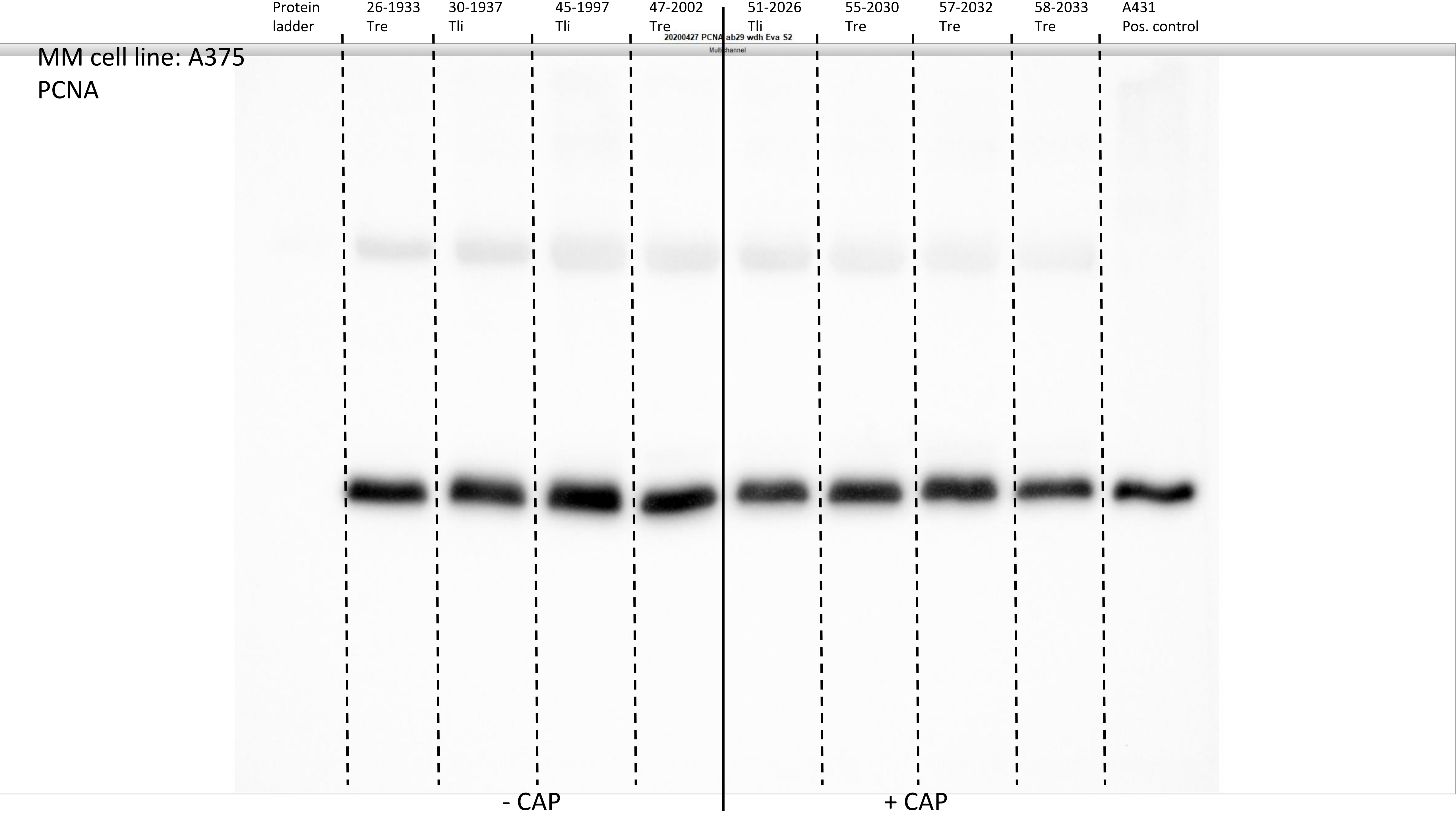
MM cell line: A375
PCNA
Protein ladder
26-1933 Tre
30-1937 Tli
45-1997 Tli
47-2002 Tre
51-2026 Tli
55-2030 Tre
57-2032 Tre
58-2033 Tre
A431 Pos. control
20200427 PCNA ab29 wdh Eva S2
Multichannel
- CAP
+ CAP

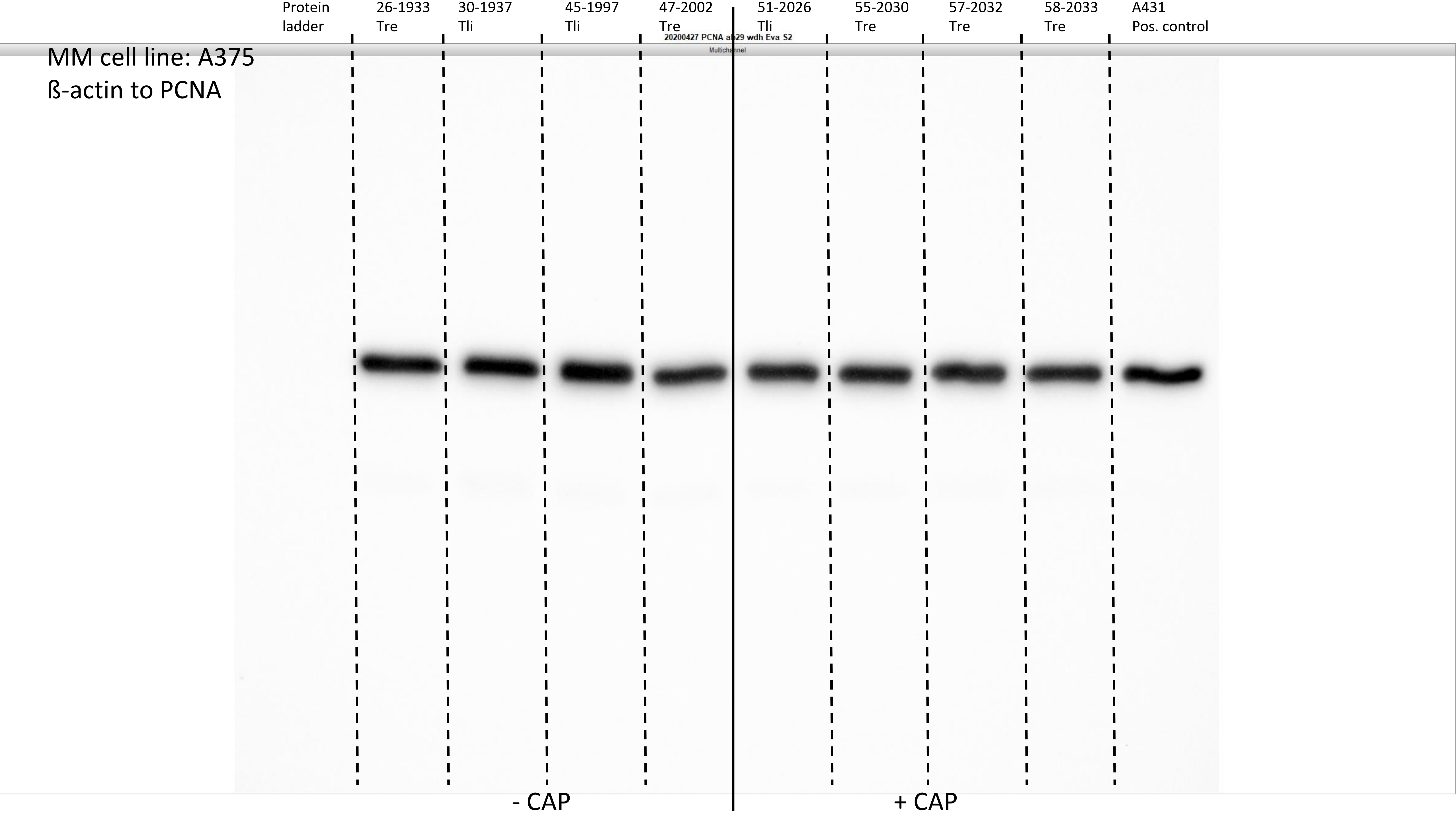
MM cell line: A375
ß-actin to PCNA
Protein ladder
26-1933 Tre
30-1937 Tli
45-1997 Tli
47-2002 Tre
51-2026 Tli
55-2030 Tre
57-2032 Tre
58-2033 Tre
A431 Pos. control
20200427 PCNA ab29 wdh Eva S2
Multichannel
- CAP
+ CAP

MM cell line: A375
Marker peq Gold IV to ß-actin and PCNA

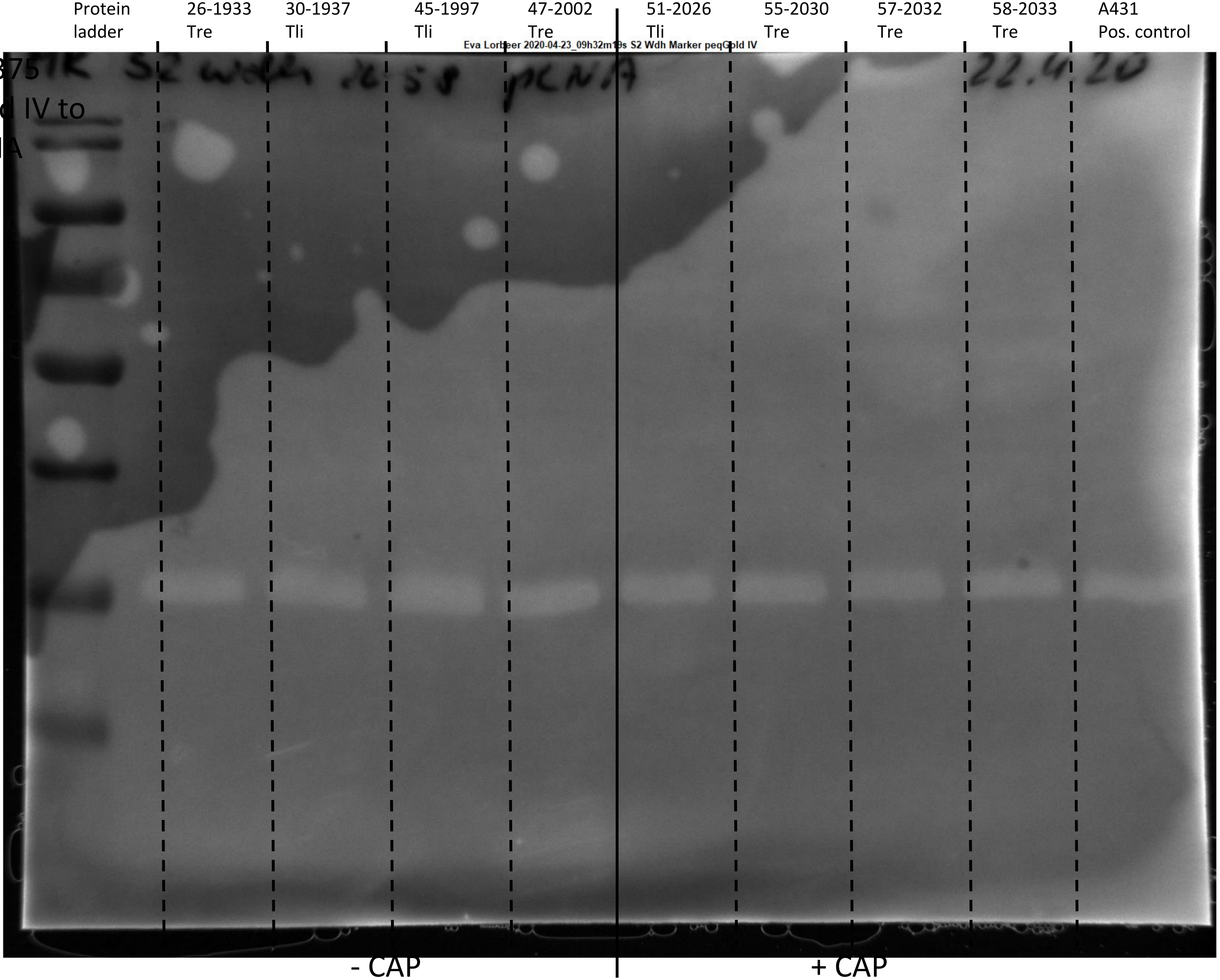

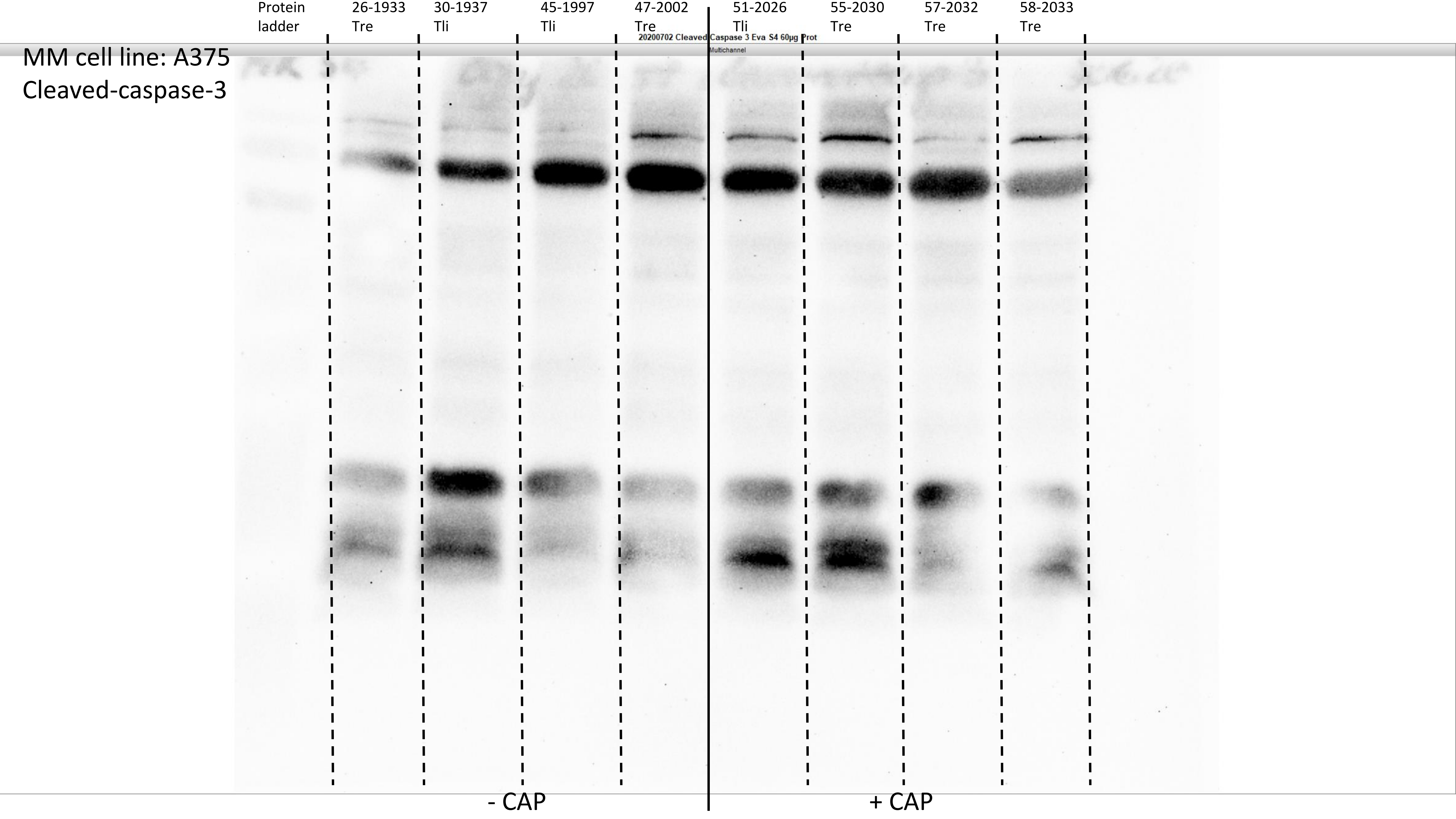
MM cell line: A375
Cleaved-caspase-3
Protein ladder
26-1933 Tre
30-1937 Tli
45-1997 Tli
47-2002 Tre
51-2026 Tli
55-2030 Tre
57-2032 Tre
58-2033 Tre
20200702 Cleaved Caspase 3 Eva S4 60µg Prot
Multichannel
- CAP
+ CAP

MM cell line: A375
ß-actin to
Cleaved-caspase-3

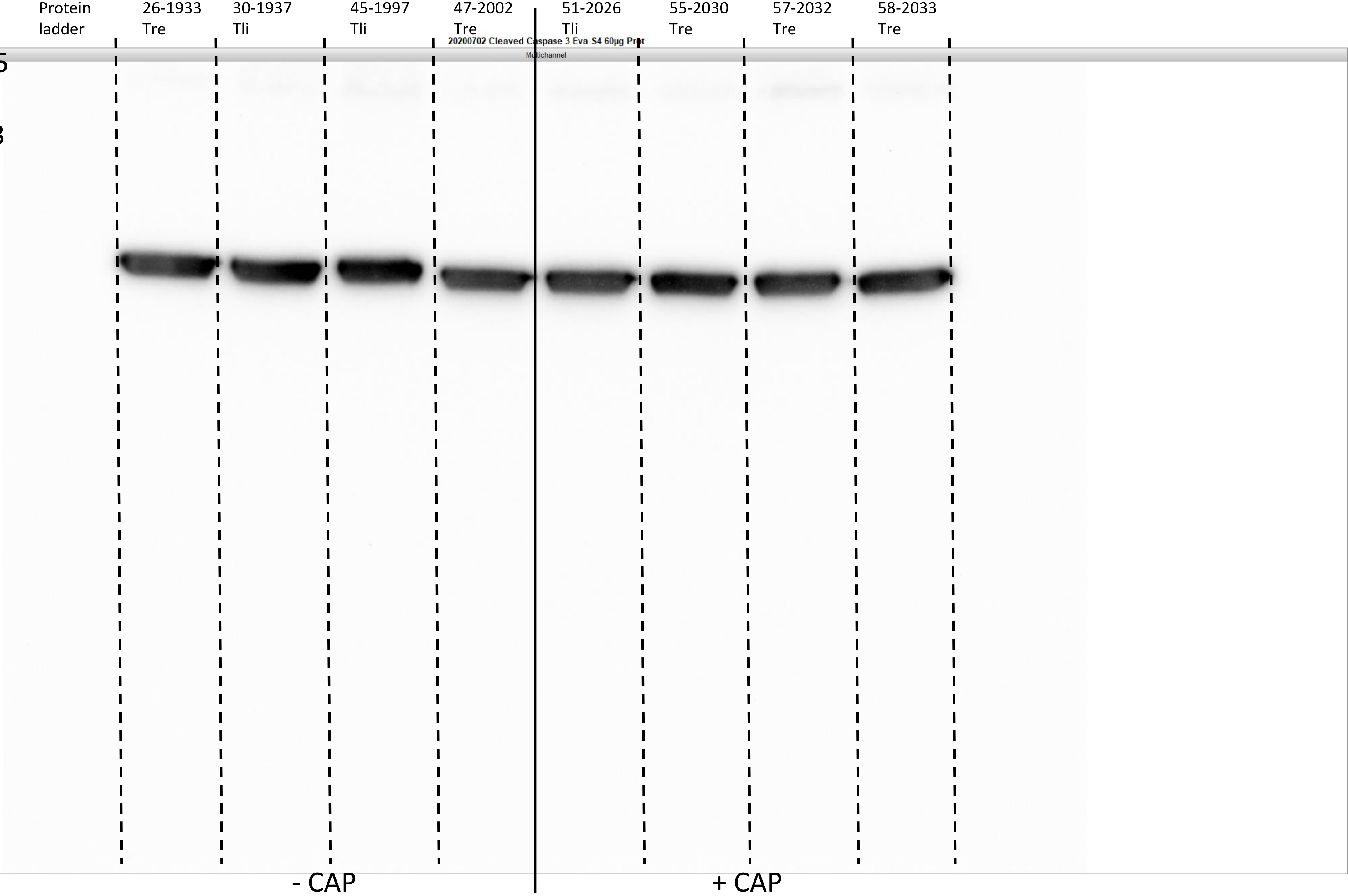

MM cell line: A375
Marker peq Gold IV to
ß-actin and
Cleaved-caspase-3

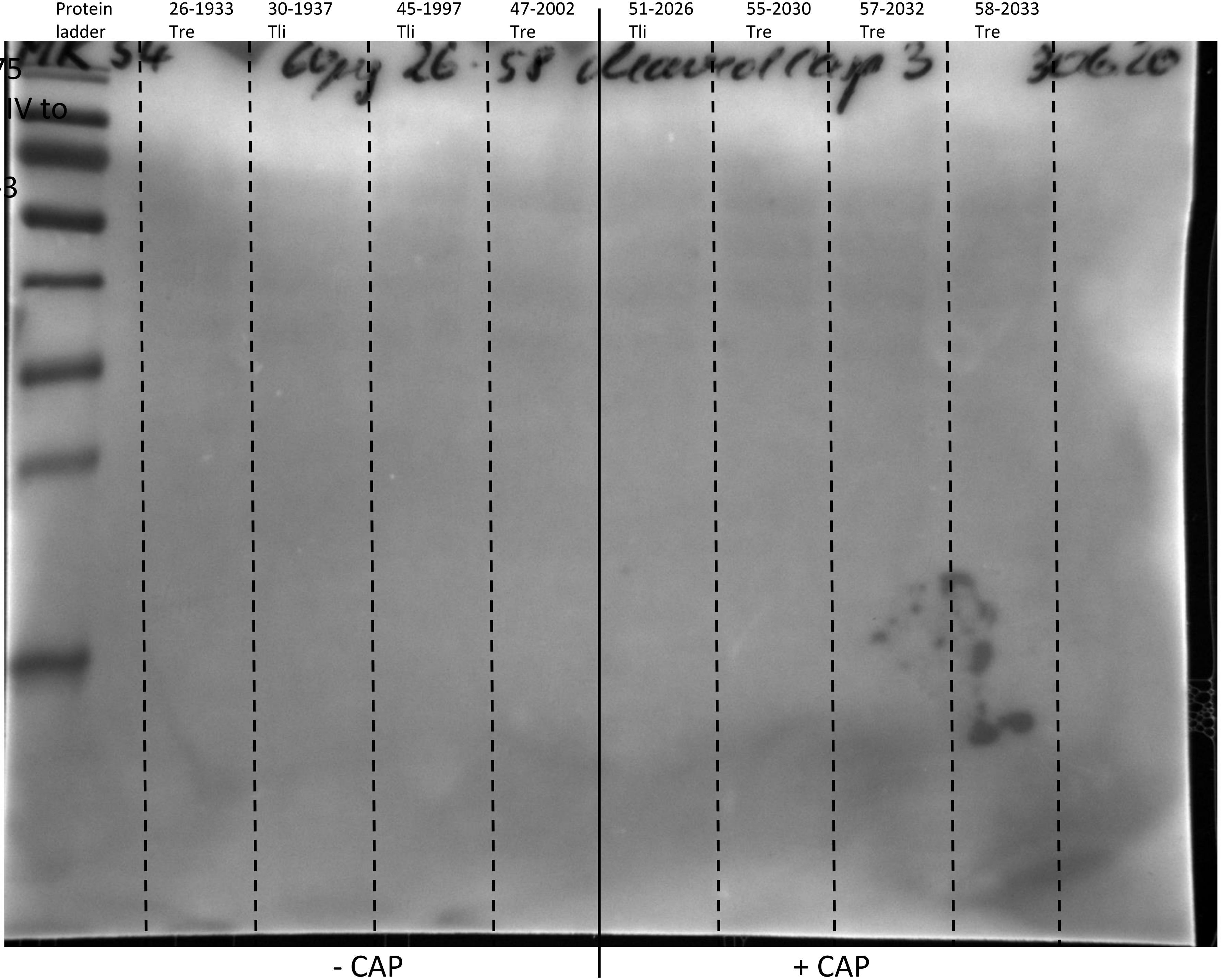

SCC cell line: A431
Caspase-3

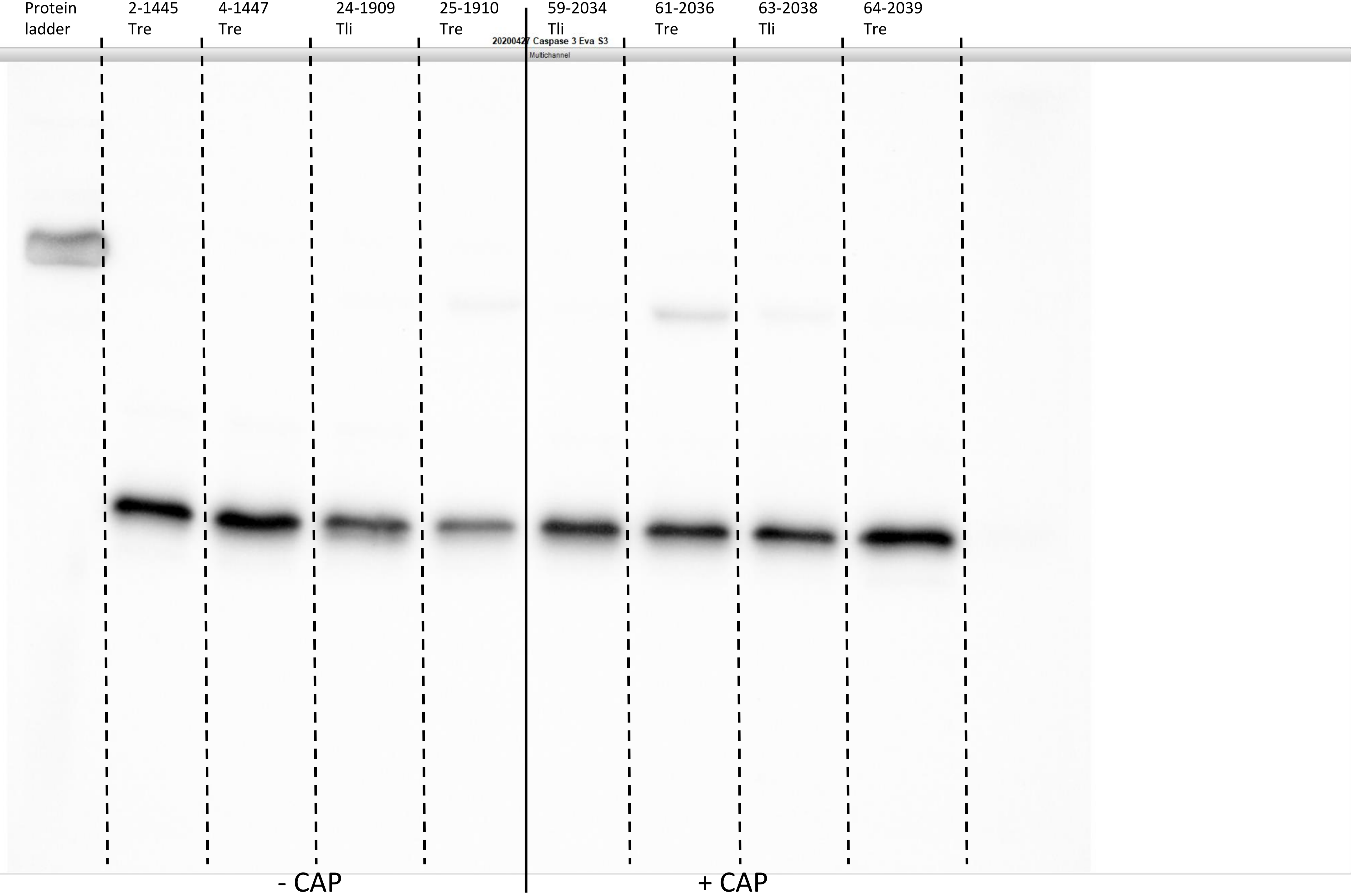

SCC cell line: A431
ß-actin to caspase-3

Protein ladder | 2-1445 Tre | 4-1447 Tre | 24-1909 Tli | 25-1910 Tre | 59-2034 Tli | 61-2036 Tre | 63-2038 Tli | 64-2039 Tre

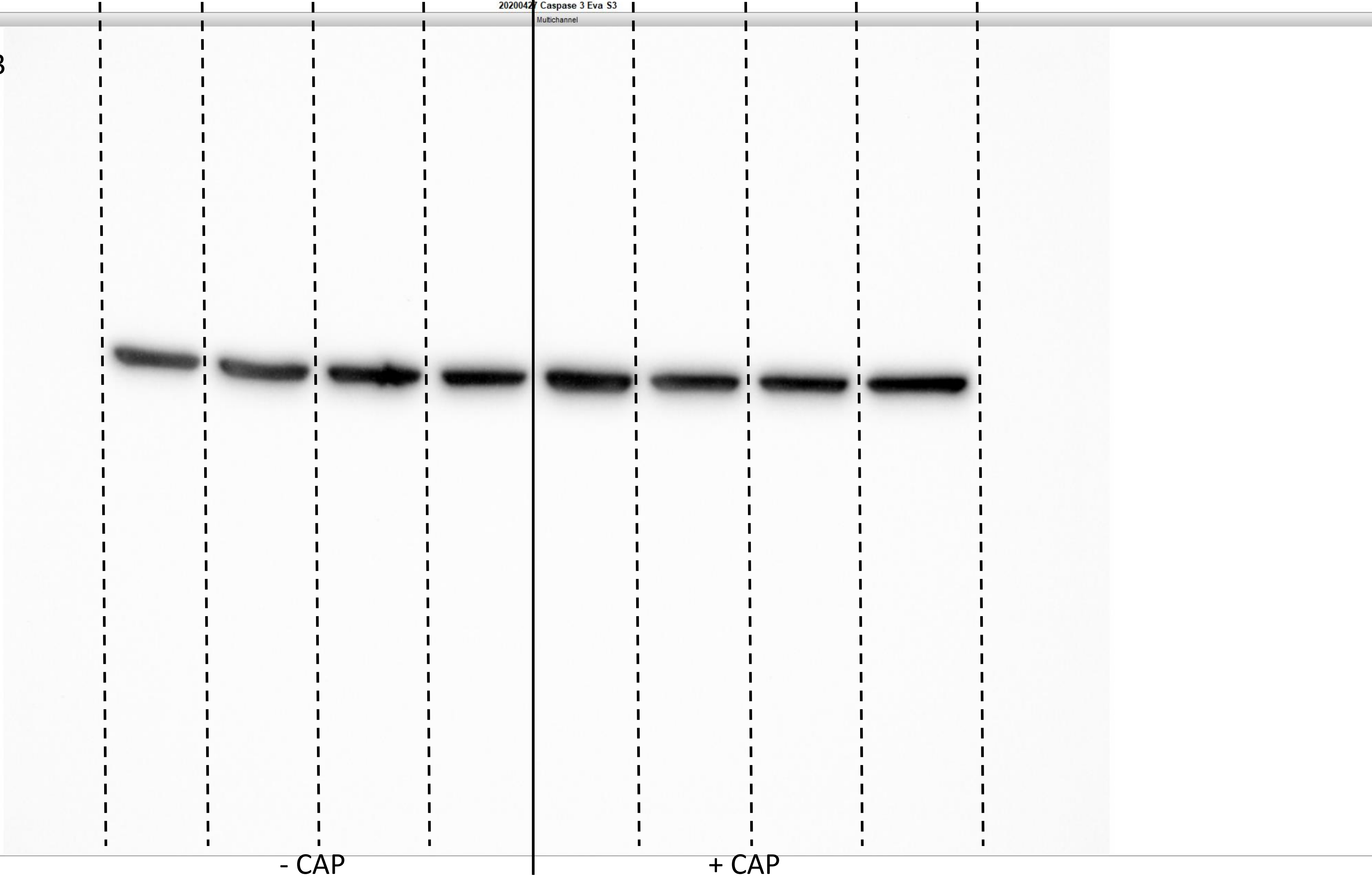

- CAP

+ CAP

SCC cell line: A431
Marker peq Gold IV to
ß-actin and caspase-3

Protein ladder | 2-1445 Tre | 4-1447 Tre | 24-1909 Tli | 25-1910 Tre | 59-2034 Tli | 61-2036 Tre | 63-2038 Tli | 64-2039 Tre

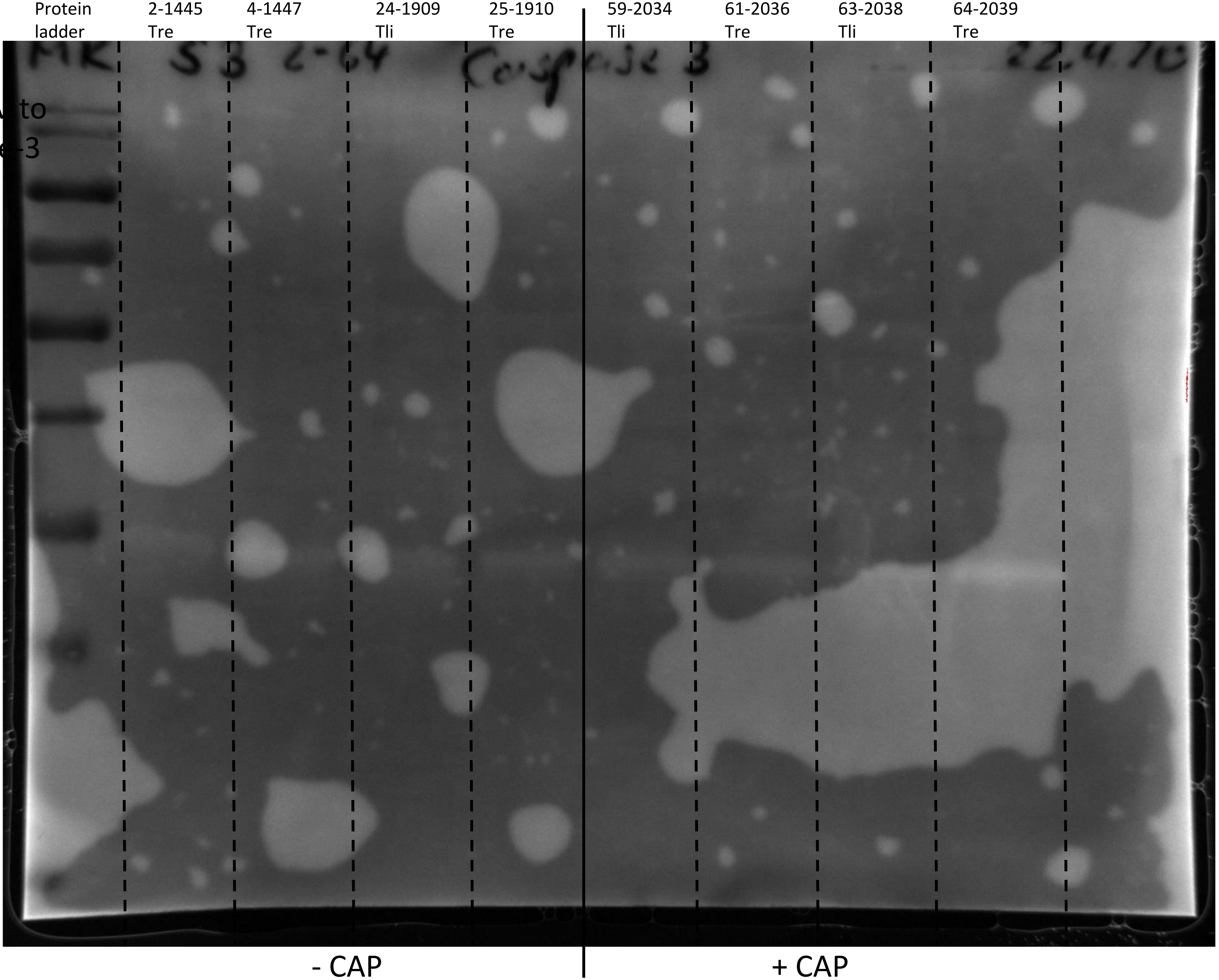

- CAP | + CAP

SCC cell line: A431
PCNA

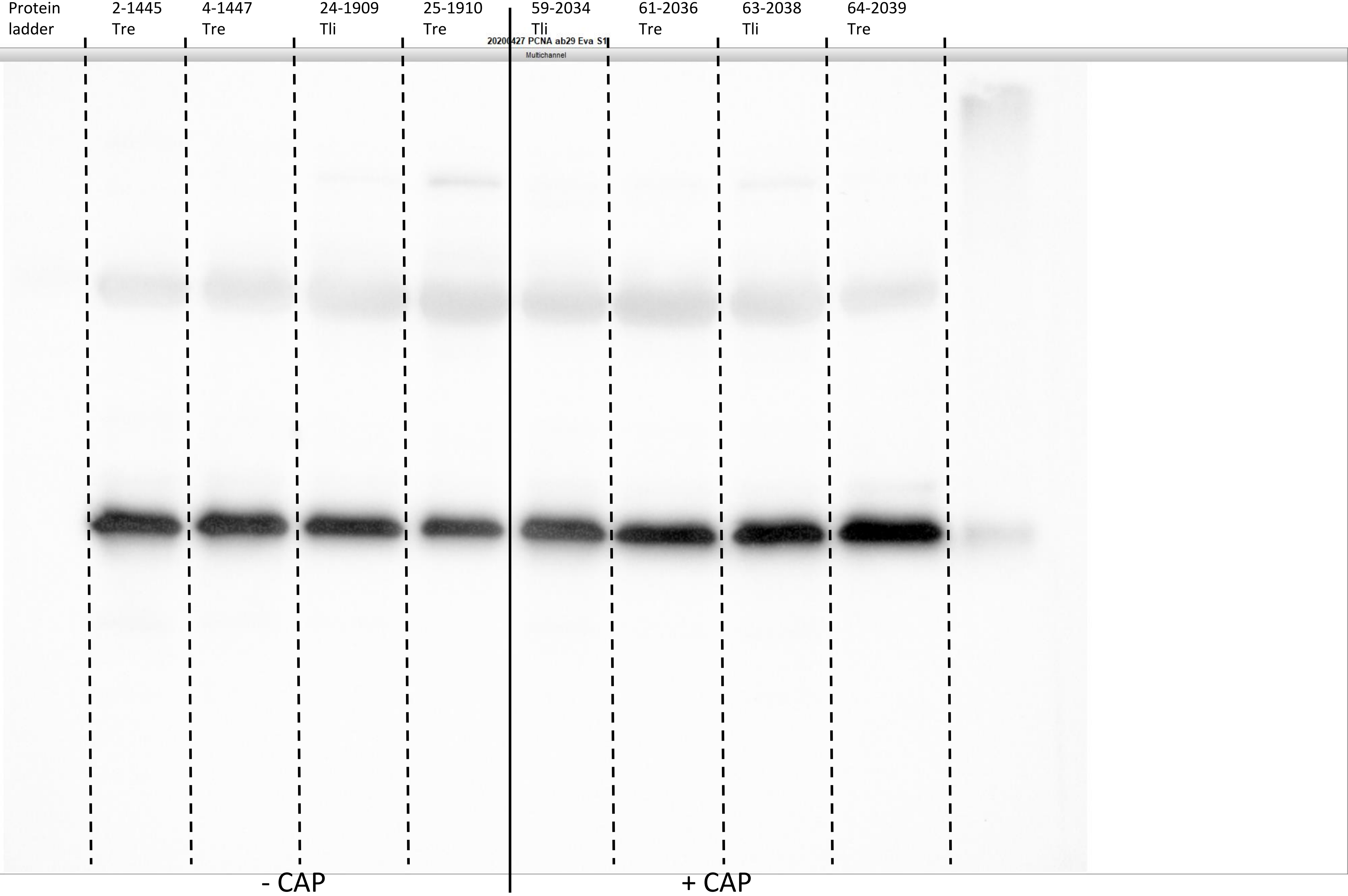

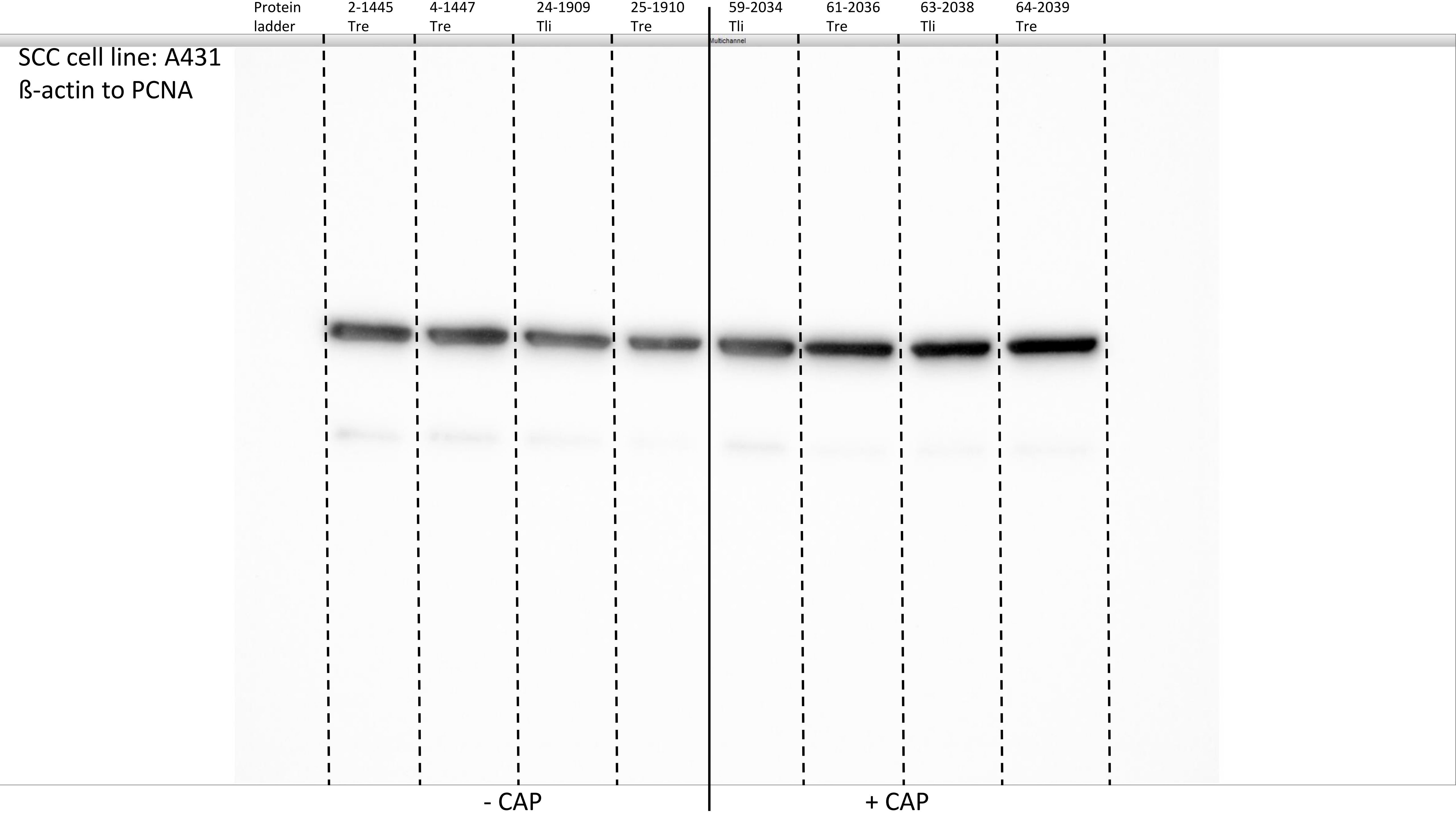
SCC cell line: A431
ß-actin to PCNA
Protein ladder
2-1445 Tre
4-1447 Tre
24-1909 Tli
25-1910 Tre
59-2034 Tli
61-2036 Tre
63-2038 Tli
64-2039 Tre
Multichannel
- CAP
+ CAP

SCC cell line: A431
Marker peq Gold IV to
ß-actin and PCNA

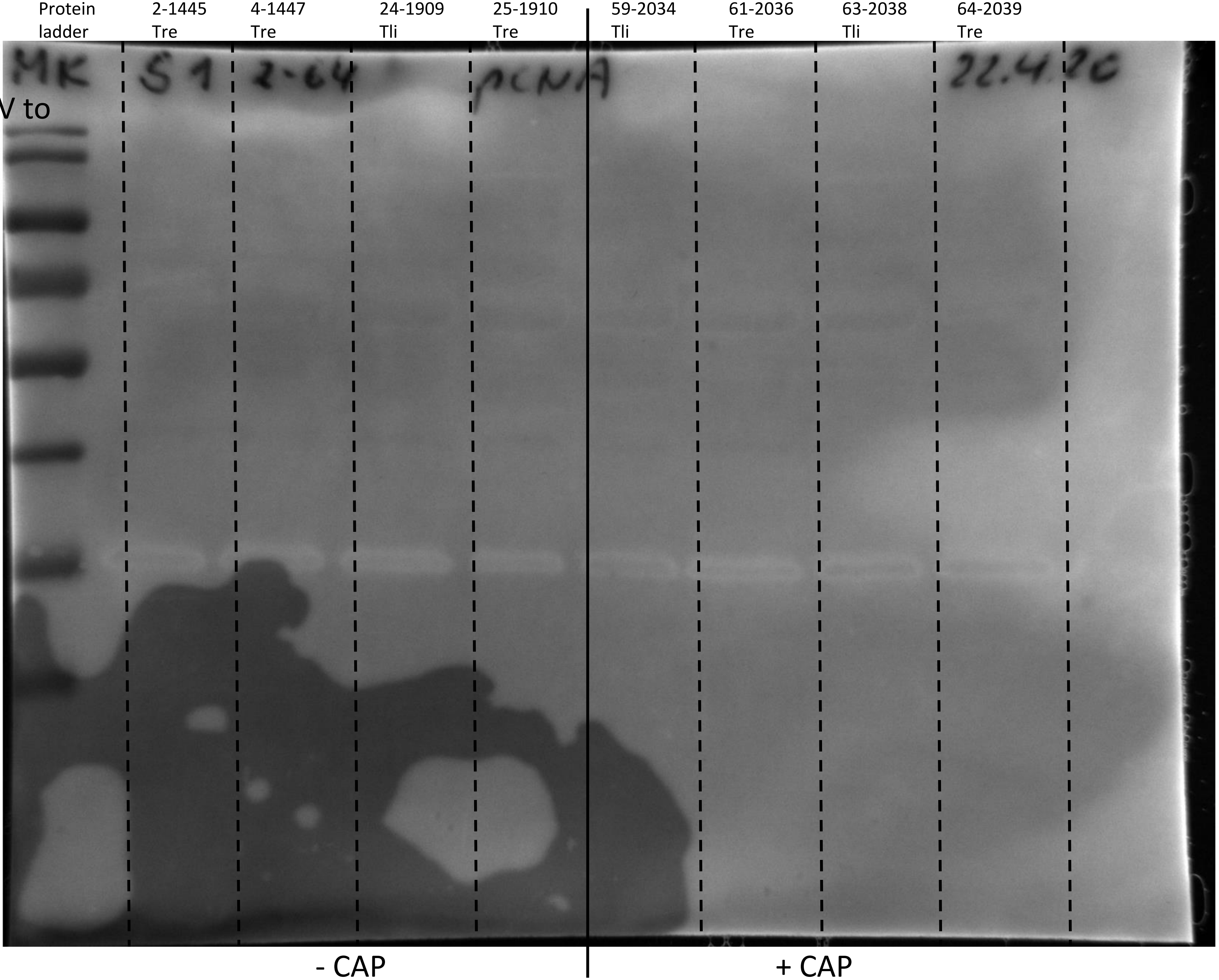

SCC cell line: A431
Cleaved-caspase-3

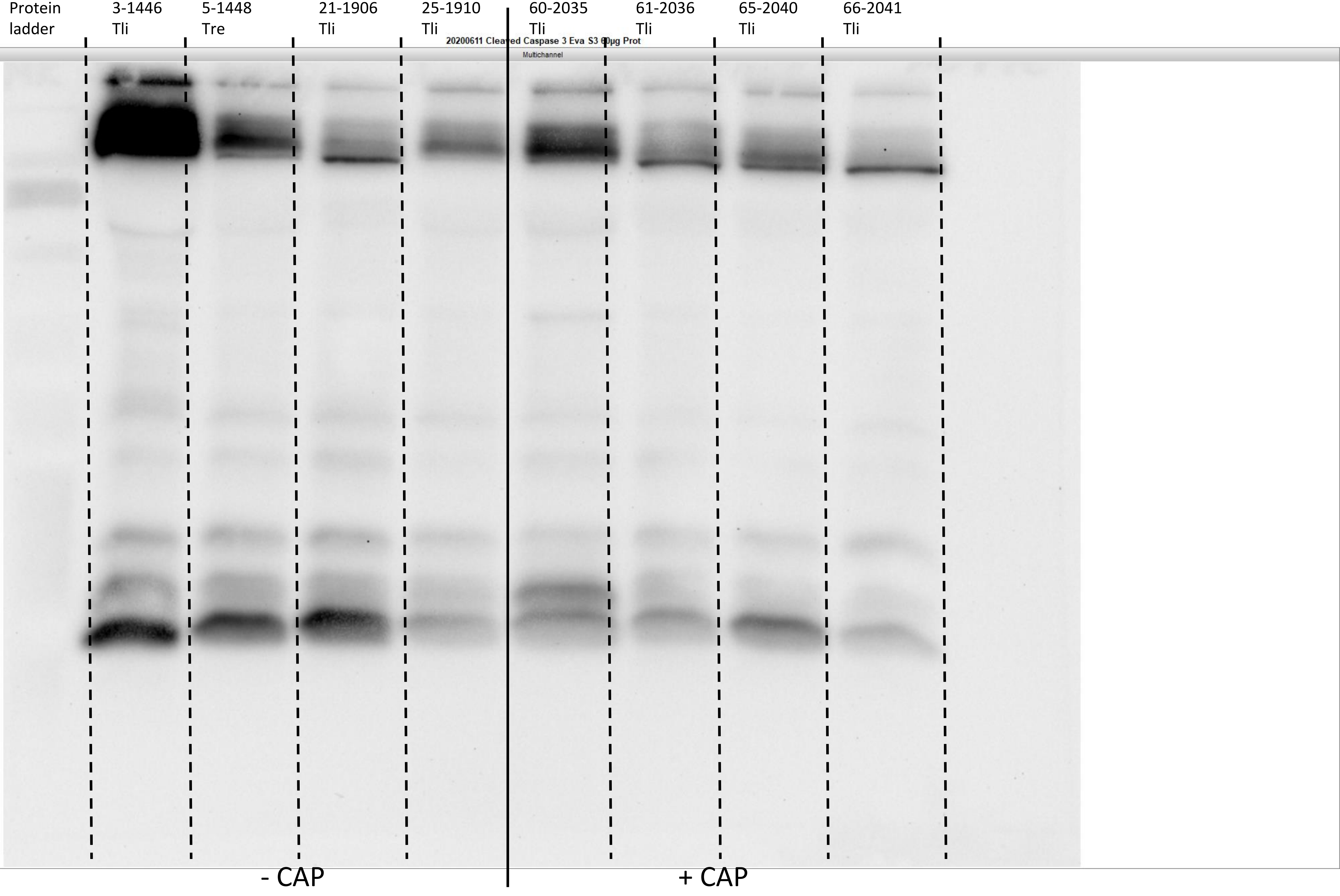

SCC cell line: A431
ß-actin to
Cleaved-caspase-3

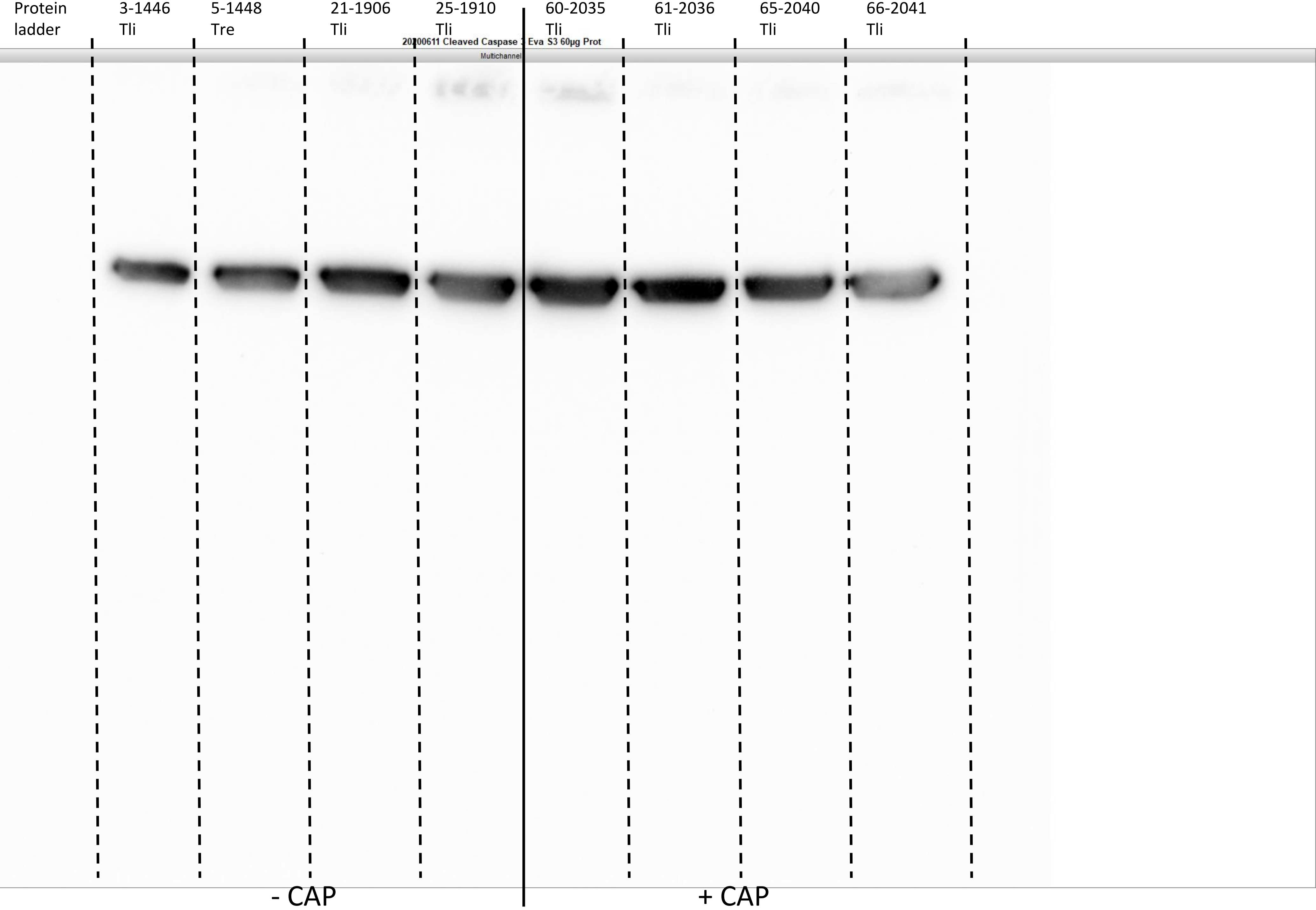

SCC cell line: A431
Marker peq Gold IV to
ß-actin and
Cleaved-caspase-3

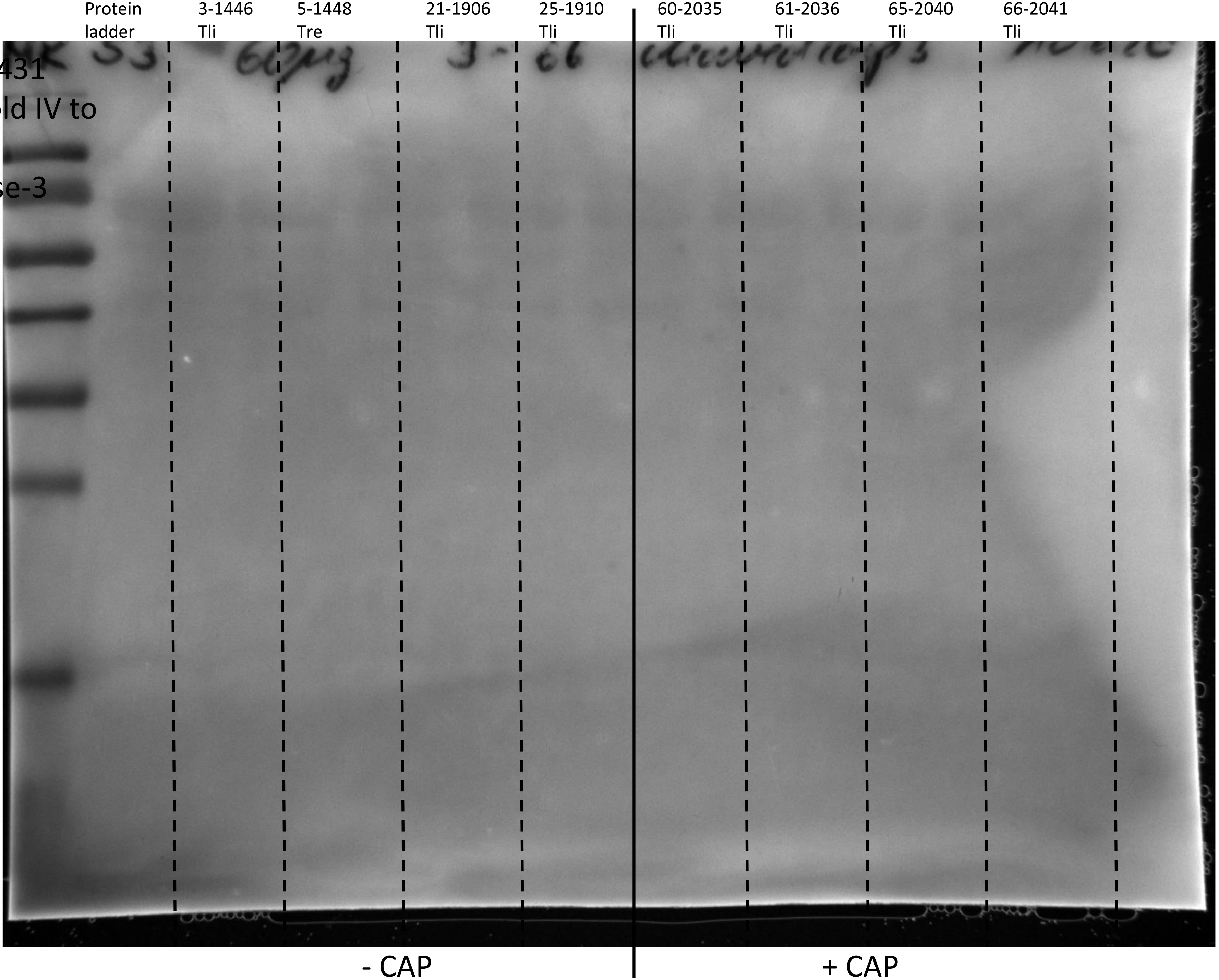

Supplement: Supplementary file 1 [file cancers-13-02483-s001.zip › cancers-1174396-supplementary/Figure S2. Original Western Blot images/original gel images figure 5.pdf]
