# Supplementary material for: Multimodal Imaging Techniques to Evaluate the Anticancer Effect of Cold Atmospheric Pressure Plasma
Source: Cancers (Basel). 2021 May 19;13(10):2483. doi: 10.3390/cancers13102483 (PMC8161248; doi:10.3390/cancers13102483)
Supplement: Supplementary file 1 [file cancers-13-02483-s001.zip › cancers-1174396-supplementary/Figure S2. Original Western Blot images/SCC cell line A431 caspase-3 ß-actin.pdf]

Image Report: 20200427 Caspase 3 Eva S3

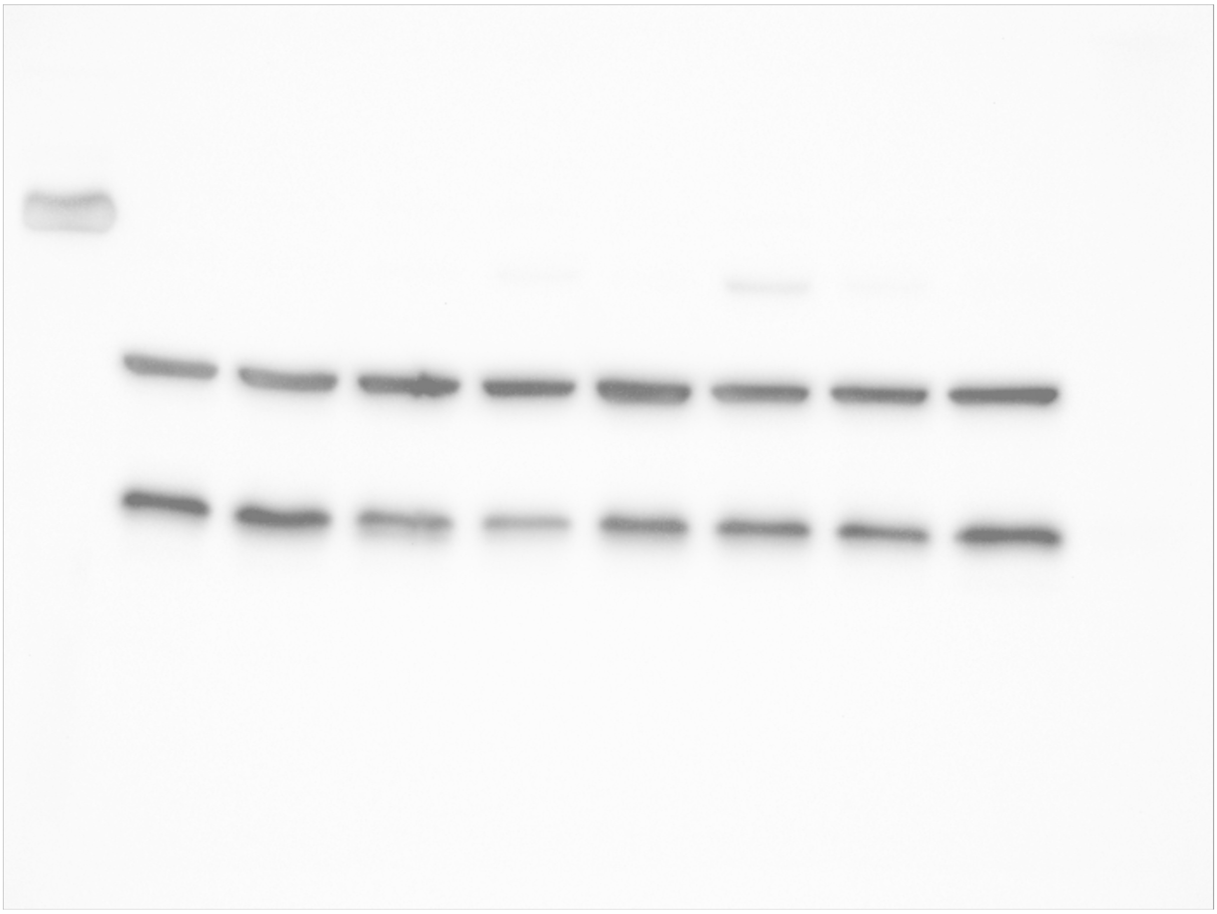

C:\Users\marcel.kordt\Desktop\Marcel\Versuche\Probenaufbereitung\Mol\WB März 2020 mit und ohne KAP\Auswertung Finall\20200427 Caspase 3 Eva S3.mscn

---

Channel 1 - Red - Chemi Hi Resolution

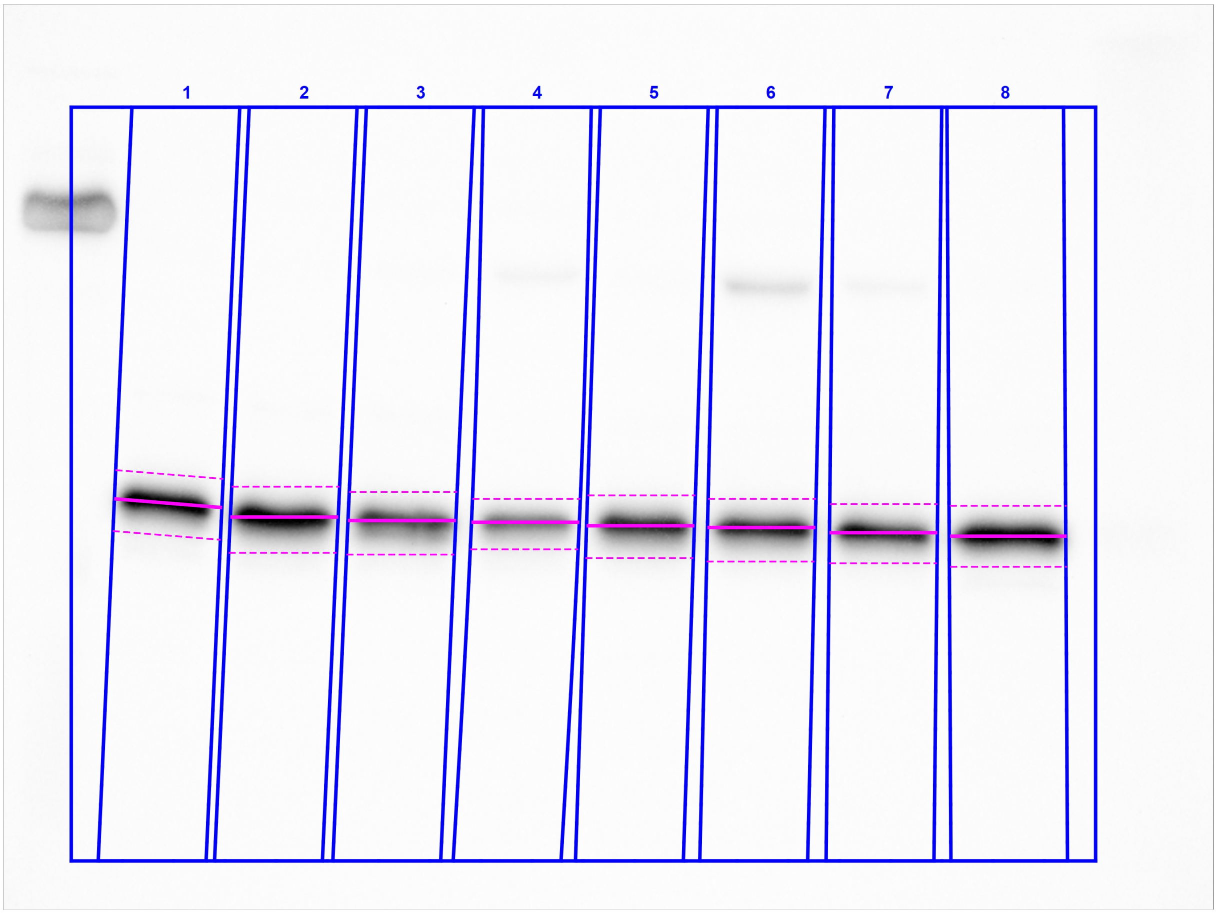

Lane Statistics

Channel	Lane No.	Adj. Total Band Vol. (Int)	Total Band Vol. (Int)	Adj. Total Lane Vol. (Int)	Total Lane Vol. (Int)	Bkgd. Vol. (Int)	Norm. Factor
Chemi Hi Resolution	1	39.511.918	42.260.130	42.804.490	75.029.300	32.224.810	N/A
Chemi Hi Resolution	2	45.428.578	48.351.506	48.098.794	79.229.180	31.130.386	N/A
Chemi Hi Resolution	3	32.988.464	35.613.420	35.598.540	65.463.754	29.865.214	N/A
Chemi Hi Resolution	4	20.073.740	22.001.754	23.637.748	52.270.340	28.632.592	N/A
Chemi Hi Resolution	5	34.733.764	37.317.738	37.180.036	66.544.538	29.364.502	N/A
Chemi Hi Resolution	6	35.280.728	37.898.058	40.981.132	70.975.802	29.994.670	N/A
Chemi Hi Resolution	7	32.584.782	35.166.276	35.794.460	66.855.902	31.061.442	N/A
Chemi Hi Resolution	8	45.288.851	48.166.903	48.706.588	83.504.177	34.797.589	N/A

Lane And Band Analysis

Lane 1

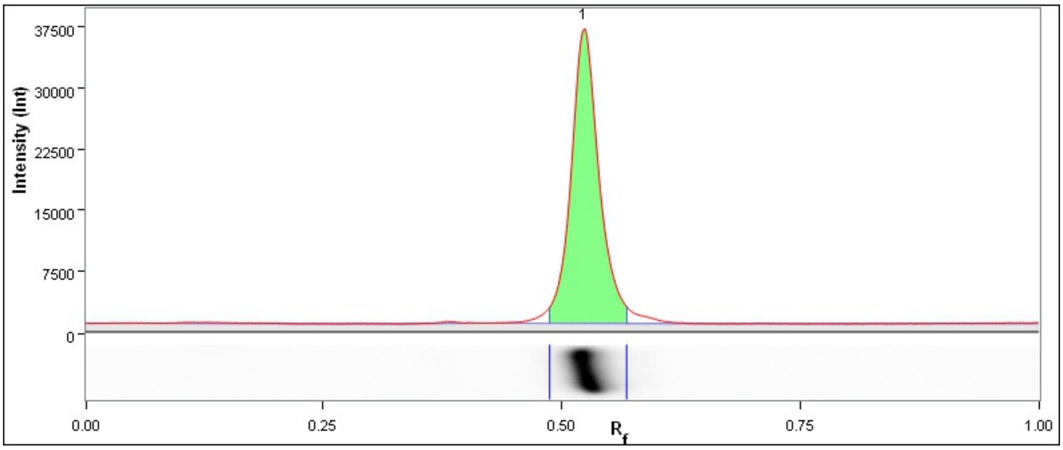

Channel	Band No.	Band Label	Mol. Wt. (KDa)	Relative Front	Adj. Volume (Int)	Volume (Int)	Abs. Quant.	Rel. Quant.	Band %	Lane %	Norm. Factor	Norm. Vol. (Int)
Chemi Hi Resolution	1		N/A	0,525	39.511.918	42.260.130	N/A	N/A	100,0	92,3	N/A	N/A

Band Detection	Automatically detected bands with sensitivity: Low
Lane Background	Lane background subtracted with disk size: 10
Lane Width	7.39 mm

Lane 2

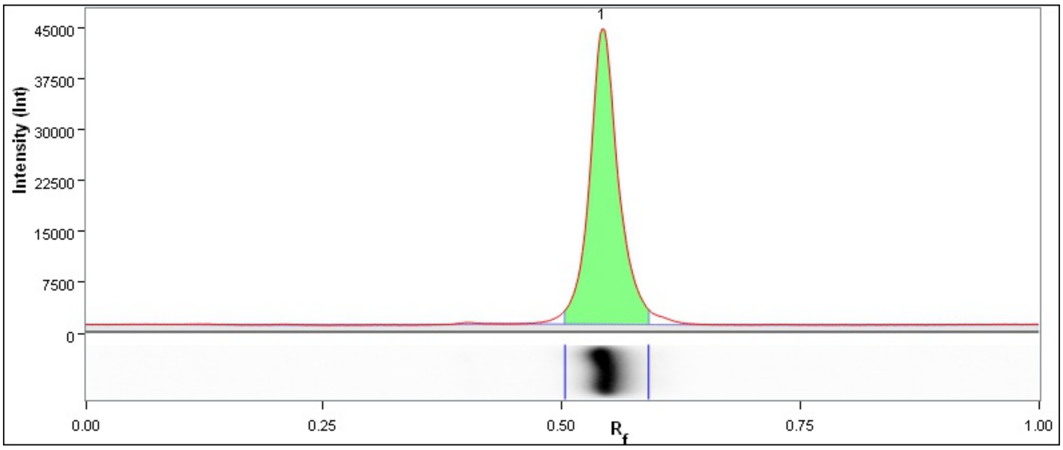

Channel	Band No.	Band Label	Mol. Wt. (KDa)	Relative Front	Adj. Volume (Int)	Volume (Int)	Abs. Quant.	Rel. Quant.	Band %	Lane %	Norm. Factor	Norm. Vol. (Int)
Chemi Hi Resolution	1		N/A	0,544	45.428.578	48.351.506	N/A	N/A	100,0	94,4	N/A	N/A

Band Detection	Automatically detected bands with sensitivity: Low
Lane Background	Lane background subtracted with disk size: 10
Lane Width	7.39 mm

Lane 3

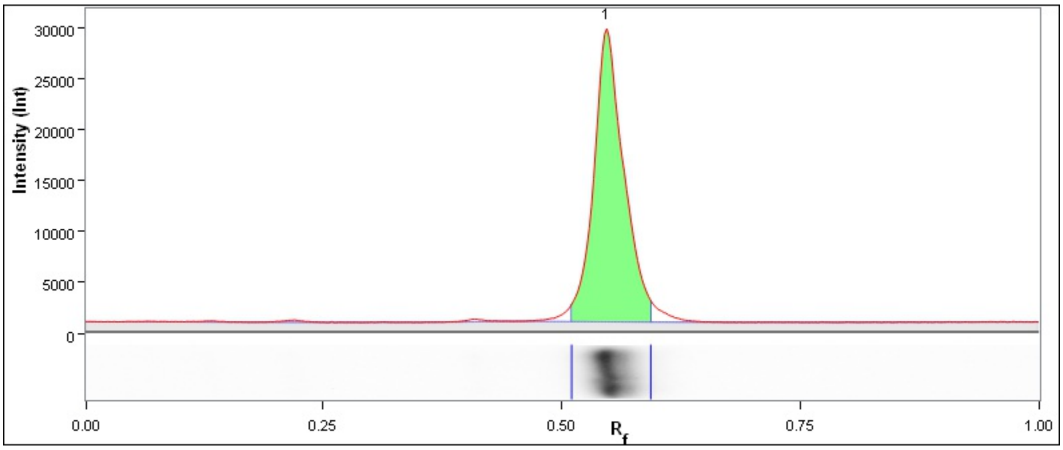

Channel	Band No.	Band Label	Mol. Wt. (KDa)	Relative Front	Adj. Volume (Int)	Volume (Int)	Abs. Quant.	Rel. Quant.	Band %	Lane %	Norm. Factor	Norm. Vol. (Int)
Chemi Hi Resolution	1		N/A	0,548	32.988.464	35.613.420	N/A	N/A	100,0	92,7	N/A	N/A

Band Detection	Automatically detected bands with sensitivity: Low
Lane Background	Lane background subtracted with disk size: 10
Lane Width	7.39 mm

Lane 4

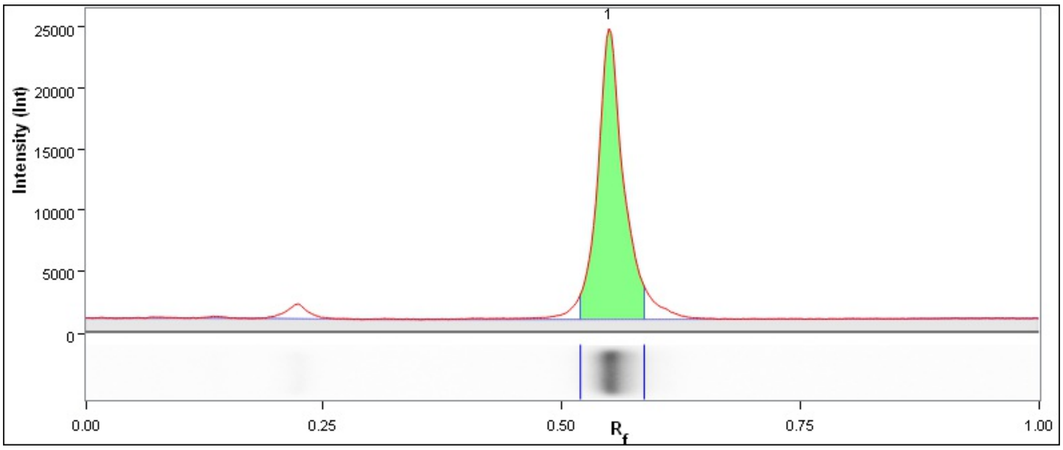

Channel	Band No.	Band Label	Mol. Wt. (KDa)	Relative Front	Adj. Volume (Int)	Volume (Int)	Abs. Quant.	Rel. Quant.	Band %	Lane %	Norm. Factor	Norm. Vol. (Int)
Chemi Hi Resolution	1		N/A	0,551	20.073.740	22.001.754	N/A	N/A	100,0	84,9	N/A	N/A

Band Detection	Automatically detected bands with sensitivity: Low
Lane Background	Lane background subtracted with disk size: 10
Lane Width	7.39 mm

Lane 5

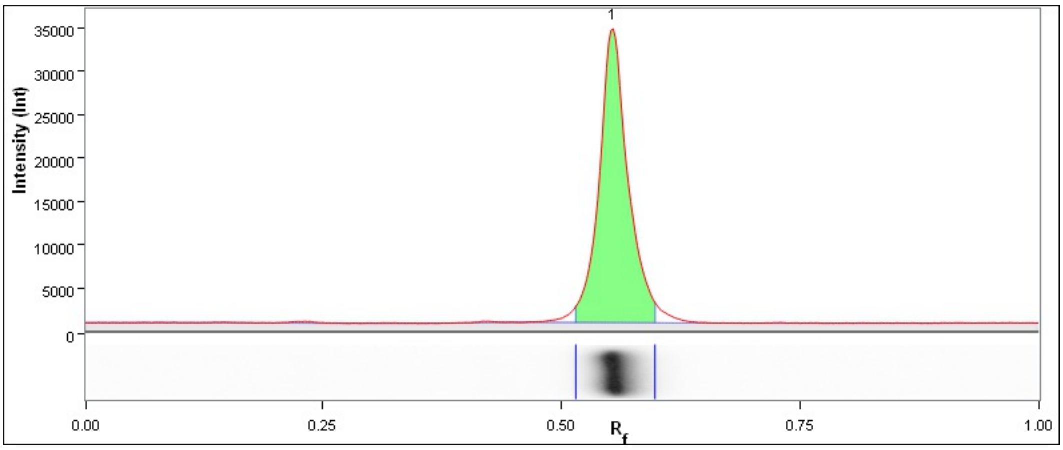

Channel	Band No.	Band Label	Mol. Wt. (KDa)	Relative Front	Adj. Volume (Int)	Volume (Int)	Abs. Quant.	Rel. Quant.	Band %	Lane %	Norm. Factor	Norm. Vol. (Int)
Chemi Hi Resolution	1		N/A	0,555	34.733.764	37.317.738	N/A	N/A	100,0	93,4	N/A	N/A

Band Detection	Automatically detected bands with sensitivity: Low
Lane Background	Lane background subtracted with disk size: 10
Lane Width	7.39 mm

Lane 6

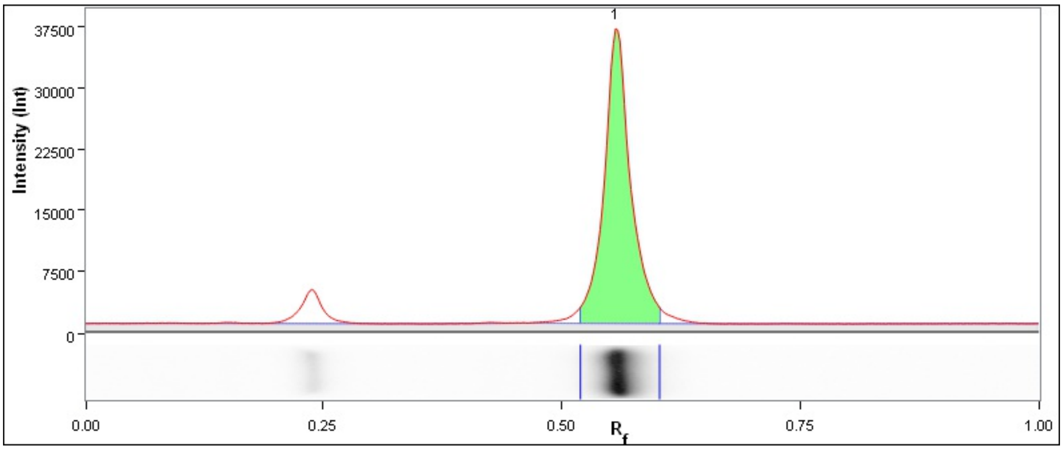

Channel	Band No.	Band Label	Mol. Wt. (KDa)	Relative Front	Adj. Volume (Int)	Volume (Int)	Abs. Quant.	Rel. Quant.	Band %	Lane %	Norm. Factor	Norm. Vol. (Int)
Chemi Hi Resolution	1		N/A	0,558	35.280.728	37.898.058	N/A	N/A	100,0	86,1	N/A	N/A

Band Detection	Automatically detected bands with sensitivity: Low
Lane Background	Lane background subtracted with disk size: 10
Lane Width	7.39 mm

Lane 7

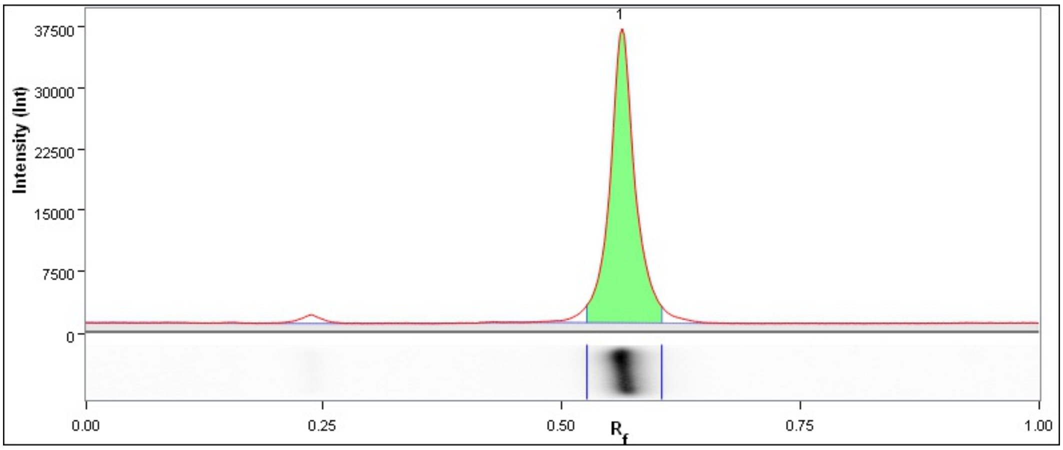

Channel	Band No.	Band Label	Mol. Wt. (KDa)	Relative Front	Adj. Volume (Int)	Volume (Int)	Abs. Quant.	Rel. Quant.	Band %	Lane %	Norm. Factor	Norm. Vol. (Int)
Chemi Hi Resolution	1		N/A	0,565	32.584.782	35.166.276	N/A	N/A	100,0	91,0	N/A	N/A

Band Detection	Automatically detected bands with sensitivity: Low
Lane Background	Lane background subtracted with disk size: 10
Lane Width	7.39 mm

Lane 8

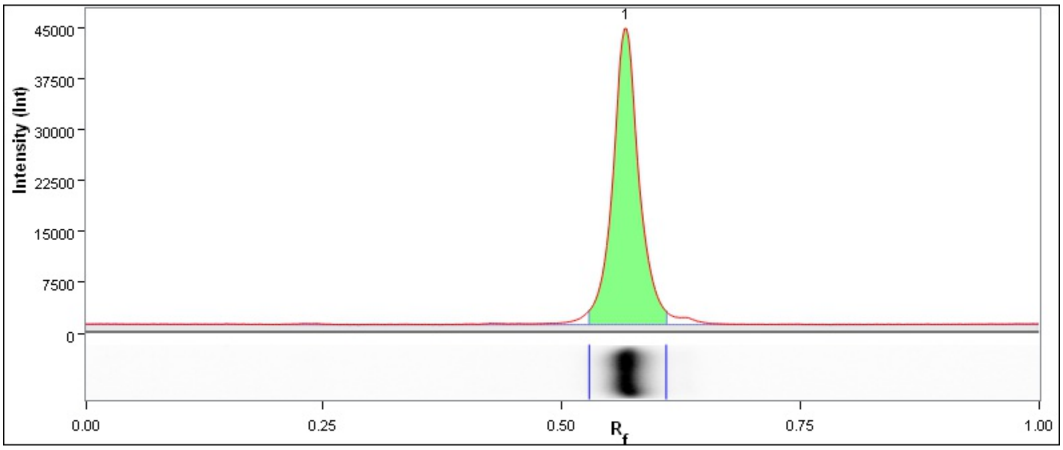

Channel	Band No.	Band Label	Mol. Wt. (KDa)	Relative Front	Adj. Volume (Int)	Volume (Int)	Abs. Quant.	Rel. Quant.	Band %	Lane %	Norm. Factor	Norm. Vol. (Int)
Chemi Hi Resolution	1		N/A	0,569	45.288.851	48.166.903	N/A	N/A	100,0	93,0	N/A	N/A

Band Detection	Automatically detected bands with sensitivity: Low
Lane Background	Lane background subtracted with disk size: 10
Lane Width	7.99 mm

Channel 2 - Green - Chemi Hi Resolution

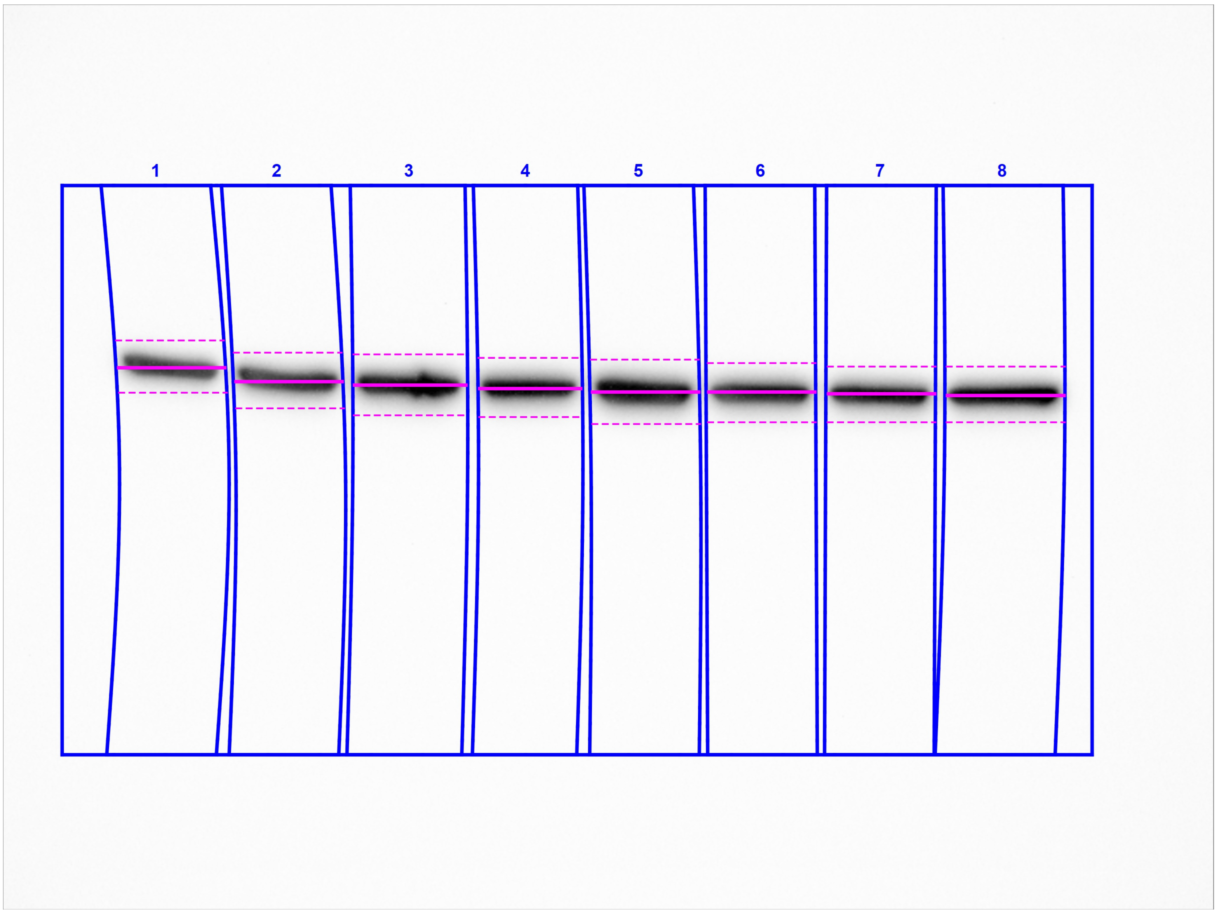

Lane Statistics

Channel	Lane No.	Adj. Total Band Vol. (Int)	Total Band Vol. (Int)	Adj. Total Lane Vol. (Int)	Total Lane Vol. (Int)	Bkgd. Vol. (Int)	Norm. Factor
Chemi Hi Resolution	1	28.178.325	30.696.498	29.683.521	56.123.361	26.439.840	N/A
Chemi Hi Resolution	2	33.162.759	35.700.651	34.891.416	60.047.505	25.156.089	N/A
Chemi Hi Resolution	3	43.945.572	46.747.074	45.878.184	71.304.420	25.426.236	N/A
Chemi Hi Resolution	4	37.017.120	39.484.140	38.654.580	61.423.740	22.769.160	N/A
Chemi Hi Resolution	5	43.929.963	46.707.318	45.689.868	69.689.529	23.999.661	N/A
Chemi Hi Resolution	6	35.861.805	38.501.694	37.323.468	61.838.910	24.515.442	N/A
Chemi Hi Resolution	7	36.220.086	38.773.413	37.857.771	63.114.534	25.256.763	N/A
Chemi Hi Resolution	8	44.124.810	47.091.258	46.015.479	75.248.088	29.232.609	N/A

Lane And Band Analysis

Lane 1

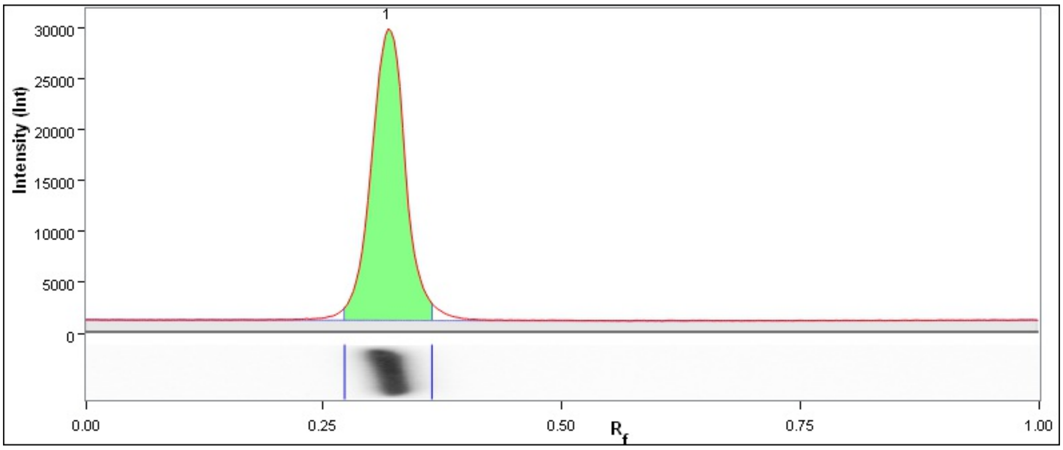

Channel	Band No.	Band Label	Mol. Wt. (KDa)	Relative Front	Adj. Volume (Int)	Volume (Int)	Abs. Quant.	Rel. Quant.	Band %	Lane %	Norm. Factor	Norm. Vol. (Int)
Chemi Hi Resolution	1		N/A	0,320	28.178.325	30.696.498	N/A	N/A	100,0	94,9	N/A	N/A

Band Detection	Automatically detected bands with sensitivity: Low
Lane Background	Lane background subtracted with disk size: 10
Lane Width	7.51 mm

Lane 2

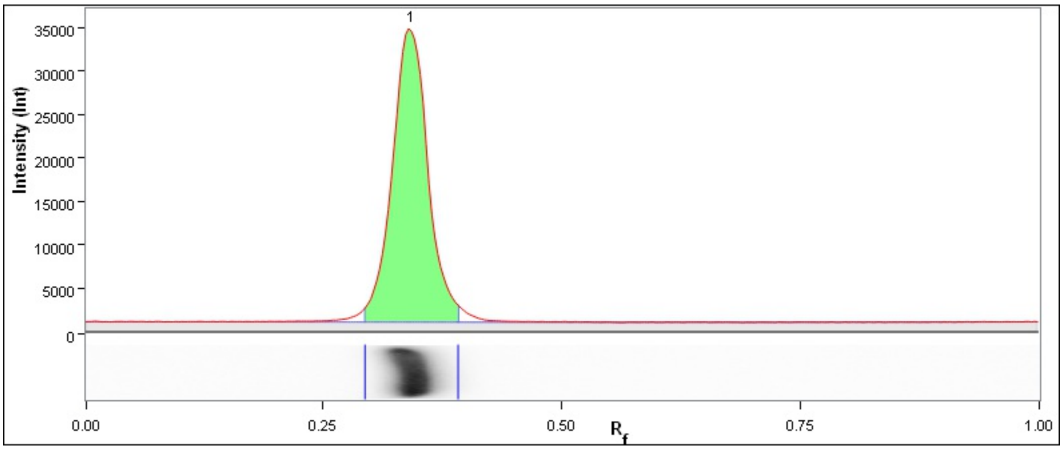

Channel	Band No.	Band Label	Mol. Wt. (KDa)	Relative Front	Adj. Volume (Int)	Volume (Int)	Abs. Quant.	Rel. Quant.	Band %	Lane %	Norm. Factor	Norm. Vol. (Int)
Chemi Hi Resolution	1		N/A	0,345	33.162.759	35.700.651	N/A	N/A	100,0	95,0	N/A	N/A

Band Detection	Automatically detected bands with sensitivity: Low
Lane Background	Lane background subtracted with disk size: 10
Lane Width	7.51 mm

Lane 3

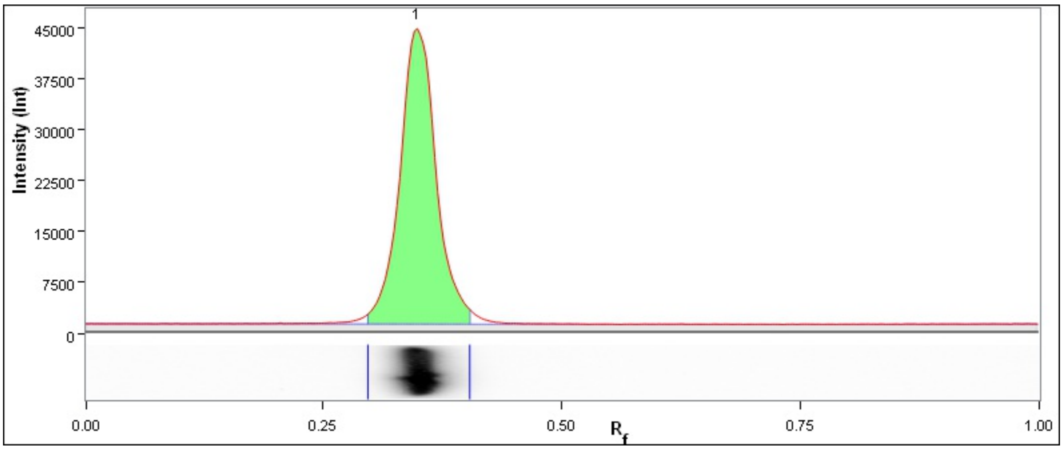

Channel	Band No.	Band Label	Mol. Wt. (KDa)	Relative Front	Adj. Volume (Int)	Volume (Int)	Abs. Quant.	Rel. Quant.	Band %	Lane %	Norm. Factor	Norm. Vol. (Int)
Chemi Hi Resolution	1		N/A	0,351	43.945.572	46.747.074	N/A	N/A	100,0	95,8	N/A	N/A

Band Detection	Automatically detected bands with sensitivity: Low
Lane Background	Lane background subtracted with disk size: 10
Lane Width	7.87 mm

Lane 4

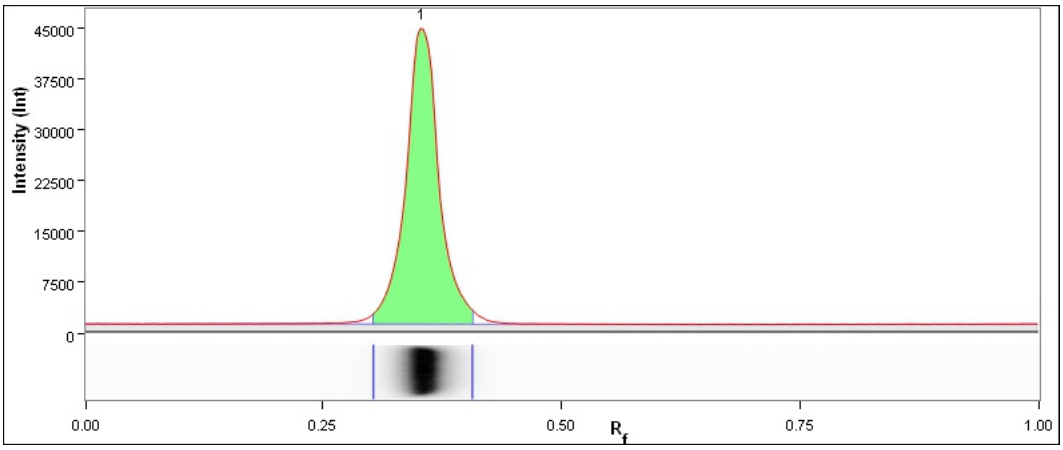

Channel	Band No.	Band Label	Mol. Wt. (KDa)	Relative Front	Adj. Volume (Int)	Volume (Int)	Abs. Quant.	Rel. Quant.	Band %	Lane %	Norm. Factor	Norm. Vol. (Int)
Chemi Hi Resolution	1		N/A	0,357	37.017.120	39.484.140	N/A	N/A	100,0	95,8	N/A	N/A

Band Detection	Automatically detected bands with sensitivity: Low
Lane Background	Lane background subtracted with disk size: 10
Lane Width	7.16 mm

Lane 5

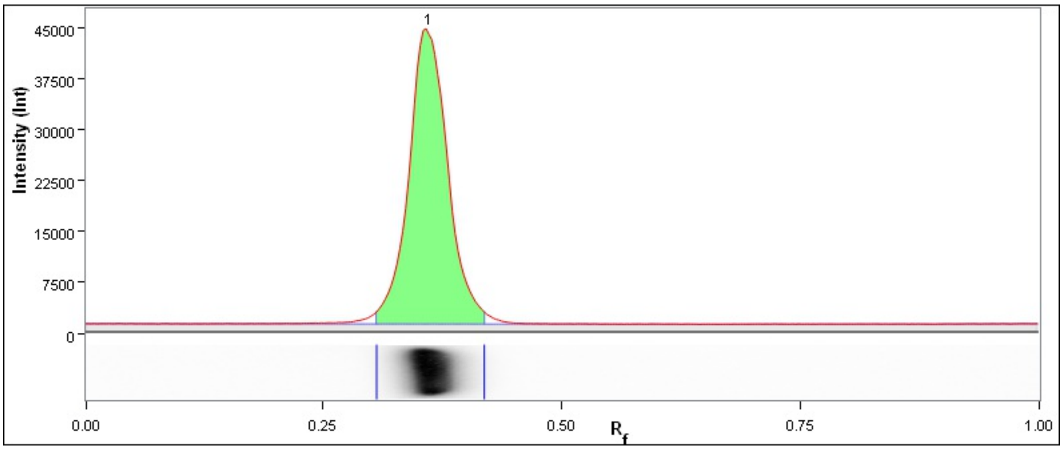

Channel	Band No.	Band Label	Mol. Wt. (KDa)	Relative Front	Adj. Volume (Int)	Volume (Int)	Abs. Quant.	Rel. Quant.	Band %	Lane %	Norm. Factor	Norm. Vol. (Int)
Chemi Hi Resolution	1		N/A	0,363	43.929.963	46.707.318	N/A	N/A	100,0	96,1	N/A	N/A

Band Detection	Automatically detected bands with sensitivity: Low
Lane Background	Lane background subtracted with disk size: 10
Lane Width	7.51 mm

Lane 6

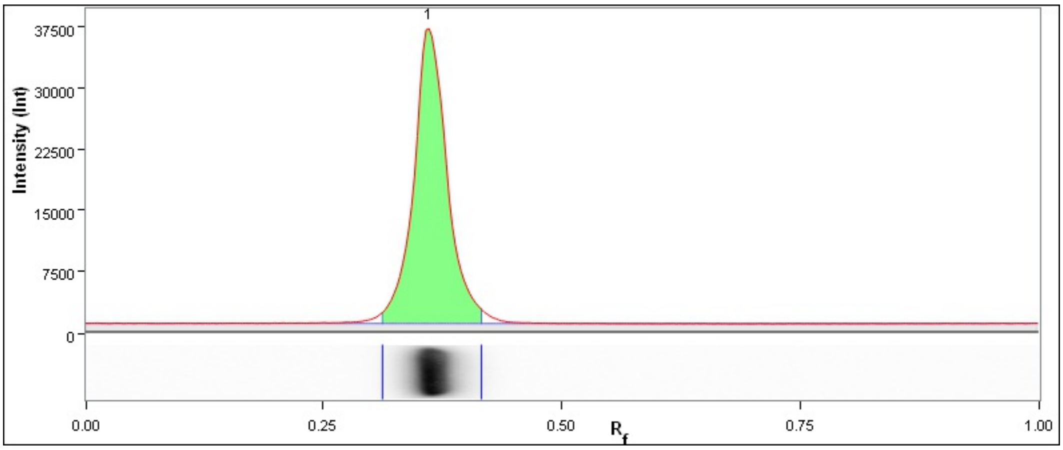

Channel	Band No.	Band Label	Mol. Wt. (KDa)	Relative Front	Adj. Volume (Int)	Volume (Int)	Abs. Quant.	Rel. Quant.	Band %	Lane %	Norm. Factor	Norm. Vol. (Int)
Chemi Hi Resolution	1		N/A	0,363	35.861.805	38.501.694	N/A	N/A	100,0	96,1	N/A	N/A

Band Detection	Automatically detected bands with sensitivity: Low
Lane Background	Lane background subtracted with disk size: 10
Lane Width	7.51 mm

Lane 7

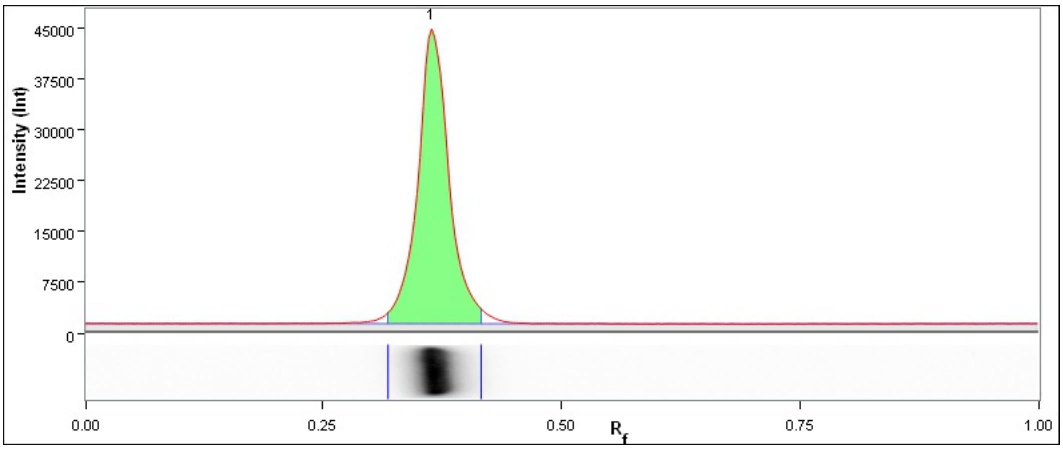

Channel	Band No.	Band Label	Mol. Wt. (KDa)	Relative Front	Adj. Volume (Int)	Volume (Int)	Abs. Quant.	Rel. Quant.	Band %	Lane %	Norm. Factor	Norm. Vol. (Int)
Chemi Hi Resolution	1		N/A	0,366	36.220.086	38.773.413	N/A	N/A	100,0	95,7	N/A	N/A

Band Detection	Automatically detected bands with sensitivity: Low
Lane Background	Lane background subtracted with disk size: 10
Lane Width	7.51 mm

Lane 8

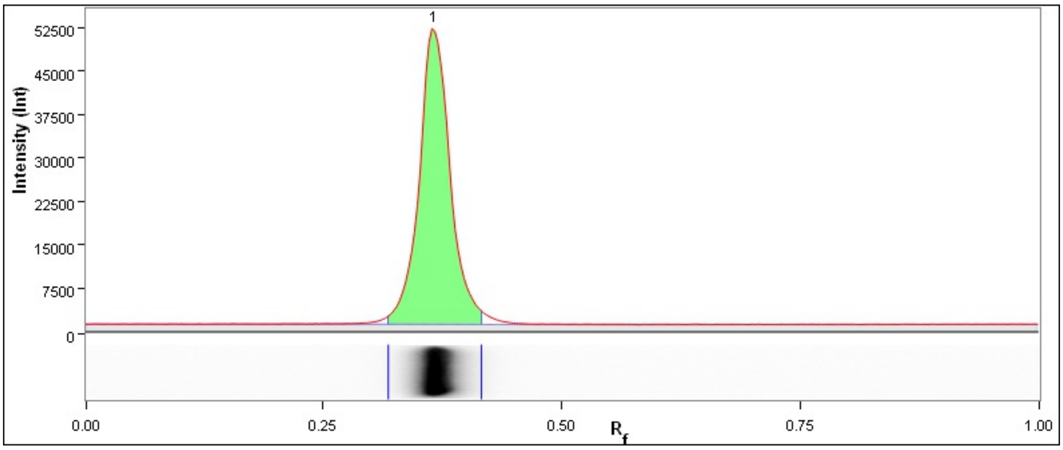

Channel	Band No.	Band Label	Mol. Wt. (KDa)	Relative Front	Adj. Volume (Int)	Volume (Int)	Abs. Quant.	Rel. Quant.	Band %	Lane %	Norm. Factor	Norm. Vol. (Int)
Chemi Hi Resolution	1		N/A	0,369	44.124.810	47.091.258	N/A	N/A	100,0	95,9	N/A	N/A

Band Detection	Automatically detected bands with sensitivity: Low
Lane Background	Lane background subtracted with disk size: 10
Lane Width	8.23 mm
