# Supplementary material for: Multimodal Imaging Techniques to Evaluate the Anticancer Effect of Cold Atmospheric Pressure Plasma
Source: Cancers (Basel). 2021 May 19;13(10):2483. doi: 10.3390/cancers13102483 (PMC8161248; doi:10.3390/cancers13102483)
Supplement: Supplementary file 1 [file cancers-13-02483-s001.zip › cancers-1174396-supplementary/Figure S2. Original Western Blot images/SCC cell line A431 cleaved-caspase-3 ß-actin.pdf]

Image Report: 20200611 Cleaved Caspase 3 Eva S3 60µg Prot

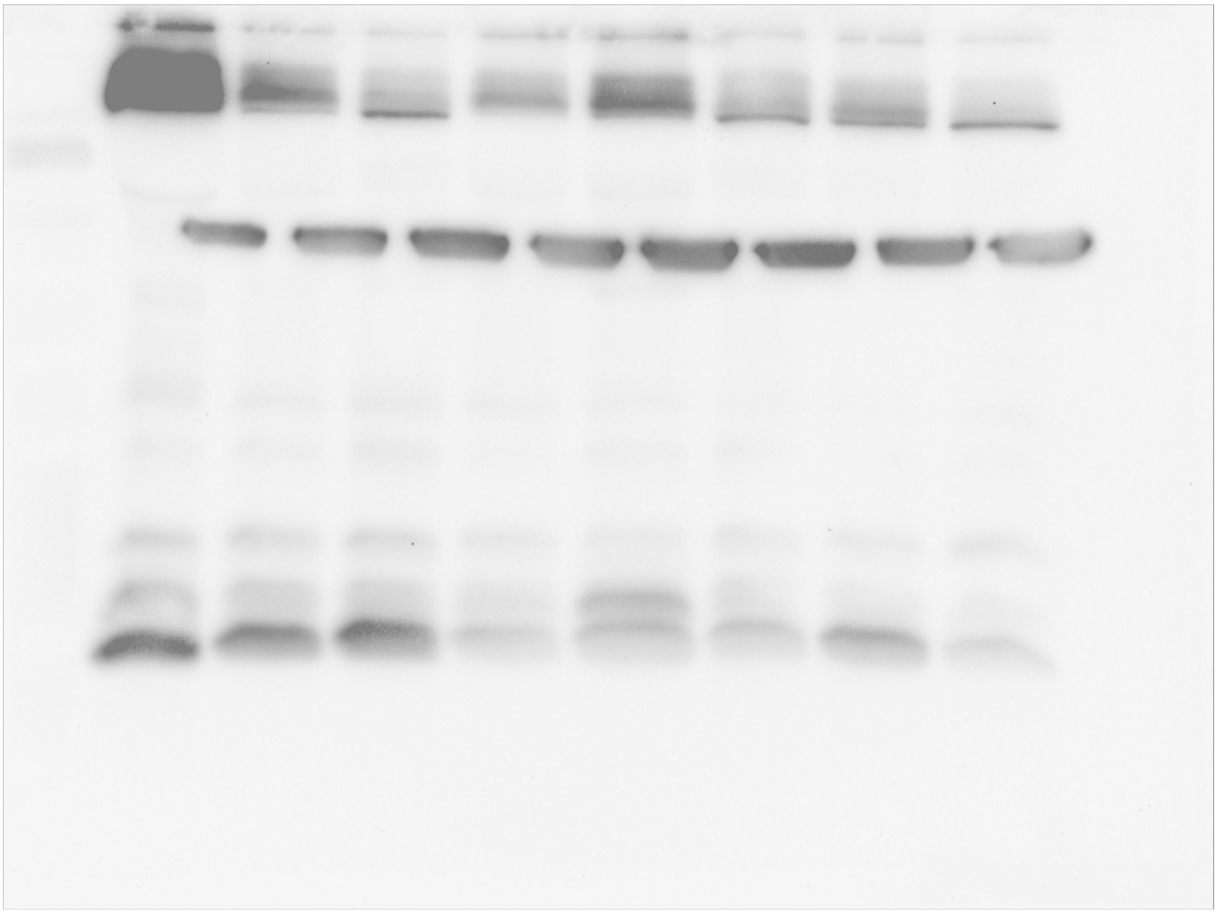

C:\Users\marcel.kordt\Desktop\Marcel\Versuche\Probenaufbereitung\Mol\WB März 2020 mit und ohne KAP\Auswertung Finall\20200611 Cleaved Caspase 3 Eva S3 60µg Prot.mscn

---

Channel 1 - Red - Chemi Hi Resolution

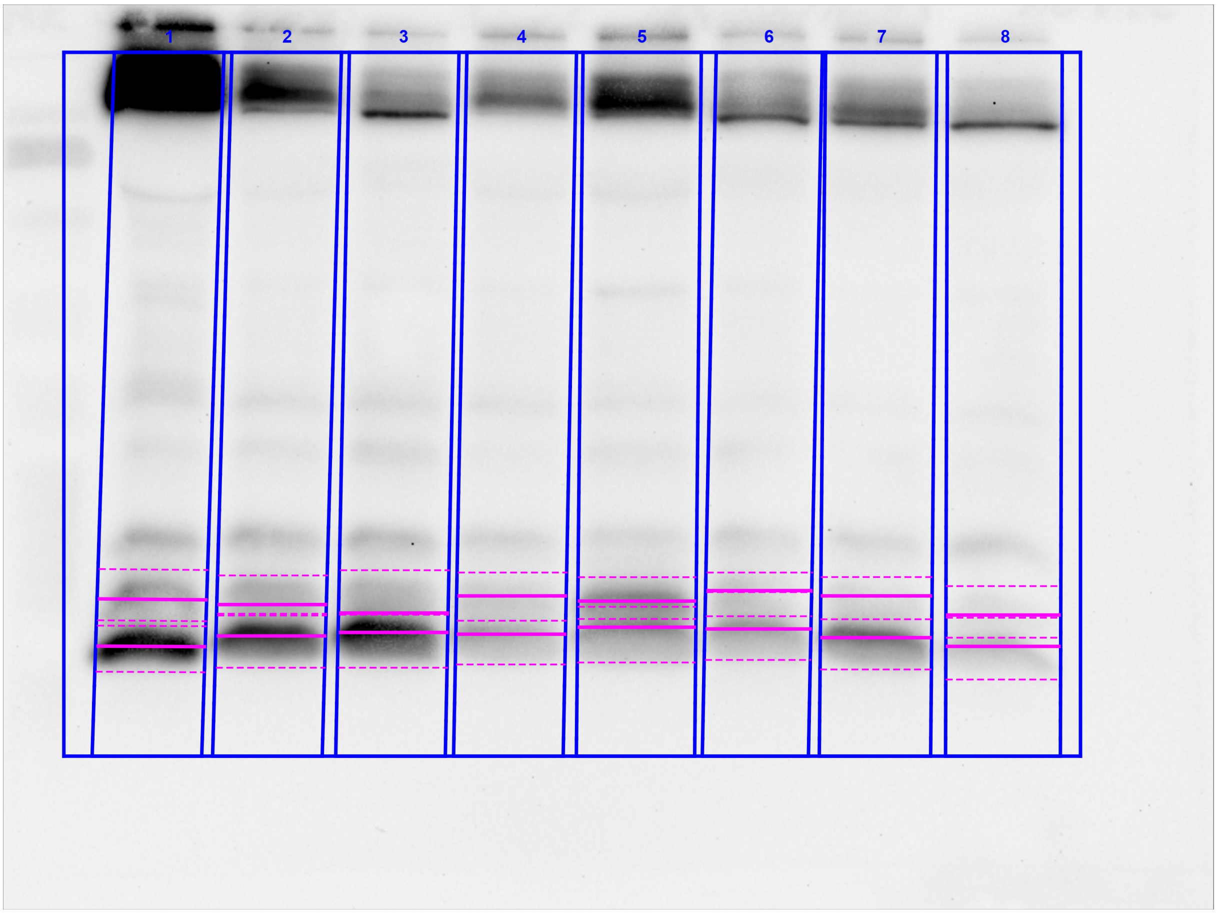

Lane Statistics

Channel	Lane No.	Adj. Total Band Vol. (Int)	Total Band Vol. (Int)	Adj. Total Lane Vol. (Int)	Total Lane Vol. (Int)	Bkgd. Vol. (Int)	Norm. Factor
Chemi Hi Resolution	1	12.637.926	15.553.692	76.579.524	111.840.624	35.261.100	N/A
Chemi Hi Resolution	2	10.368.162	12.731.544	23.460.318	39.634.623	16.174.305	N/A
Chemi Hi Resolution	3	11.256.840	13.853.700	19.927.719	34.393.905	14.466.186	N/A
Chemi Hi Resolution	4	2.684.367	4.145.400	10.516.023	23.853.753	13.337.730	N/A
Chemi Hi Resolution	5	7.344.748	8.618.184	24.247.032	38.805.900	14.558.868	N/A
Chemi Hi Resolution	6	2.794.532	4.125.613	10.969.874	23.650.249	12.680.375	N/A
Chemi Hi Resolution	7	5.115.776	6.425.984	14.309.952	26.175.488	11.865.536	N/A
Chemi Hi Resolution	8	1.701.480	2.887.236	8.536.176	19.923.486	11.387.310	N/A

Lane And Band Analysis

Lane 1

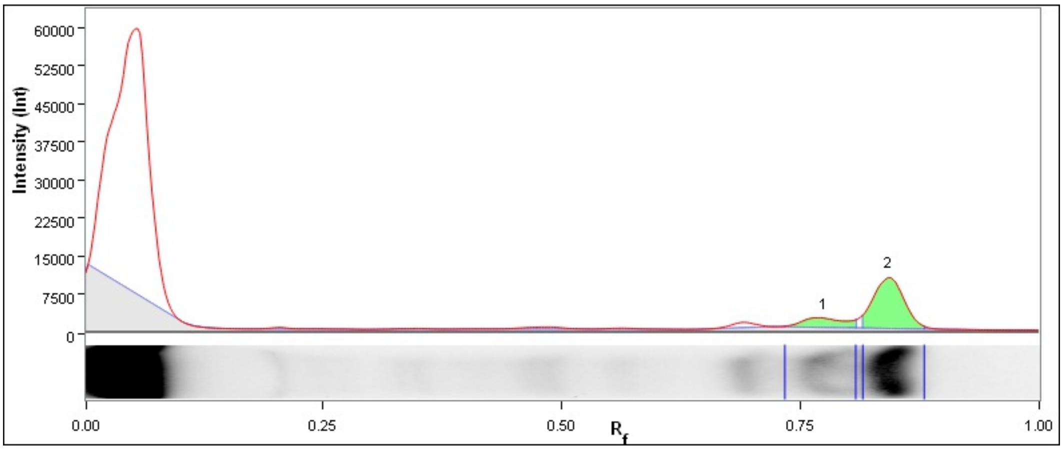

Channel	Band No.	Band Label	Mol. Wt. (KDa)	Relative Front	Adj. Volume (Int)	Volume (Int)	Abs. Quant.	Rel. Quant.	Band %	Lane %	Norm. Factor	Norm. Vol. (Int)
Chemi Hi Resolution	1		N/A	0,777	2.751.966	4.494.798	N/A	N/A	21,8	3,6	N/A	N/A
Chemi Hi Resolution	2		N/A	0,844	9.885.960	11.058.894	N/A	N/A	78,2	12,9	N/A	N/A

Band Detection	Automatically detected bands with sensitivity: Low
Lane Background	Lane background subtracted with disk size: 10
Lane Width	7.51 mm

Lane 2

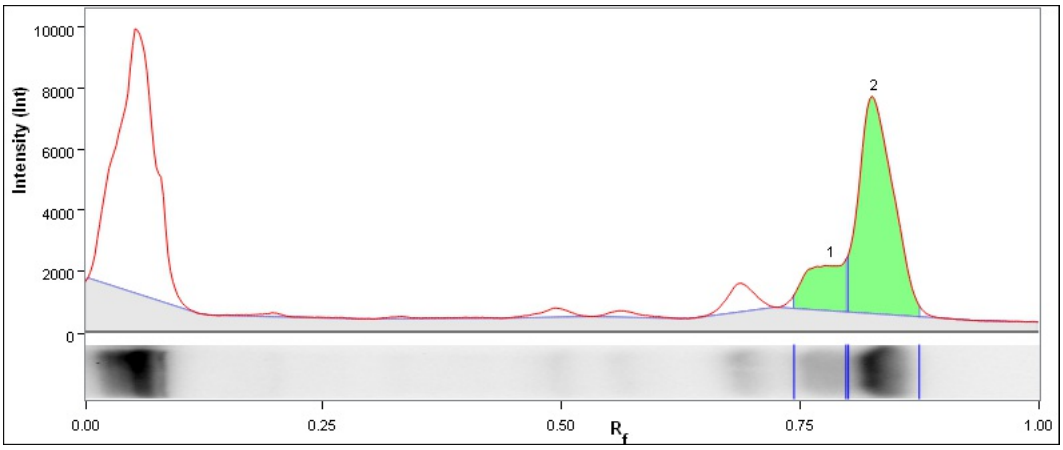

Channel	Band No.	Band Label	Mol. Wt. (KDa)	Relative Front	Adj. Volume (Int)	Volume (Int)	Abs. Quant.	Rel. Quant.	Band %	Lane %	Norm. Factor	Norm. Vol. (Int)
Chemi Hi Resolution	1		N/A	0,785	2.050.083	3.167.451	N/A	N/A	19,8	8,7	N/A	N/A
Chemi Hi Resolution	2		N/A	0,829	8.318.079	9.564.093	N/A	N/A	80,2	35,5	N/A	N/A

Band Detection	Automatically detected bands with sensitivity: Low
Lane Background	Lane background subtracted with disk size: 10
Lane Width	7.51 mm

Lane 3

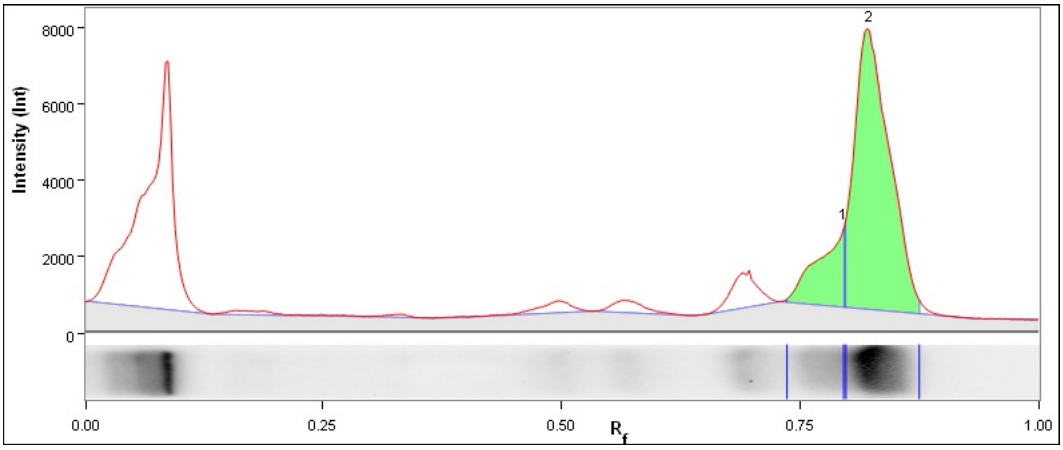

Channel	Band No.	Band Label	Mol. Wt. (KDa)	Relative Front	Adj. Volume (Int)	Volume (Int)	Abs. Quant.	Rel. Quant.	Band %	Lane %	Norm. Factor	Norm. Vol. (Int)
Chemi Hi Resolution	1		N/A	0,797	2.085.741	3.412.332	N/A	N/A	18,5	10,5	N/A	N/A
Chemi Hi Resolution	2		N/A	0,824	9.171.099	10.441.368	N/A	N/A	81,5	46,0	N/A	N/A

Band Detection	Automatically detected bands with sensitivity: Low
Lane Background	Lane background subtracted with disk size: 10
Lane Width	7.51 mm

Lane 4

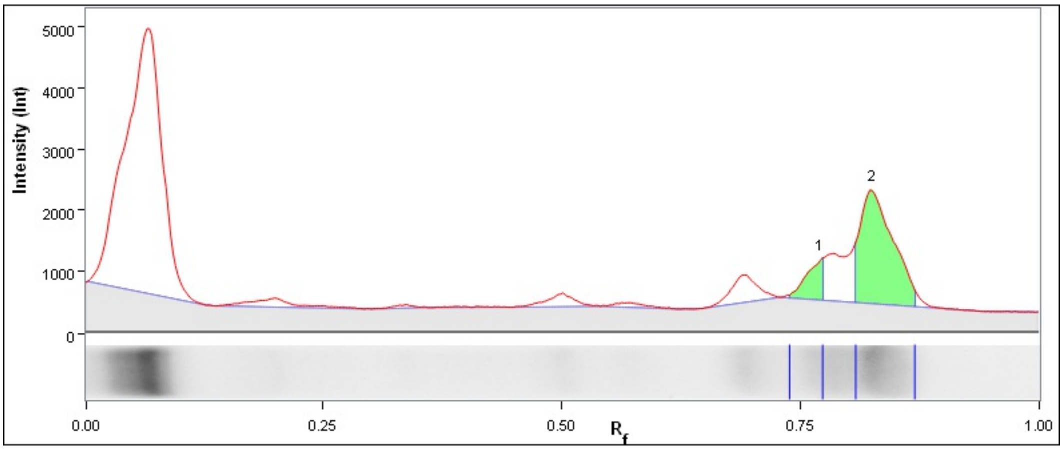

Channel	Band No.	Band Label	Mol. Wt. (KDa)	Relative Front	Adj. Volume (Int)	Volume (Int)	Abs. Quant.	Rel. Quant.	Band %	Lane %	Norm. Factor	Norm. Vol. (Int)
Chemi Hi Resolution	1		N/A	0,772	465.003	1.063.125	N/A	N/A	17,3	4,4	N/A	N/A
Chemi Hi Resolution	2		N/A	0,827	2.219.364	3.082.275	N/A	N/A	82,7	21,1	N/A	N/A

Band Detection	Automatically detected bands with sensitivity: Low
Lane Background	Lane background subtracted with disk size: 10
Lane Width	7.51 mm

Lane 5

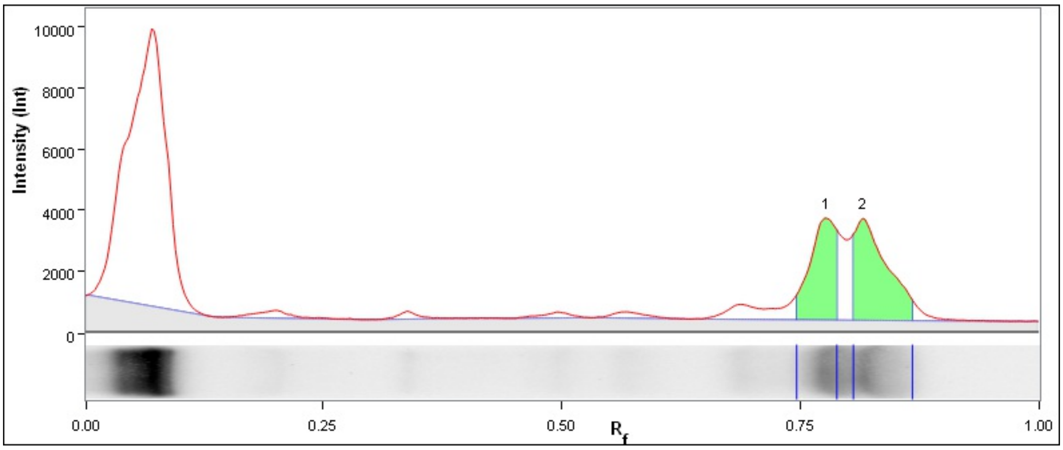

Channel	Band No.	Band Label	Mol. Wt. (KDa)	Relative Front	Adj. Volume (Int)	Volume (Int)	Abs. Quant.	Rel. Quant.	Band %	Lane %	Norm. Factor	Norm. Vol. (Int)
Chemi Hi Resolution	1		N/A	0,780	3.241.356	3.775.428	N/A	N/A	44,1	13,4	N/A	N/A
Chemi Hi Resolution	2		N/A	0,817	4.103.392	4.842.756	N/A	N/A	55,9	16,9	N/A	N/A

Band Detection	Automatically detected bands with sensitivity: Low
Lane Background	Lane background subtracted with disk size: 10
Lane Width	8.11 mm

Lane 6

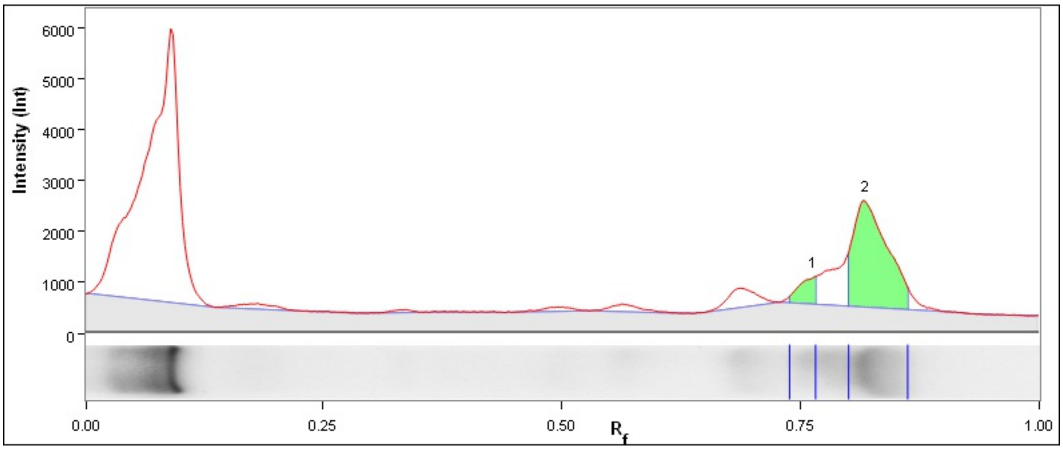

Channel	Band No.	Band Label	Mol. Wt. (KDa)	Relative Front	Adj. Volume (Int)	Volume (Int)	Abs. Quant.	Rel. Quant.	Band %	Lane %	Norm. Factor	Norm. Vol. (Int)
Chemi Hi Resolution	1		N/A	0,765	348.554	818.681	N/A	N/A	12,5	3,2	N/A	N/A
Chemi Hi Resolution	2		N/A	0,819	2.445.978	3.306.932	N/A	N/A	87,5	22,3	N/A	N/A

Band Detection	Automatically detected bands with sensitivity: Low
Lane Background	Lane background subtracted with disk size: 10
Lane Width	7.27 mm

Lane 7

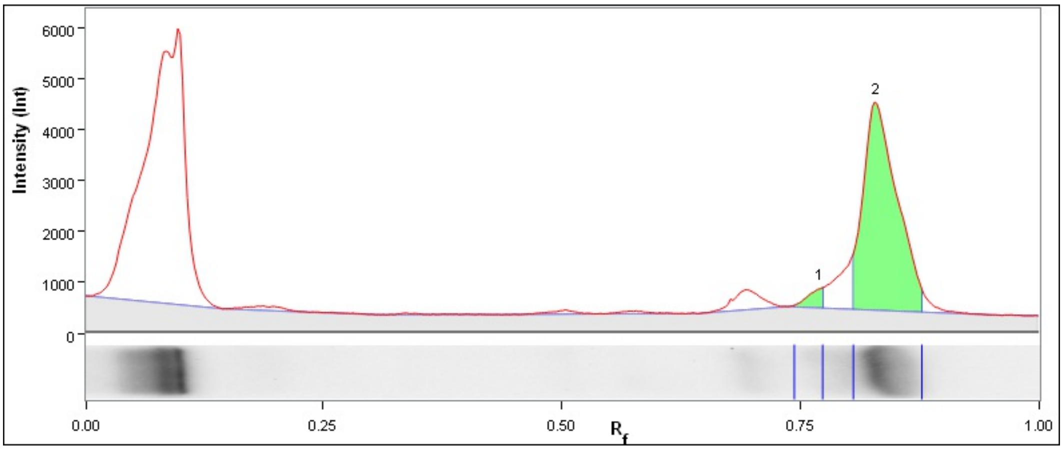

Channel	Band No.	Band Label	Mol. Wt. (KDa)	Relative Front	Adj. Volume (Int)	Volume (Int)	Abs. Quant.	Rel. Quant.	Band %	Lane %	Norm. Factor	Norm. Vol. (Int)
Chemi Hi Resolution	1		N/A	0,772	238.144	683.904	N/A	N/A	4,7	1,7	N/A	N/A
Chemi Hi Resolution	2		N/A	0,832	4.877.632	5.742.080	N/A	N/A	95,3	34,1	N/A	N/A

Band Detection	Automatically detected bands with sensitivity: Low
Lane Background	Lane background subtracted with disk size: 10
Lane Width	7.63 mm

Lane 8

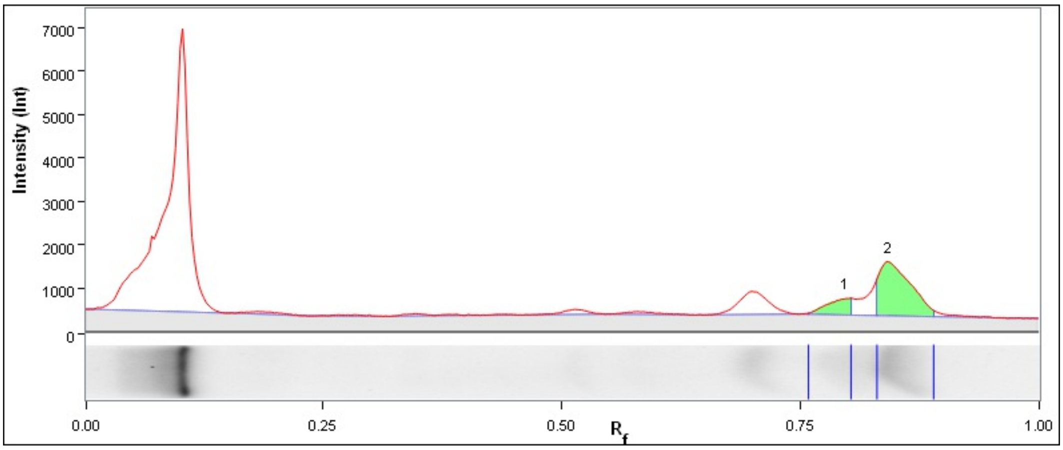

Channel	Band No.	Band Label	Mol. Wt. (KDa)	Relative Front	Adj. Volume (Int)	Volume (Int)	Abs. Quant.	Rel. Quant.	Band %	Lane %	Norm. Factor	Norm. Vol. (Int)
Chemi Hi Resolution	1		N/A	0,800	351.978	889.284	N/A	N/A	20,7	4,1	N/A	N/A
Chemi Hi Resolution	2		N/A	0,844	1.349.502	1.997.952	N/A	N/A	79,3	15,8	N/A	N/A

Band Detection	Automatically detected bands with sensitivity: Low
Lane Background	Lane background subtracted with disk size: 10
Lane Width	7.87 mm

Channel 2 - Green - Chemi Hi Resolution

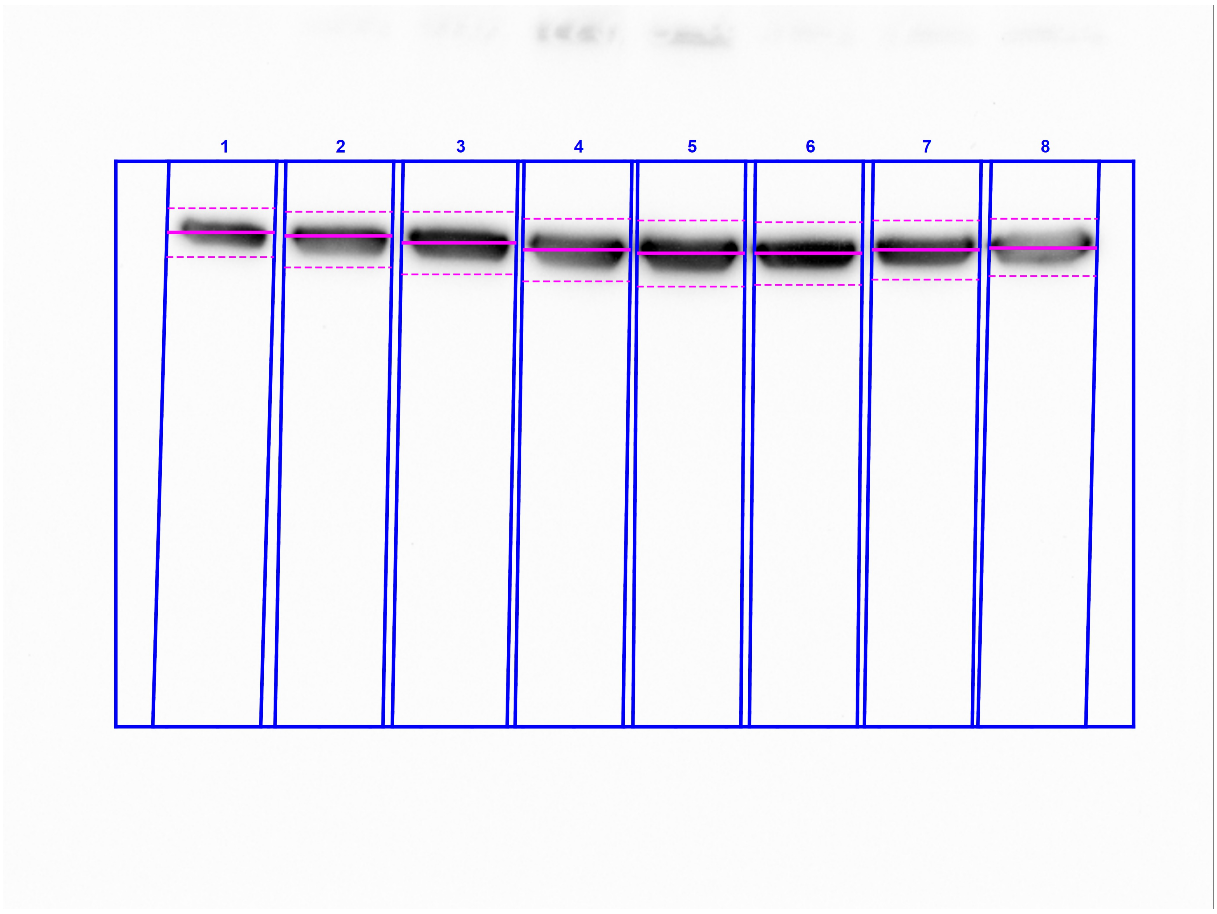

Lane Statistics

Channel	Lane No.	Adj. Total Band Vol. (Int)	Total Band Vol. (Int)	Adj. Total Lane Vol. (Int)	Total Lane Vol. (Int)	Bkgd. Vol. (Int)	Norm. Factor
Chemi Hi Resolution	1	25.204.736	27.129.216	26.402.452	47.374.262	20.971.810	N/A
Chemi Hi Resolution	2	32.106.328	34.326.796	33.351.846	54.596.890	21.245.044	N/A
Chemi Hi Resolution	3	43.403.250	46.105.884	44.790.372	67.679.172	22.888.800	N/A
Chemi Hi Resolution	4	37.713.360	40.247.672	39.367.582	60.869.244	21.501.662	N/A
Chemi Hi Resolution	5	48.006.848	50.707.506	49.725.922	71.407.260	21.681.338	N/A
Chemi Hi Resolution	6	47.784.020	50.319.262	49.275.244	70.805.488	21.530.244	N/A
Chemi Hi Resolution	7	37.853.728	40.192.182	39.247.984	60.280.740	21.032.756	N/A
Chemi Hi Resolution	8	27.391.848	29.616.780	28.559.308	49.251.188	20.691.880	N/A

Lane And Band Analysis

Lane 1

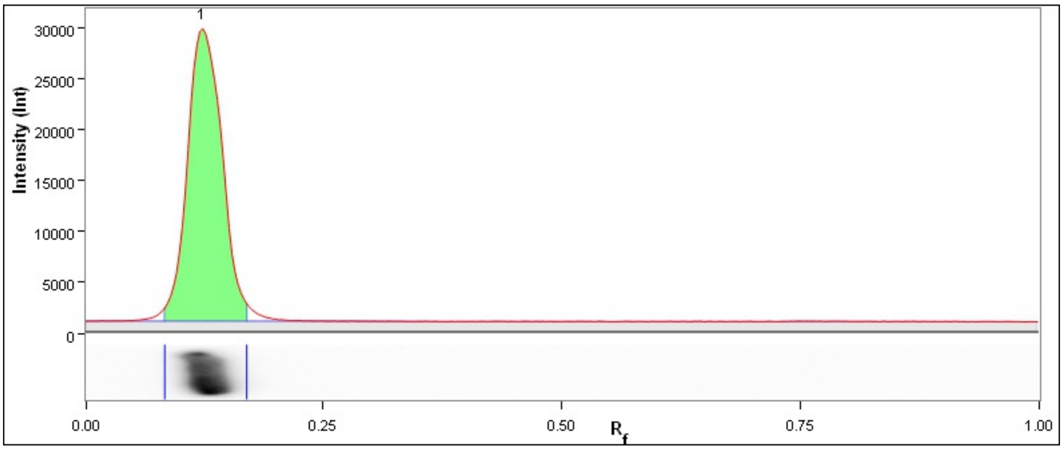

Channel	Band No.	Band Label	Mol. Wt. (KDa)	Relative Front	Adj. Volume (Int)	Volume (Int)	Abs. Quant.	Rel. Quant.	Band %	Lane %	Norm. Factor	Norm. Vol. (Int)
Chemi Hi Resolution	1		N/A	0,126	25.204.736	27.129.216	N/A	N/A	100,0	95,5	N/A	N/A

Band Detection	Automatically detected bands with sensitivity: Low
Lane Background	Lane background subtracted with disk size: 10
Lane Width	7.39 mm

Lane 2

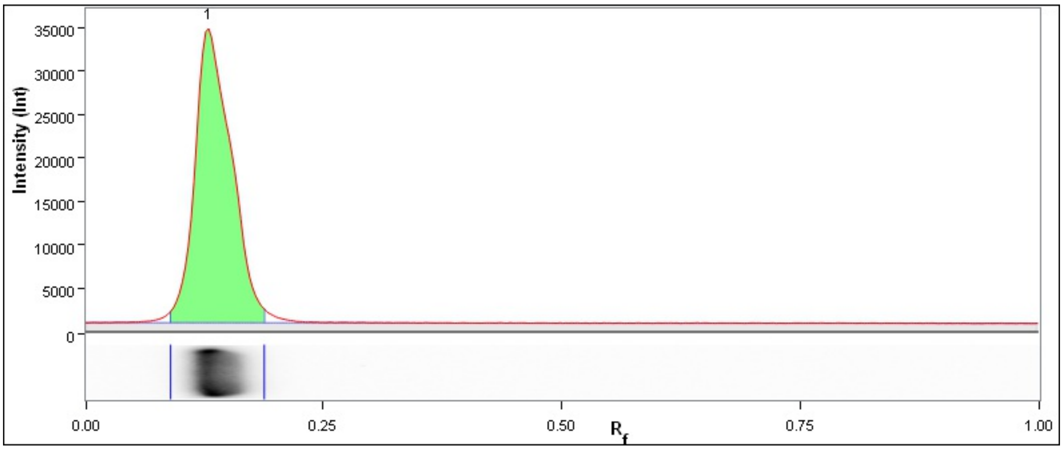

Channel	Band No.	Band Label	Mol. Wt. (KDa)	Relative Front	Adj. Volume (Int)	Volume (Int)	Abs. Quant.	Rel. Quant.	Band %	Lane %	Norm. Factor	Norm. Vol. (Int)
Chemi Hi Resolution	1		N/A	0,132	32.106.328	34.326.796	N/A	N/A	100,0	96,3	N/A	N/A

Band Detection	Automatically detected bands with sensitivity: Low
Lane Background	Lane background subtracted with disk size: 10
Lane Width	7.39 mm

Lane 3

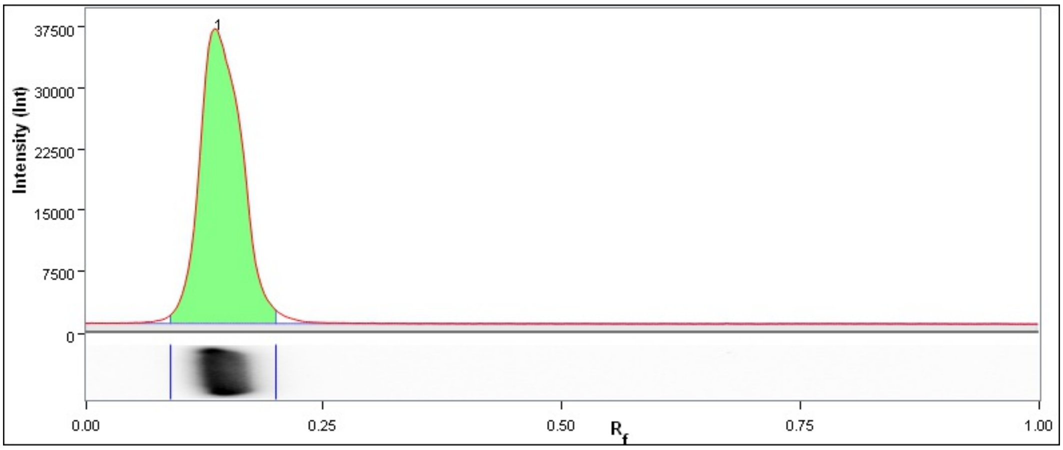

Channel	Band No.	Band Label	Mol. Wt. (KDa)	Relative Front	Adj. Volume (Int)	Volume (Int)	Abs. Quant.	Rel. Quant.	Band %	Lane %	Norm. Factor	Norm. Vol. (Int)
Chemi Hi Resolution	1		N/A	0,144	43.403.250	46.105.884	N/A	N/A	100,0	96,9	N/A	N/A

Band Detection	Automatically detected bands with sensitivity: Low
Lane Background	Lane background subtracted with disk size: 10
Lane Width	7.87 mm

Lane 4

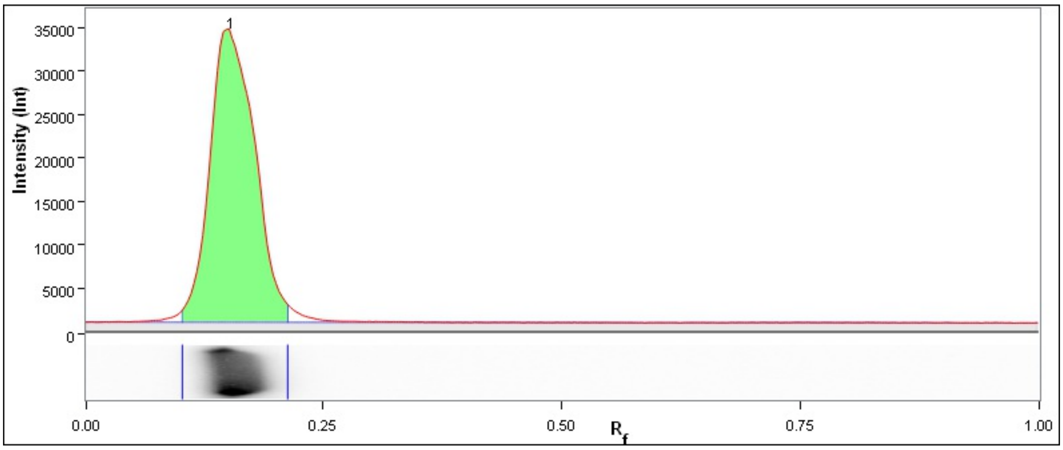

Channel	Band No.	Band Label	Mol. Wt. (KDa)	Relative Front	Adj. Volume (Int)	Volume (Int)	Abs. Quant.	Rel. Quant.	Band %	Lane %	Norm. Factor	Norm. Vol. (Int)
Chemi Hi Resolution	1		N/A	0,156	37.713.360	40.247.672	N/A	N/A	100,0	95,8	N/A	N/A

Band Detection	Automatically detected bands with sensitivity: Low
Lane Background	Lane background subtracted with disk size: 10
Lane Width	7.39 mm

Lane 5

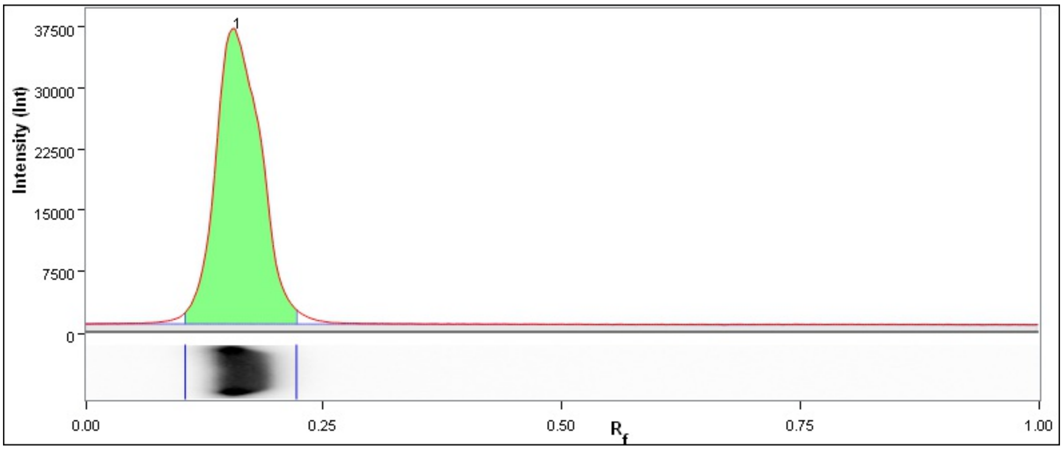

Channel	Band No.	Band Label	Mol. Wt. (KDa)	Relative Front	Adj. Volume (Int)	Volume (Int)	Abs. Quant.	Rel. Quant.	Band %	Lane %	Norm. Factor	Norm. Vol. (Int)
Chemi Hi Resolution	1		N/A	0,163	48.006.848	50.707.506	N/A	N/A	100,0	96,5	N/A	N/A

Band Detection	Automatically detected bands with sensitivity: Low
Lane Background	Lane background subtracted with disk size: 10
Lane Width	7.39 mm

Lane 6

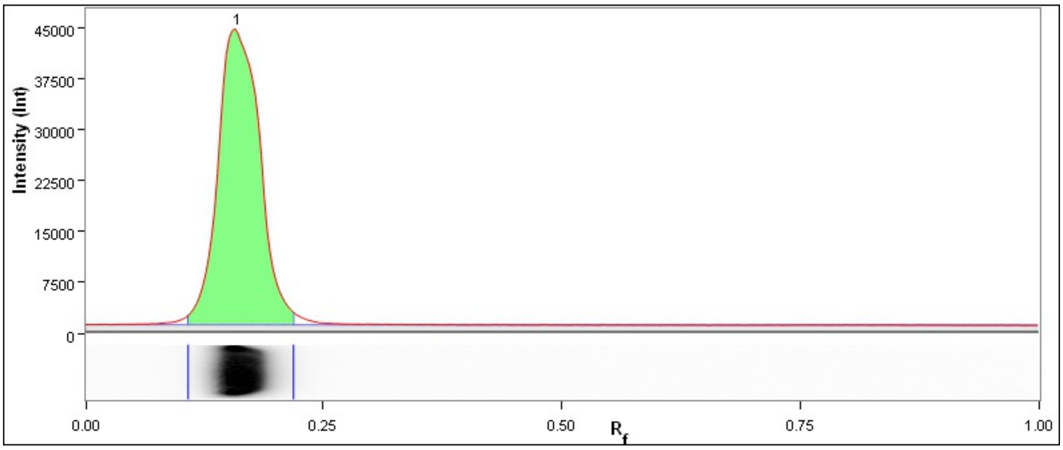

Channel	Band No.	Band Label	Mol. Wt. (KDa)	Relative Front	Adj. Volume (Int)	Volume (Int)	Abs. Quant.	Rel. Quant.	Band %	Lane %	Norm. Factor	Norm. Vol. (Int)
Chemi Hi Resolution	1		N/A	0,163	47.784.020	50.319.262	N/A	N/A	100,0	97,0	N/A	N/A

Band Detection	Automatically detected bands with sensitivity: Low
Lane Background	Lane background subtracted with disk size: 10
Lane Width	7.39 mm

Lane 7

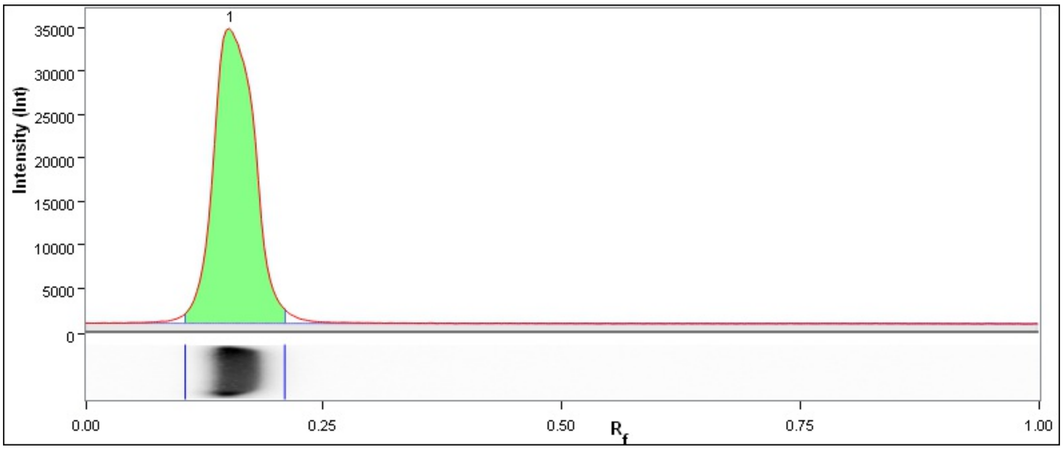

Channel	Band No.	Band Label	Mol. Wt. (KDa)	Relative Front	Adj. Volume (Int)	Volume (Int)	Abs. Quant.	Rel. Quant.	Band %	Lane %	Norm. Factor	Norm. Vol. (Int)
Chemi Hi Resolution	1		N/A	0,156	37.853.728	40.192.182	N/A	N/A	100,0	96,4	N/A	N/A

Band Detection	Automatically detected bands with sensitivity: Low
Lane Background	Lane background subtracted with disk size: 10
Lane Width	7.39 mm

Lane 8

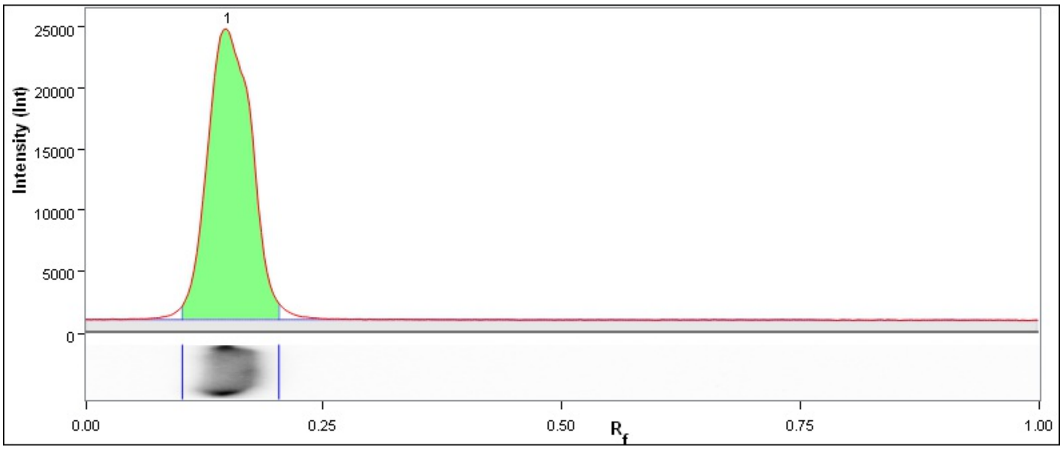

Channel	Band No.	Band Label	Mol. Wt. (KDa)	Relative Front	Adj. Volume (Int)	Volume (Int)	Abs. Quant.	Rel. Quant.	Band %	Lane %	Norm. Factor	Norm. Vol. (Int)
Chemi Hi Resolution	1		N/A	0,153	27.391.848	29.616.780	N/A	N/A	100,0	95,9	N/A	N/A

Band Detection	Automatically detected bands with sensitivity: Low
Lane Background	Lane background subtracted with disk size: 10
Lane Width	7.39 mm
