# Supplementary material for: Multimodal Imaging Techniques to Evaluate the Anticancer Effect of Cold Atmospheric Pressure Plasma
Source: Cancers (Basel). 2021 May 19;13(10):2483. doi: 10.3390/cancers13102483 (PMC8161248; doi:10.3390/cancers13102483)
Supplement: Supplementary file 1 [file cancers-13-02483-s001.zip › cancers-1174396-supplementary/Figure S2. Original Western Blot images/SCC cell line A431 PCNA ß-actin.pdf]

Image Report: 20200427 PCNA ab29 wdh Eva S2

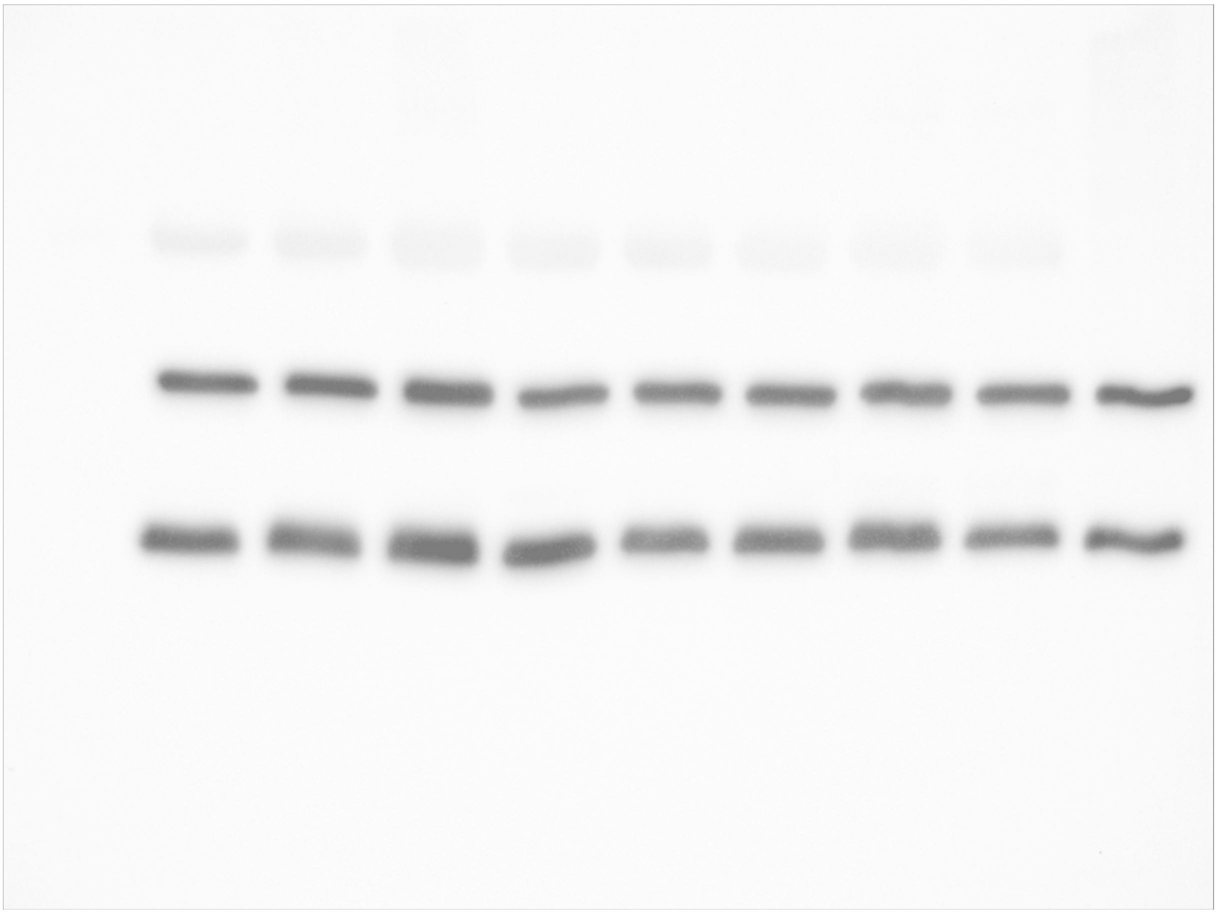

C:\Users\marcel.kordt\Desktop\Marcel\Versuche\Probenaufbereitung\Mol\WB März 2020 mit und ohne KAP\Auswertung Finall\20200427 PCNA ab29 wdh Eva S2.mscn

---

Channel 1 - Red - Chemi Hi Resolution

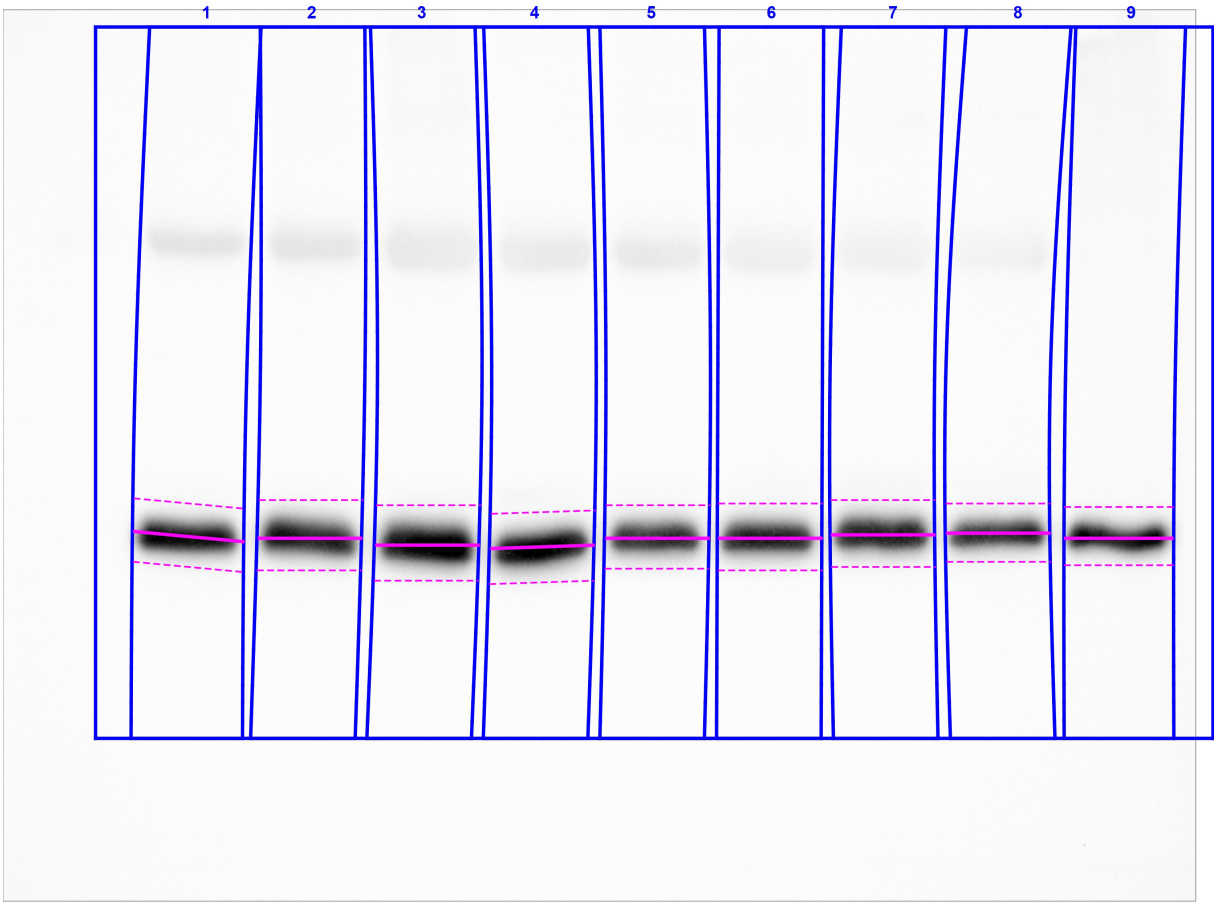

Lane Statistics

Channel	Lane No.	Adj. Total Band Vol. (Int)	Total Band Vol. (Int)	Adj. Total Lane Vol. (Int)	Total Lane Vol. (Int)	Bkgd. Vol. (Int)	Norm. Factor
Chemi Hi Resolution	1	50.189.165	53.702.155	57.764.915	97.331.260	39.566.345	N/A
Chemi Hi Resolution	2	50.304.870	53.811.638	58.177.530	94.577.389	36.399.859	N/A
Chemi Hi Resolution	3	62.494.073	66.285.589	70.635.377	108.268.534	37.633.157	N/A
Chemi Hi Resolution	4	53.556.048	57.010.112	61.638.426	96.078.233	34.439.807	N/A
Chemi Hi Resolution	5	40.397.372	43.467.746	47.624.286	82.339.630	34.715.344	N/A
Chemi Hi Resolution	6	44.992.258	48.253.074	51.522.430	86.507.638	34.985.208	N/A
Chemi Hi Resolution	7	47.356.374	50.819.466	53.736.913	90.556.269	36.819.356	N/A
Chemi Hi Resolution	8	38.288.053	41.428.638	43.661.299	82.479.137	38.817.838	N/A
Chemi Hi Resolution	9	42.299.328	46.046.848	45.058.944	93.959.040	48.900.096	N/A

Lane And Band Analysis

Lane 1

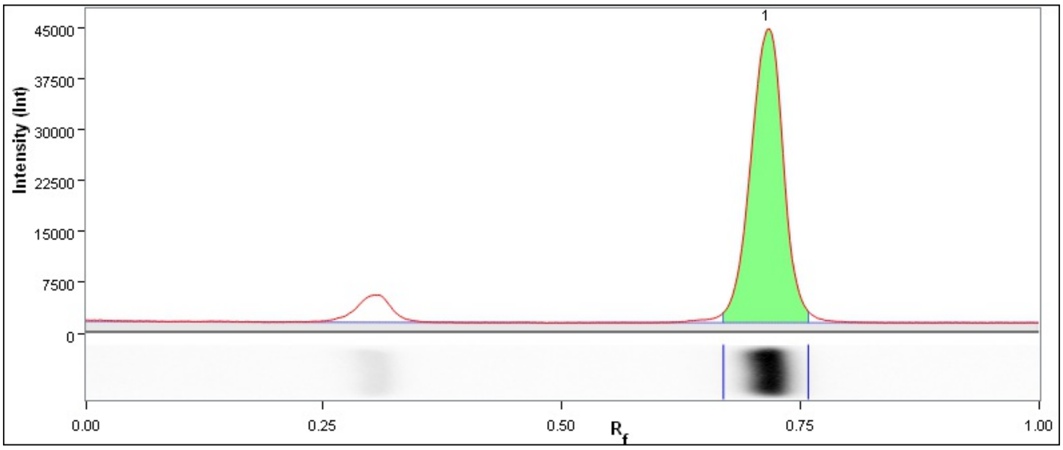

Channel	Band No.	Band Label	Mol. Wt. (KDa)	Relative Front	Adj. Volume (Int)	Volume (Int)	Abs. Quant.	Rel. Quant.	Band %	Lane %	Norm. Factor	Norm. Vol. (Int)
Chemi Hi Resolution	1		N/A	0,716	50.189.165	53.702.155	N/A	N/A	100,0	86,9	N/A	N/A

Band Detection	Automatically detected bands with sensitivity: Low
Lane Background	Lane background subtracted with disk size: 10
Lane Width	7.75 mm

Lane 2

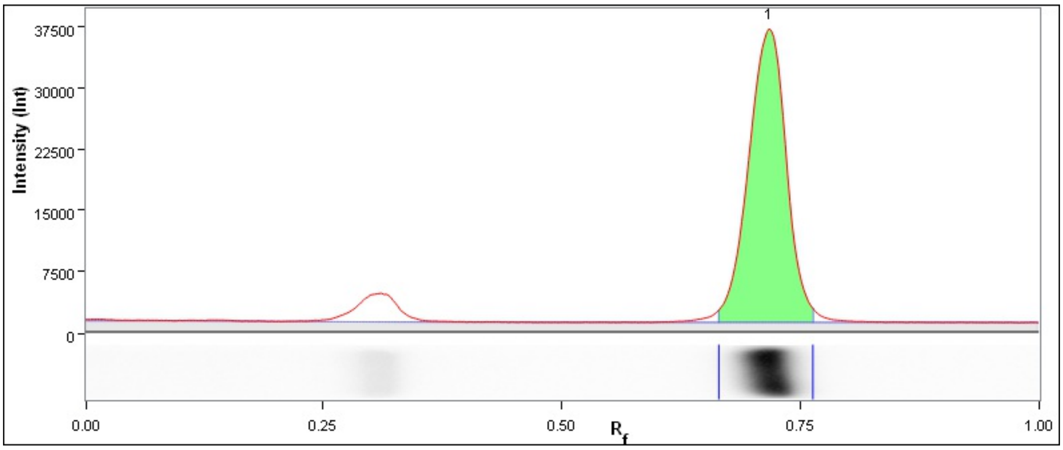

Channel	Band No.	Band Label	Mol. Wt. (KDa)	Relative Front	Adj. Volume (Int)	Volume (Int)	Abs. Quant.	Rel. Quant.	Band %	Lane %	Norm. Factor	Norm. Vol. (Int)
Chemi Hi Resolution	1		N/A	0,719	50.304.870	53.811.638	N/A	N/A	100,0	86,5	N/A	N/A

Band Detection	Automatically detected bands with sensitivity: Low
Lane Background	Lane background subtracted with disk size: 10
Lane Width	7.27 mm

Lane 3

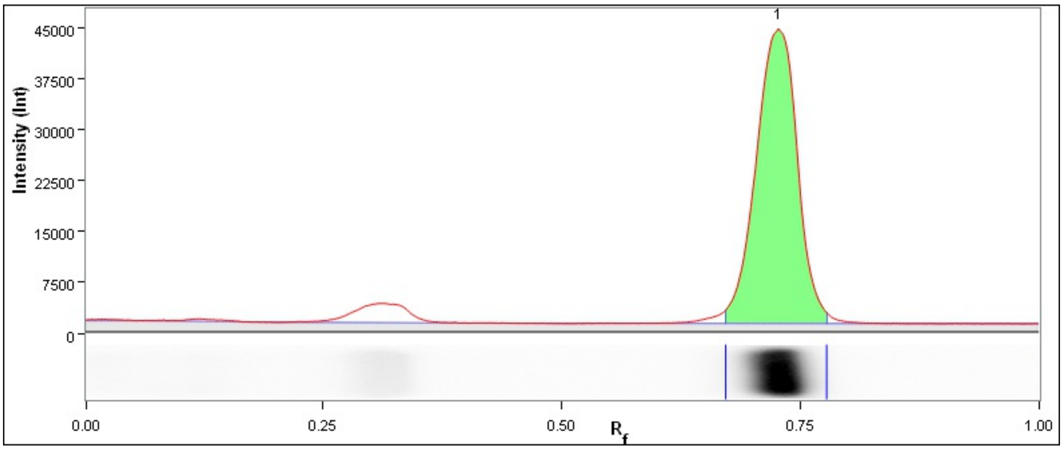

Channel	Band No.	Band Label	Mol. Wt. (KDa)	Relative Front	Adj. Volume (Int)	Volume (Int)	Abs. Quant.	Rel. Quant.	Band %	Lane %	Norm. Factor	Norm. Vol. (Int)
Chemi Hi Resolution	1		N/A	0,728	62.494.073	66.285.589	N/A	N/A	100,0	88,5	N/A	N/A

Band Detection	Automatically detected bands with sensitivity: Low
Lane Background	Lane background subtracted with disk size: 10
Lane Width	7.27 mm

Lane 4

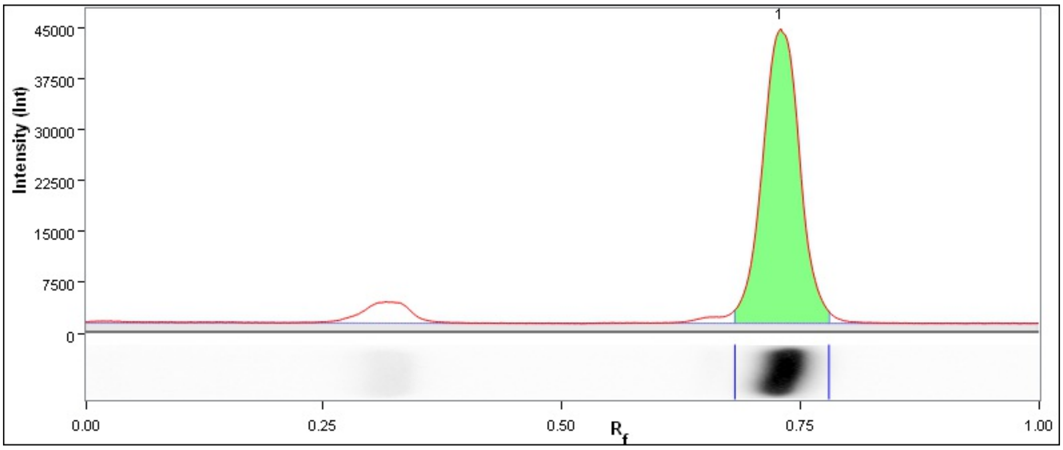

Channel	Band No.	Band Label	Mol. Wt. (KDa)	Relative Front	Adj. Volume (Int)	Volume (Int)	Abs. Quant.	Rel. Quant.	Band %	Lane %	Norm. Factor	Norm. Vol. (Int)
Chemi Hi Resolution	1		N/A	0,731	53.556.048	57.010.112	N/A	N/A	100,0	86,9	N/A	N/A

Band Detection	Automatically detected bands with sensitivity: Low
Lane Background	Lane background subtracted with disk size: 10
Lane Width	7.27 mm

Lane 5

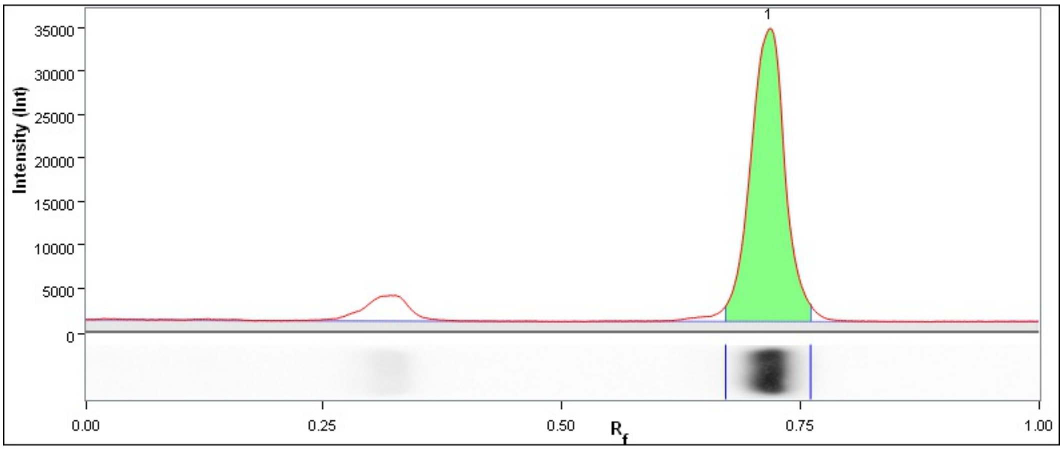

Channel	Band No.	Band Label	Mol. Wt. (KDa)	Relative Front	Adj. Volume (Int)	Volume (Int)	Abs. Quant.	Rel. Quant.	Band %	Lane %	Norm. Factor	Norm. Vol. (Int)
Chemi Hi Resolution	1		N/A	0,719	40.397.372	43.467.746	N/A	N/A	100,0	84,8	N/A	N/A

Band Detection	Automatically detected bands with sensitivity: Low
Lane Background	Lane background subtracted with disk size: 10
Lane Width	7.27 mm

Lane 6

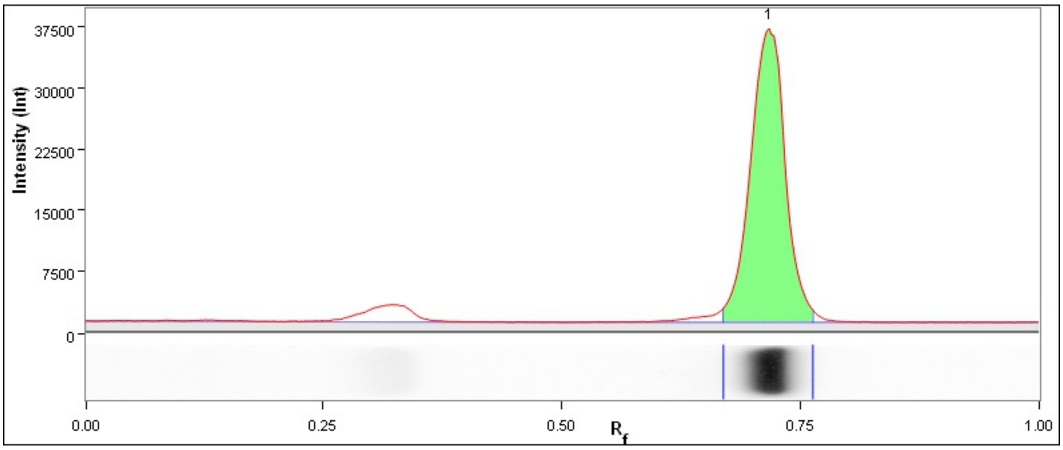

Channel	Band No.	Band Label	Mol. Wt. (KDa)	Relative Front	Adj. Volume (Int)	Volume (Int)	Abs. Quant.	Rel. Quant.	Band %	Lane %	Norm. Factor	Norm. Vol. (Int)
Chemi Hi Resolution	1		N/A	0,719	44.992.258	48.253.074	N/A	N/A	100,0	87,3	N/A	N/A

Band Detection	Automatically detected bands with sensitivity: Low
Lane Background	Lane background subtracted with disk size: 10
Lane Width	7.27 mm

Lane 7

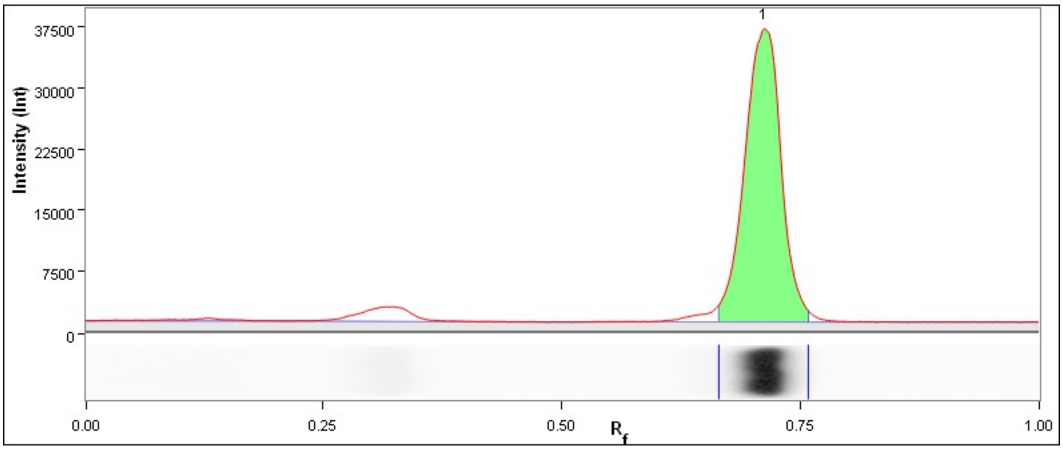

Channel	Band No.	Band Label	Mol. Wt. (KDa)	Relative Front	Adj. Volume (Int)	Volume (Int)	Abs. Quant.	Rel. Quant.	Band %	Lane %	Norm. Factor	Norm. Vol. (Int)
Chemi Hi Resolution	1		N/A	0,714	47.356.374	50.819.466	N/A	N/A	100,0	88,1	N/A	N/A

Band Detection	Automatically detected bands with sensitivity: Low
Lane Background	Lane background subtracted with disk size: 10
Lane Width	7.27 mm

Lane 8

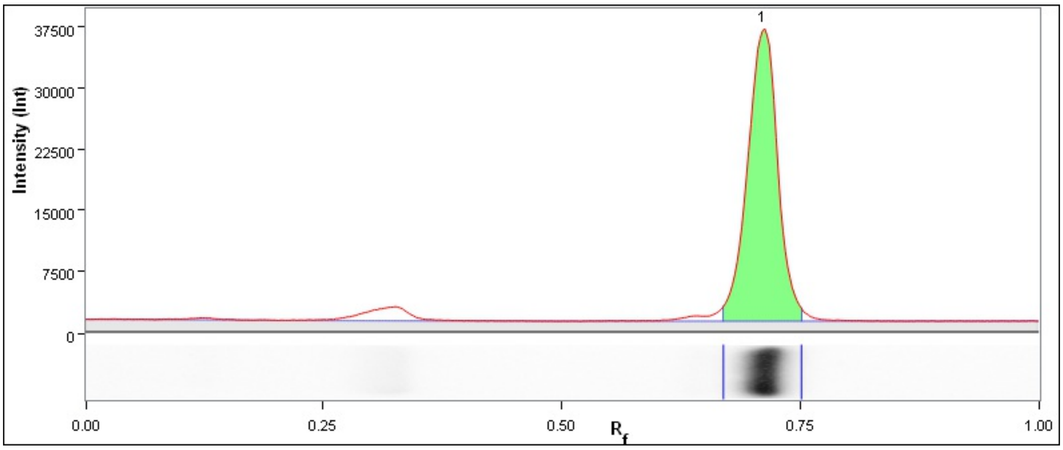

Channel	Band No.	Band Label	Mol. Wt. (KDa)	Relative Front	Adj. Volume (Int)	Volume (Int)	Abs. Quant.	Rel. Quant.	Band %	Lane %	Norm. Factor	Norm. Vol. (Int)
Chemi Hi Resolution	1		N/A	0,712	38.288.053	41.428.638	N/A	N/A	100,0	87,7	N/A	N/A

Band Detection	Automatically detected bands with sensitivity: Low
Lane Background	Lane background subtracted with disk size: 10
Lane Width	7.27 mm

Lane 9

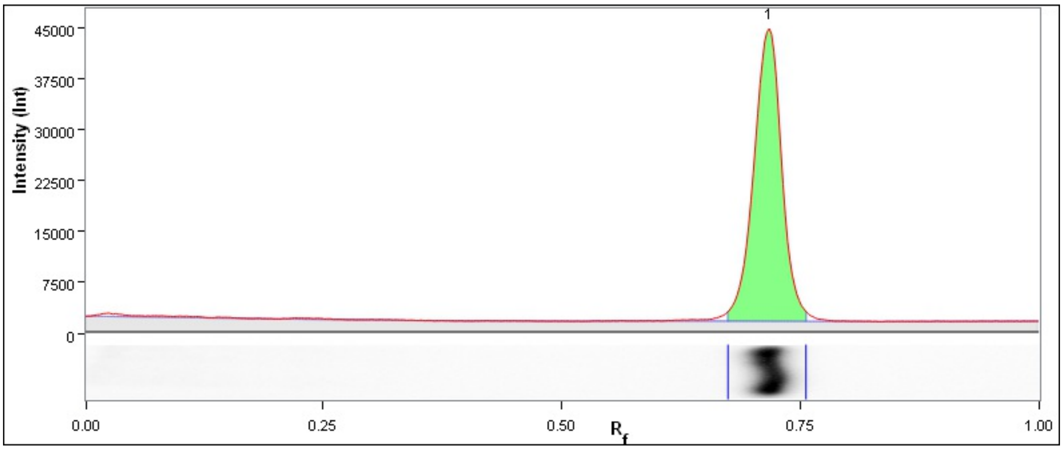

Channel	Band No.	Band Label	Mol. Wt. (KDa)	Relative Front	Adj. Volume (Int)	Volume (Int)	Abs. Quant.	Rel. Quant.	Band %	Lane %	Norm. Factor	Norm. Vol. (Int)
Chemi Hi Resolution	1		N/A	0,719	42.299.328	46.046.848	N/A	N/A	100,0	93,9	N/A	N/A

Band Detection	Automatically detected bands with sensitivity: Low
Lane Background	Lane background subtracted with disk size: 10
Lane Width	7.63 mm

Channel 2 - Green - Chemi Hi Resolution

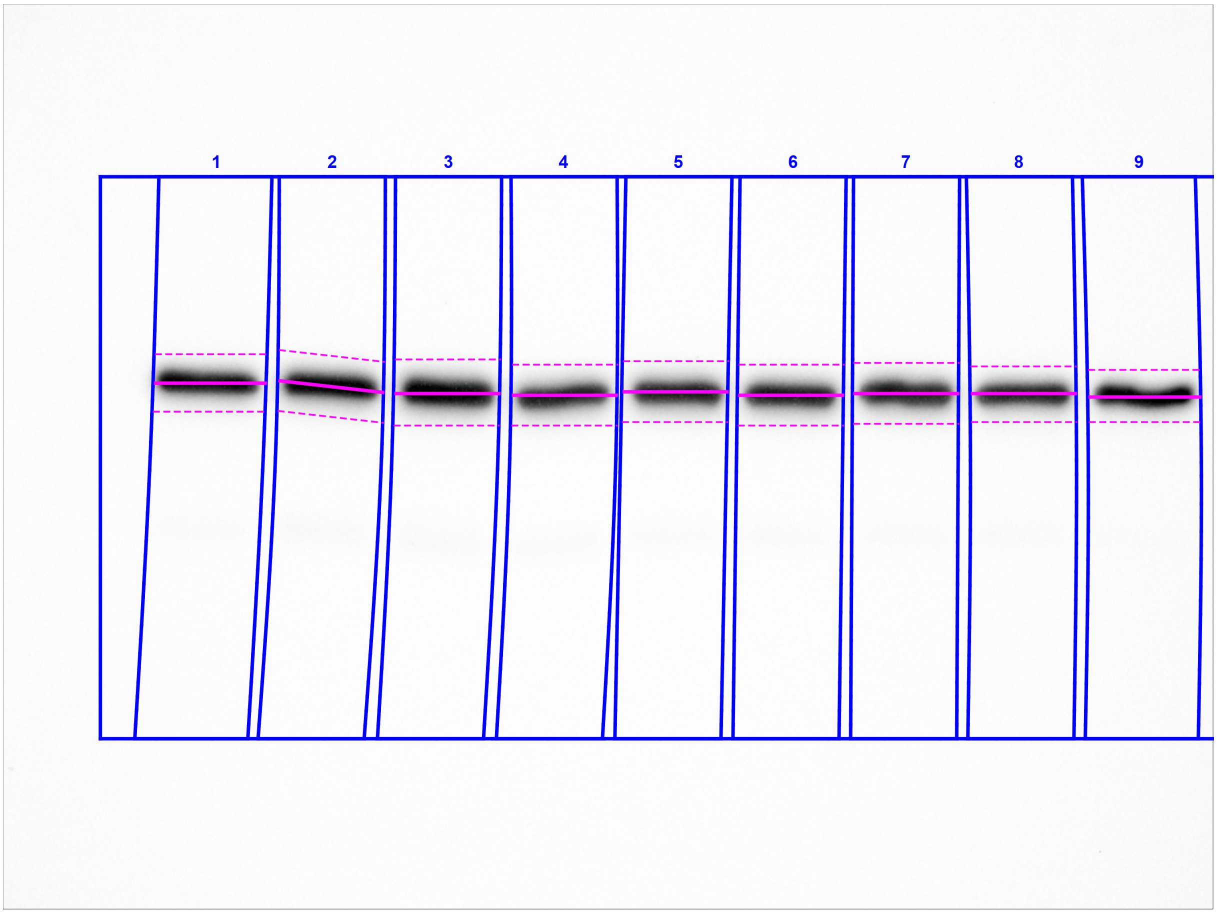

Lane Statistics

Channel	Lane No.	Adj. Total Band Vol. (Int)	Total Band Vol. (Int)	Adj. Total Lane Vol. (Int)	Total Lane Vol. (Int)	Bkgd. Vol. (Int)	Norm. Factor
Chemi Hi Resolution	1	41.383.875	44.219.825	43.104.620	69.702.165	26.597.545	N/A
Chemi Hi Resolution	2	42.894.651	45.557.606	44.904.418	68.653.792	23.749.374	N/A
Chemi Hi Resolution	3	46.938.036	49.718.904	48.972.752	71.957.247	22.984.495	N/A
Chemi Hi Resolution	4	35.596.855	38.127.257	37.436.981	60.124.284	22.687.303	N/A
Chemi Hi Resolution	5	36.295.549	38.853.157	38.071.015	60.892.640	22.821.625	N/A
Chemi Hi Resolution	6	37.316.018	39.910.409	39.131.439	62.457.351	23.325.912	N/A
Chemi Hi Resolution	7	37.909.182	40.622.645	39.719.540	64.016.877	24.297.337	N/A
Chemi Hi Resolution	8	34.845.030	37.488.648	36.836.070	62.656.028	25.819.958	N/A
Chemi Hi Resolution	9	38.705.030	41.679.950	40.397.630	71.339.840	30.942.210	N/A

Lane And Band Analysis

Lane 1

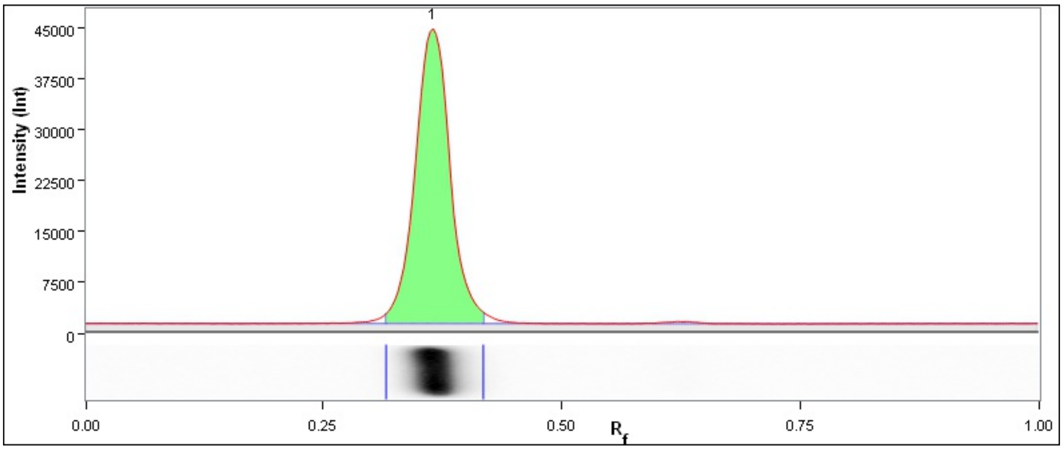

Channel	Band No.	Band Label	Mol. Wt. (KDa)	Relative Front	Adj. Volume (Int)	Volume (Int)	Abs. Quant.	Rel. Quant.	Band %	Lane %	Norm. Factor	Norm. Vol. (Int)
Chemi Hi Resolution	1		N/A	0,367	41.383.875	44.219.825	N/A	N/A	100,0	96,0	N/A	N/A

Band Detection	Automatically detected bands with sensitivity: Low
Lane Background	Lane background subtracted with disk size: 10
Lane Width	7.75 mm

Lane 2

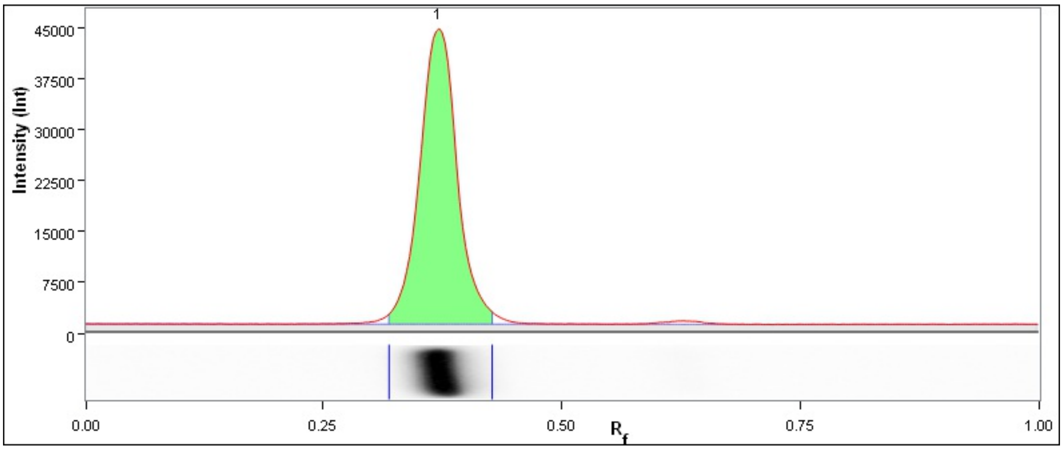

Channel	Band No.	Band Label	Mol. Wt. (KDa)	Relative Front	Adj. Volume (Int)	Volume (Int)	Abs. Quant.	Rel. Quant.	Band %	Lane %	Norm. Factor	Norm. Vol. (Int)
Chemi Hi Resolution	1		N/A	0,373	42.894.651	45.557.606	N/A	N/A	100,0	95,5	N/A	N/A

Band Detection	Automatically detected bands with sensitivity: Low
Lane Background	Lane background subtracted with disk size: 10
Lane Width	7.27 mm

Lane 3

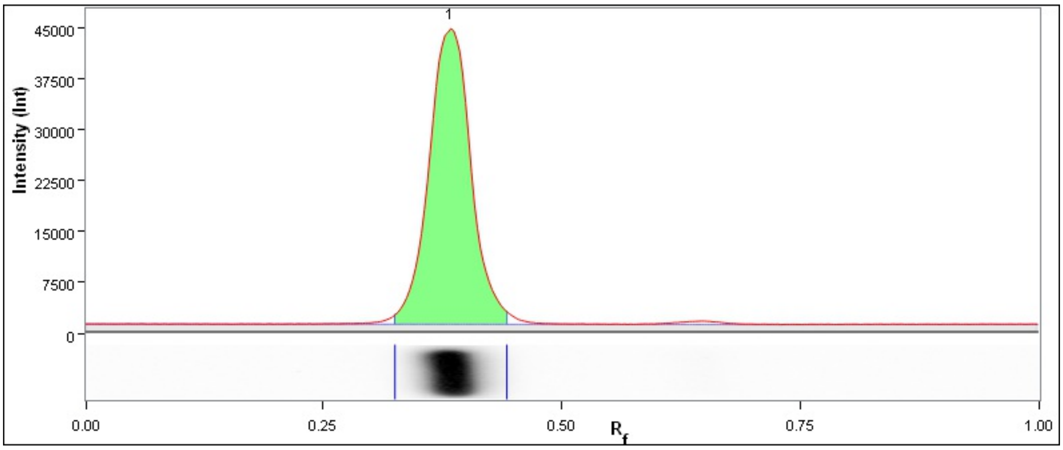

Channel	Band No.	Band Label	Mol. Wt. (KDa)	Relative Front	Adj. Volume (Int)	Volume (Int)	Abs. Quant.	Rel. Quant.	Band %	Lane %	Norm. Factor	Norm. Vol. (Int)
Chemi Hi Resolution	1		N/A	0,386	46.938.036	49.718.904	N/A	N/A	100,0	95,8	N/A	N/A

Band Detection	Automatically detected bands with sensitivity: Low
Lane Background	Lane background subtracted with disk size: 10
Lane Width	7.27 mm

Lane 4

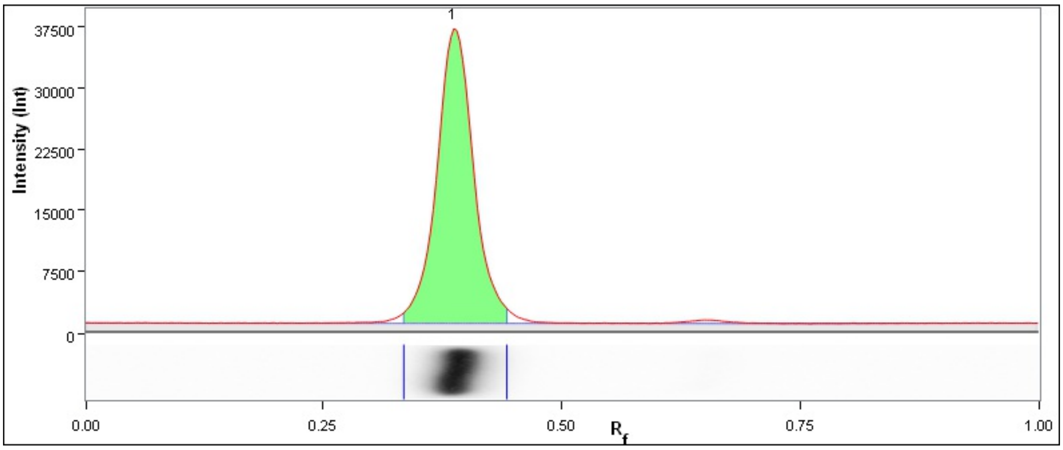

Channel	Band No.	Band Label	Mol. Wt. (KDa)	Relative Front	Adj. Volume (Int)	Volume (Int)	Abs. Quant.	Rel. Quant.	Band %	Lane %	Norm. Factor	Norm. Vol. (Int)
Chemi Hi Resolution	1		N/A	0,389	35.596.855	38.127.257	N/A	N/A	100,0	95,1	N/A	N/A

Band Detection	Automatically detected bands with sensitivity: Low
Lane Background	Lane background subtracted with disk size: 10
Lane Width	7.27 mm

Lane 5

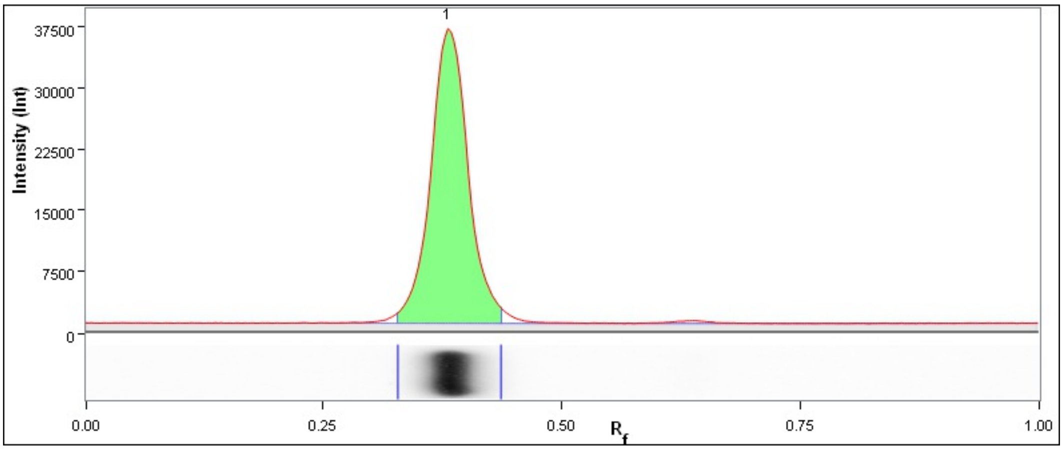

Channel	Band No.	Band Label	Mol. Wt. (KDa)	Relative Front	Adj. Volume (Int)	Volume (Int)	Abs. Quant.	Rel. Quant.	Band %	Lane %	Norm. Factor	Norm. Vol. (Int)
Chemi Hi Resolution	1		N/A	0,383	36.295.549	38.853.157	N/A	N/A	100,0	95,3	N/A	N/A

Band Detection	Automatically detected bands with sensitivity: Low
Lane Background	Lane background subtracted with disk size: 10
Lane Width	7.27 mm

Lane 6

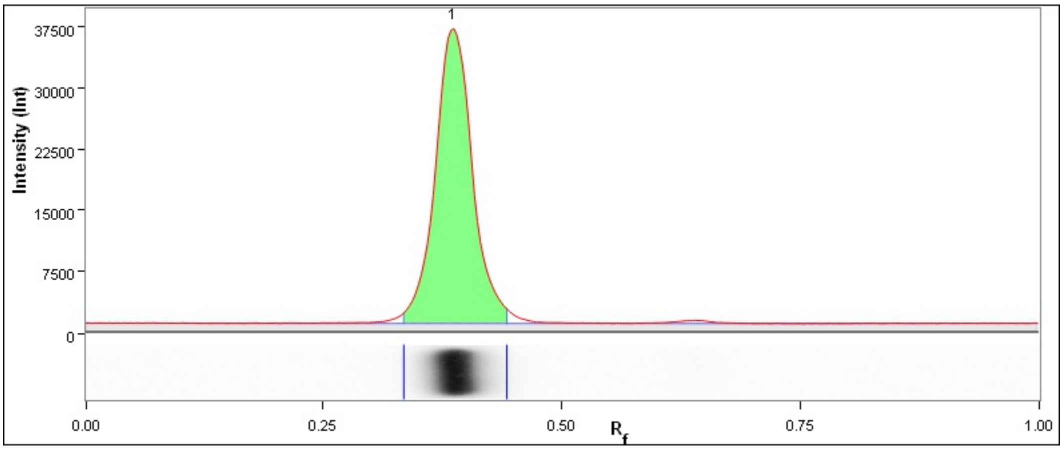

Channel	Band No.	Band Label	Mol. Wt. (KDa)	Relative Front	Adj. Volume (Int)	Volume (Int)	Abs. Quant.	Rel. Quant.	Band %	Lane %	Norm. Factor	Norm. Vol. (Int)
Chemi Hi Resolution	1		N/A	0,389	37.316.018	39.910.409	N/A	N/A	100,0	95,4	N/A	N/A

Band Detection	Automatically detected bands with sensitivity: Low
Lane Background	Lane background subtracted with disk size: 10
Lane Width	7.27 mm

Lane 7

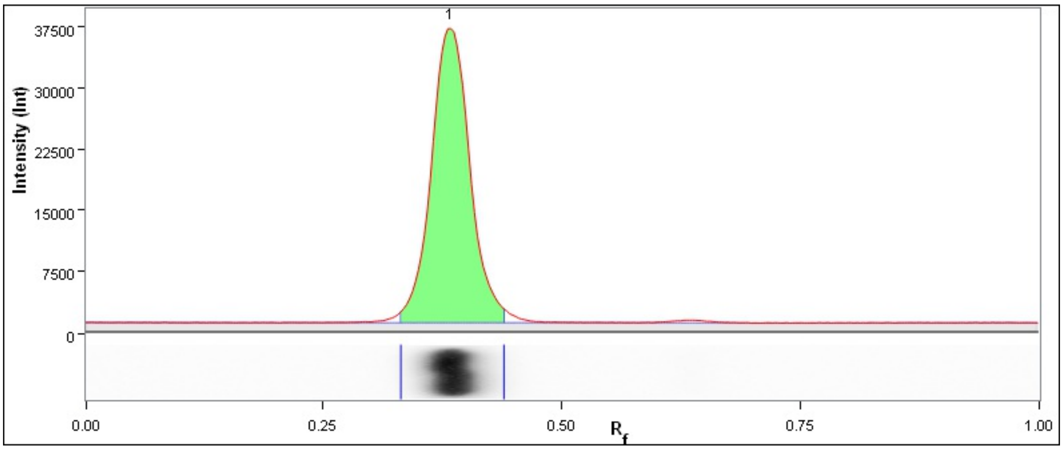

Channel	Band No.	Band Label	Mol. Wt. (KDa)	Relative Front	Adj. Volume (Int)	Volume (Int)	Abs. Quant.	Rel. Quant.	Band %	Lane %	Norm. Factor	Norm. Vol. (Int)
Chemi Hi Resolution	1		N/A	0,386	37.909.182	40.622.645	N/A	N/A	100,0	95,4	N/A	N/A

Band Detection	Automatically detected bands with sensitivity: Low
Lane Background	Lane background subtracted with disk size: 10
Lane Width	7.27 mm

Lane 8

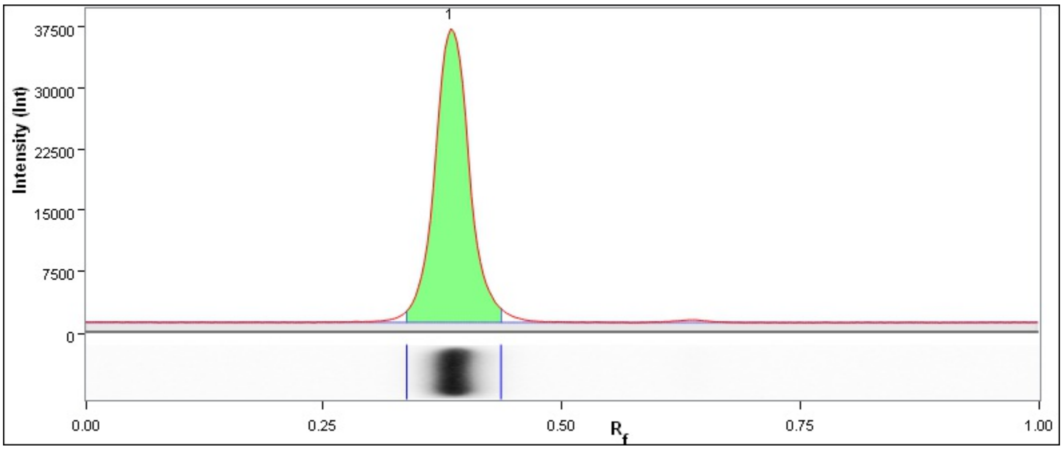

Channel	Band No.	Band Label	Mol. Wt. (KDa)	Relative Front	Adj. Volume (Int)	Volume (Int)	Abs. Quant.	Rel. Quant.	Band %	Lane %	Norm. Factor	Norm. Vol. (Int)
Chemi Hi Resolution	1		N/A	0,386	34.845.030	37.488.648	N/A	N/A	100,0	94,6	N/A	N/A

Band Detection	Automatically detected bands with sensitivity: Low
Lane Background	Lane background subtracted with disk size: 10
Lane Width	7.27 mm

Lane 9

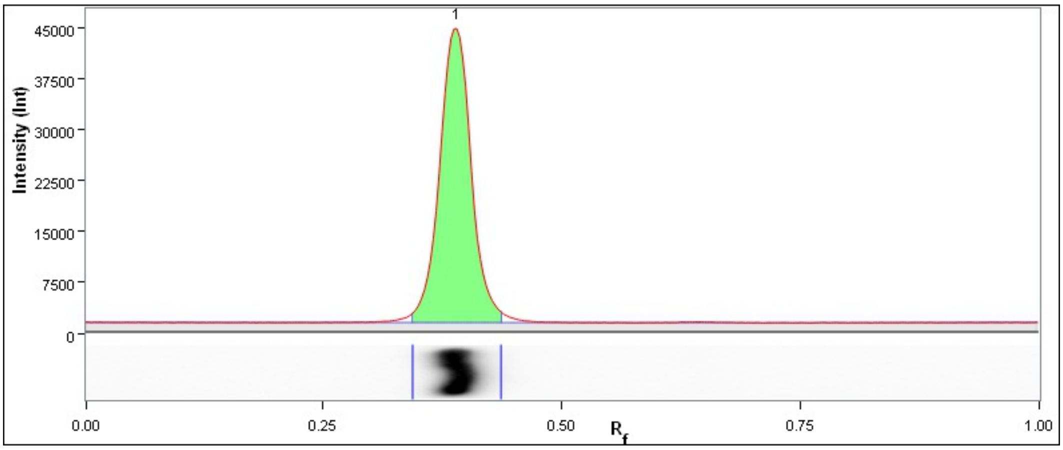

Channel	Band No.	Band Label	Mol. Wt. (KDa)	Relative Front	Adj. Volume (Int)	Volume (Int)	Abs. Quant.	Rel. Quant.	Band %	Lane %	Norm. Factor	Norm. Vol. (Int)
Chemi Hi Resolution	1		N/A	0,392	38.705.030	41.679.950	N/A	N/A	100,0	95,8	N/A	N/A

Band Detection	Automatically detected bands with sensitivity: Low
Lane Background	Lane background subtracted with disk size: 10
Lane Width	7.75 mm
